# C-terminal dimerization motifs control asynchronous chain elongation during modular polyketide biosynthesis

**DOI:** 10.1016/j.jbc.2026.113119

**Published:** 2026-05-06

**Authors:** Chengli Liu, Ryan C. West, Muyuan Chen, Whitaker Cohn, George Wang, Selena Kim, Aryan M. Mandot, Kym F. Faull, Dillon P. Cogan

**Affiliations:** 1 Department of Pharmacology and Pharmaceutical Sciences, University of Southern California, Los Angeles California, USA; 2 Division of CryoEM and Bioimaging, Stanford Synchrotron Radiation Lightsource, SLAC National Accelerator Laboratory, Stanford University, Menlo Park California, USA

**Keywords:** antibiotics, cryogenic electron microscopy, enzymology, natural product biosynthesis

## Abstract

The rifamycin synthetase (RIFS) from the bacterium *Amycolatopsis mediterranei* is a homodimeric assembly line that catalyzes 40+ chemical reactions to generate a complex precursor of the antitubercular drug rifampicin. It consists of an N-terminal substrate loading module followed by a decamodular polyketide synthase (PKS). While the catalytic functions are known for each domain of RIFS, how these activities are spatially and temporally coordinated during polyketide assembly remains incompletely defined. Here, we address this problem with thiol-selective crosslinking to understand the basis for conformational asymmetry during polyketide chain elongation. Our data suggest that C-terminal dimerization motifs—which are ubiquitous in bacterial PKS assembly lines—force their adjacent substrate carrier protein (CP) domains to comigrate between two equivalent ketosynthase (KS) active site chambers. Cryogenic electron microscopy analysis of the first PKS module of RIFS further underscored this observation while revealing its unique architecture. Single-turnover kinetic analysis indicated that although changes to the C-terminus that reduced CP dimerization supported 2-fold greater KS:CP interactions, they were insufficient to overcome substoichiometric product accumulation on the homodimeric protein. Our findings illuminate factors underlying asymmetry during polyketide antibiotic biosynthesis and should be instructive to future megasynth(et)ase engineers.

A variety of uni-cellular and multicellular organisms convert simple metabolic precursors into structurally complex and medicinally important natural products (1). Many of these enzymes, especially those found in bacteria, function as molecular assembly lines wherein groups of multi-enzyme “modules” coordinate stepwise maturation of biosynthetic intermediates across a defined sequence of active sites. The structural and behavioral modularity of these systems presents an encouraging natural platform for directing biosynthesis of custom molecules *via* genetic reprogramming (2). Despite knowledge of hundreds of natural assembly lines that share this general framework (3), universally effective strategies for engineering new biosynthetic pathways while maintaining catalytic integrity remain out of reach. Understanding assembly-line mechanisms will be key to unlocking methods for reliably encoding the biosynthesis of natural product analogs.

Here, we analyze early stages of antibiotic biosynthesis by the rifamycin synthetase (RIFS) in *Amycolatopsis mediterranei* as a model for understanding assembly-line mechanisms in bacteria (Fig. 1*A*). RIFS is a nonribosomal peptide synthetase (NRPS)-polyketide synthase (PKS) hybrid assembly line encoded on five separate genes (*rifA–E*) (4, 5, 6). The overall homodimeric system contains an N-terminal NRPS loading module (LM) followed by 10 PKS elongating modules (M1–M10) (Fig. 1*B*). The adenylation (A) domain of the LM initiates biosynthesis by selecting 3-amino-5-hydroxybenzoate (3,5-AHB) for ATP-dependent arylation of the 4′-phosphopantetheine (Ppant) cofactor of its adjacent substrate carrier protein (CP) domain (Fig. 1*C*) (7). The ketosynthase (KS) domain of M1 then catalyzes transfer of the CP-tethered aryl group onto its catalytic Cys residue, whereupon the intermediate begins repeated elongation and modification by a decamodular PKS (Fig. 1*C*) (8). This basic mechanism for the incorporation of 3,5-AHB by an NRPS LM (consisting of an A-CP didomain), followed by aryl group transfer onto a PKS assembly line, is shared across the structurally diverse family of “ansamycin” natural products (9). Ansamycin assembly lines like RIFS feature embedded acyltransferase (AT) domains, making them “*cis*-AT” type PKSs, as opposed to “*trans*-AT” type PKSs which collaborate with separately encoded, or “standalone”, AT domains (3). The AT domain present in each PKS module catalyzes transfer of an (α-substituted)malonyl group from an acyl coenzyme A metabolite onto the Ppant thiol of its downstream CP domain, thereby supplying the nucleophilic substrate of KS-catalyzed Claisen condensation (elongation) with a KS-bound thioester (Fig. 1*C*). (For an illustration of the catalytic cycle of RIFS LM and M1, see Fig. S1.) Additional modifying domains, such as a ketoreductase (KR), dehydratase (DH), or enoylreductase (ER), may be present in the PKS module and usually catalyze NADPH-dependent reduction (KR/ER), dehydration (DH), or isomerization (KR/DH) of elongated polyketide intermediates before they are received by the downstream module’s KS. Covalent release from RIFS occurs when the M10-bound linear intermediate undergoes macrolactamization to afford proansamycin X, a late-stage precursor of the antibiotic rifamycin B and several of its FDA-approved derivatives (Fig. 1*A*).

**Figure 1:**
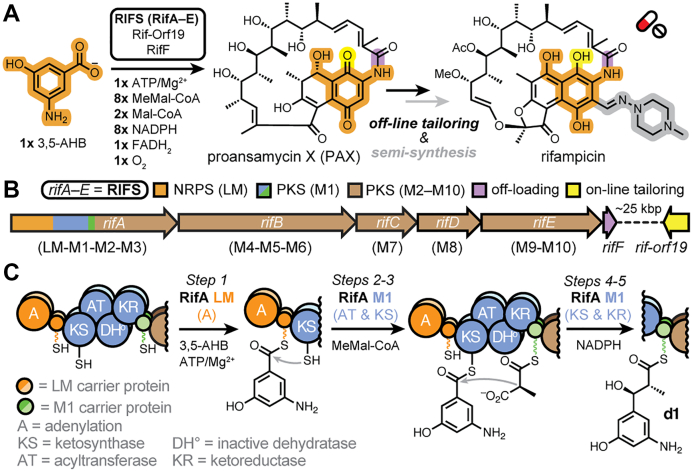
**Overview ofrifamycinbiosynthesis.***A*, biosynthesis of rifamycin precursor, proansamycin X (PAX), by RIFS in partnership with RifF (57) and Rif-Orf19 (58) (Mal = malonyl; MeMal = (2*S*)-methylmalonyl). The antitubercular drug rifampicin is a semisynthetic derivative of PAX-derived rifamycin B. *B*, genetic organization of RIFS (*rifA–E*), *rifF*, and *rif-orf19* in *Amycolatopsis mediterranei*. *C*, biosynthesis of diketide thioester (**d1**) en route to PAX by the LM and M1 of RIFS (*i.e.*, RifA). Two copies of each domain of RifA are shown to depict the homodimeric structure. The stereochemistry of **d1** accords with prior NMR structure elucidation of prematurely released intermediates of RIFS (59). Color-matched squiggly lines attached to sulfur atoms depict 4′-phosphopantetheine (Ppant) groups. The fourth chemical step (catalyzed by the KS) illustrates intermolecular Claisen condensation between protein-tethered reactants from different subunits (distinguished by heavy and light shading).

Given the universality of 3,5-AHB incorporation into the ansamycin natural products and its involvement of a hybrid NRPS–PKS interface (9), we sought to investigate the mechanism of mixed aryl diketide thioester (**d1**) formation by RIFS (Fig. 1*C*). Admiraal *et al.* previously reconstituted **d1** formation *in vitro* from purified substrates and the RIFS LM-M1 bimodule (7, 8, 10). Under single-turnover conditions, they reported that CP arylation by the LM proceeded to a roughly 3-fold greater extent than **d1** formation by M1, implying that only one of the LM-M1 catalytic subunits of the homodimer was fully active (8). Likewise, analysis of the erythromycin synthase determined that its homodimeric PKS modules were only partially (∼50%) occupied with growing polyketide intermediates under steady-state turnover conditions (11). Recent reported structures of PKS modules in asymmetric states support the idea that catalysis in each subunit occurs asynchronously (12, 13, 14, 15). Notwithstanding these advances, an accurate description of the temporal sequence of steps, and their associated subunits, throughout a modular PKS’s catalytic cycle remains incompletely defined.

We recently addressed how the KS domain engages its up- and down-stream CP partners by employing a thiol-reactive crosslinker to trap and measure KS:CP interactions in the erythromycin synthase (15). The reagent 1,3-dibromoacetone (DBA) reacts within seconds with native thiol (or imidazole) groups of the homodimeric protein, thereby promoting site-selective crosslinking between the catalytic Cys (or His) and Ser-Ppant residues of the KS and CP domains, respectively (Fig. 2*A*) (16, 17). For a homodimeric PKS module, 0, 1, or 2 of its KS/CP pairs were predicted to become crosslinked, given the potential for DBA to either covalently link or protect thiol groups based on their proximities (15). (Fig. 2*A* displays one possible outcome following DBA addition to a homodimeric PKS module.) Biochemical and structural analyses confirmed that while an intact PKS module was limited to a single KS-CP crosslink, up to two KS-CP crosslinks could be observed when the CP domains were supplied *in trans* (15). This hinted at a molecular factor for constraining PKS modules to a single KS:CP interaction at a time during polyketide elongation—a phenomenon which has separately been observed in the lasalocid (12) and colibactin (18) assembly lines but, notably, not in structurally related iterative systems which follow a different mechanism (19, 20). Here, we provide a molecular rationale for asynchronous KS:CP interactions in a modular PKS through crosslinking and cryogenic electron microscopy (cryo-EM) analyses of RIFS M1. Our data suggest that dimerization motifs C-terminal to CP domains impose a single KS:CP interaction limit per homodimeric module, as their removal promoted simultaneous crosslinking between both KS/CP pairs. In addition, cryo-EM analysis of M1 resolved its unique architecture and a putative structural transition during the catalytic cycle. Finally, single-turnover kinetic analysis showed that removal of the C-terminal dimerization motif was unable to increase partial product accumulation on the homodimeric LM-M1 bimodule. This observation is consistent with a previously reported mechanism wherein KS-catalyzed elongation is energetically coupled with a conformational change (known as “turnstile closing”) that temporarily inhibits further KS activities in a PKS module (11, 13). Our data add clarity to this mechanistic model by demonstrating that increased CP mobility was insufficient to promote the reactivity of a module that had already completed a catalytic cycle.

**Figure 2:**
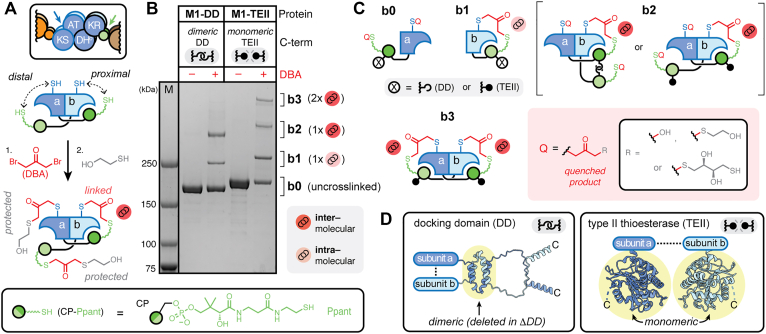
**Proximity-dependent crosslinking of rifamycin synthetase module 1.***A*, addition of DBA to a homodimeric, *holo*-form PKS module (step 1) followed by quenching with excess thiol (step 2) permits proximity-dependent crosslinking or chemical protection of thiols present on KS (*blue*) and CP (*green*) domains. Only one possible crosslinking outcome is shown, and domains not involved in crosslinking have been omitted for clarity. The above, boxed inset shows how the KS and CP domain representation has been adapted here (where ‘a’ and ‘b’ denote PKS subunits); the lower, boxed inset shows the structure of the Ppant posttranslational modification at the conserved Ser residue of the CP domain. *B*, reducing SDS-PAGE analysis following the addition of DBA to M1-DD and M1-TEII, which are RIFS M1 derivatives, revealed a ladder of low-mobility crosslinked bands (**b1**–**b3**) relative to uncrosslinked material (**b0**) (n = 1, technical replicate). *C*, cartoon illustrations of **b0**–**b3** before denaturation by SDS-PAGE. Interlinked *black* hooks illustrate the concept of DD-induced comigration of CP domains. Sulfur atoms attached with ‘Q’ groups depict the quenched reaction product between Cys/Ppant thiols (step 1) and β-mercaptoethanol (BME), dithiothreitol (DTT), or water (step 2) with DBA. DTT was used to quench DBA crosslinking reactions analyzed exclusively by SDS-PAGE, whereas BME was used to quench reactions analyzed by mass spectrometry or cryo-EM. *D*, structural depictions of the C-terminal dimeric docking domain (DD, *black hooks*) from M1-DD and C-terminal monomeric type II thioesterase (TEII, *black circles*) from M1-TEII. The DD model was obtained *via* AlphaFold 3 (43), whereas the TEII model was obtained *via* PDB 3FLA (28).

## Results

To assess the generality of the abovementioned KS-CP crosslinking limit (first observed in PKS modules from the erythromycin assembly line) (15), we isolated the first PKS module (M1) from RIFS as a C-terminally His_6_-tagged recombinant protein for DBA crosslinking analysis. M1 was also fused with short N- and C-terminal docking domains (DDs) from the erythromycin assembly line. The N-terminal DD provided an α-coiled-coil epitope for antibody complexation and cryo-EM analysis, as before (*vide infra*) (13, 21), whereas the C-terminal DD provided an α-helical dimerization motif previously determined to be compatible with expression and purification of functional PKS modules (22, 23). Co-expression of this form of RIFS M1 (M1-DD) with the enzyme Sfp (24, 25) in *Escherichia coli* BAP1 (26) allowed posttranslational 4′-phosphopantetheinylation at Ser1538 of its CP domain from cellular coenzyme A (*i.e.*, conversion from its *apo*- to *holo*-form). Addition of DBA to *holo*-M1-DD followed by SDS-PAGE analysis revealed partial conversion of the starting material into two products, bands 1 (**b1**) and 2 (**b2**), with reduced mobility relative to the uncrosslinked material (**b0**) (Fig. 2*B*) (15, 16). Similarity of **b1** and **b2** to previously crosslinked modules from the erythromycin assembly line (15) suggested that intramolecular and intermolecular KS-CP crosslinks had occurred, respectively (Fig. 2*C*). To confirm the site-selectivity of DBA crosslinking, variants of *holo*-M1-DD whose catalytic KS Cys (C203) residue had been substituted with Ala or Ser were prepared in addition to *apo*-form M1-DD by expressing the wild type (WT) protein in *E. coli* BL21(DE3) (which lacks Sfp). Crosslinking analysis of these M1-DD variants alongside WT *holo*-M1-DD revealed that thiols of the Ppant and catalytic Cys from the CP and KS domains, respectively, were principally involved in the formation of **b1** and **b2** (Fig. S2). Although minor in comparison, the appearance of crosslinked bands similar to **b1** and **b2** in the *holo*-M1-DD(C203(A/S)) variants implied that other nucleophilic residues near the KS active site (*e.g.*, His or Lys) may participate in DBA crosslinking (Fig. S2). The observation that RIFS M1-DD was chemically converted into the above crosslinked products also indicated that the catalytically inactive dehydratase domain (DH°), not present in previously tested PKS modules (15), was not influential in enforcing the apparent single KS-CP crosslinking limit. Thus, asymmetry with respect to intramodular KS:CP interactions appears to be a universal feature of these distinct PKS assembly lines and module architectures.

To understand the basis for this phenomenon, a previous model by Bagde *et al.* was considered that proposed C-terminal dimerization motifs noncovalently hold CP domains in proximity, thereby restricting their simultaneous occupancy at spatially separated KS active sites (12). This model is so far in agreement with available cryo-EM structures of homodimeric PKS modules featuring intramodular KS:CP interactions and a C-terminal dimeric domain (12, 13, 15, 18). Hence, we predicted that replacement of a PKS module’s C-terminal dimeric domain with a monomeric domain might allow both CP domains to interact and crosslink with their KS partners independently. Accordingly, the C-terminal DD of RIFS M1-DD, which is dimeric (27), was substituted with the type II thioesterase (TEII) associated with the rifamycin biosynthetic gene cluster (a.k.a. RifR), which is monomeric (28), affording construct M1-TEII (Fig. 2*B*). Similar isolation of *holo*-form M1-TEII followed by DBA addition and SDS-PAGE analysis again revealed low-mobility bands **b1** and **b2** (with corresponding mass shifts from the larger TEII domain) in addition to a newly observed band (**b3**) with even further reduced mobility (Fig. 2, *B* and *C*). To investigate whether **b3** was formed through a nonspecific reaction with the TEII, a version of M1-DD was prepared featuring a 38-residue deletion corresponding to its dimeric α-helical motif of the C-terminal DD (M1-ΔDD, Fig. 2*D*). DBA addition to M1-ΔDD produced a new band relative to crosslinked M1-DD that migrated similarly to **b3** in M1-TEII, reinforcing the idea that DD dimerization was inhibitory to the formation of this species (Fig. S3). Validation for the crosslinked species was gathered by tandem mass spectrometric analysis of DBA-treated and trypsinized M1-TEII, wherein parent and fragment ions were observed that were consistent with the expected peptides crosslinked at residues Cys203 and Ser-Ppant1538 from the KS and CP domains, respectively (Figs. S4–S6). A similar ladder of low-mobility bands resembling **b1**–**b3** was previously observed following KS-CP crosslinking of the animal fatty acid synthase (FAS), which possesses an overall homodimeric structure and domain architecture like that of modular PKSs (16). Notably, the C-terminal thioesterase domain of animal FAS by itself is monomeric (29). The reported structural assignments of the **b1** and **b2** equivalents in crosslinked animal FAS are similar to those represented in Figure 2*C* and characterized earlier (15). Consistent with the model from Bagde *et al.* (12), the equivalent of **b3** in crosslinked animal FAS was determined to be a crosslinked homodimer bearing two intermolecular KS-CP crosslinks, suggesting similar species were formed after crosslinking M1-TEII and M1-ΔDD (Figs. 2 and S3).

**Figure 3:**
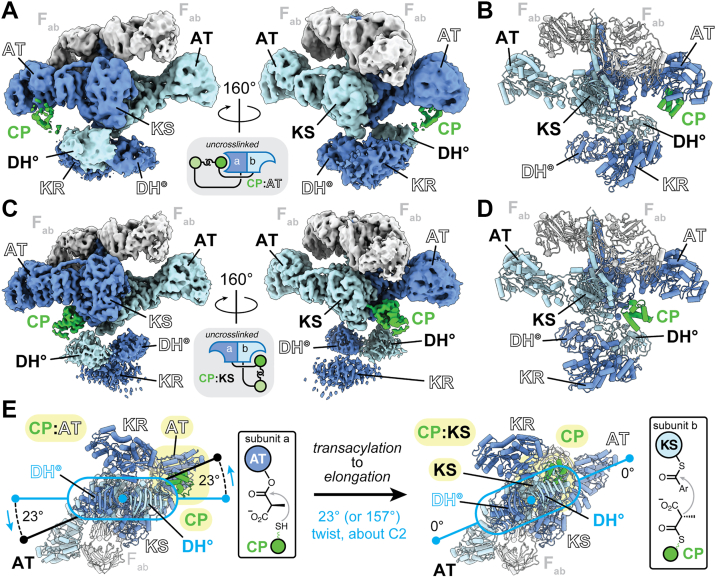
**Cryo-EM structural analysis of *uncrosslinked* rifamycin synthetase module 1.***A* and *B*, the overall cryo-EM map and model of uncrosslinked RIFS M1-DD bound to F_ab_ 1B2 (M1-DD-1B2) in the *transacylation-mode* at 3.96 Å gold-standard Fourier shell correlation (GSFSC) resolution (Figs. S8–S10). Focused refinement produced a supplemental cryo-EM map with more prominent density for the CP domain in the *transacylation-mode* structure (Fig. S11). *C* and *D*, the overall cryo-EM map and model of uncrosslinked M1-DD-1B2 in the *elongation-mode* at 3.22 Å GSFSC resolution (Figs. S8, S12 and S13). A closer view of the associated AT:CP and KS:CP interactions is shown in Figure S14. The cartoon insets in (*A*) and (*C*) arbitrarily represent intermolecular CP:AT or CP:KS interactions, respectively, as similar intramolecular interactions could not be ruled out by the map density. *E*, comparison of the *transacylation*- and *elongation-mode* structures of M1-DD-1B2 highlights potential motion during the catalytic cycle involving a 23° (or 157°) twist of the DH° dimer (or KS-AT dimer) about the pseudo-C2 axis of module symmetry (orthogonal to the plane of the page; represented as a *cyan dot*) (Supporting Movie). Note: “pseudo-C2” in this context implies near-C2 symmetry based on the relatively symmetric KS-AT didomain, whereas “asymmetry” used elsewhere implies an overall lack of symmetry in the homodimer.

To gain structural support of these putative crosslinked states, as well as visualize the unique architecture of M1, our previous methodology for analyzing PKS modules by single-particle cryo-EM was applied to M1-DD (13, 15). The M1-DD protein harbored an N-terminal α-coiled-coil epitope of an antibody fragment (F_ab_) “1B2” previously shown to enhance the particle quality of homodimeric PKS modules (12, 13, 21). The isolated module–F_ab_ complex (M1-DD-1B2, Fig. S7) was subjected to cryo-EM analysis, affording a 2.86 Å consensus map from 1.3 million particles; refined without symmetry (Fig. S8). This map, corresponding to the uncrosslinked state of M1-DD, featured prominent density for two F_ab_ heterodimers bound to the homodimeric KS-AT core (Fig. 3, *A*–*D*). To assess sample heterogeneity, the consensus map and particles were subjected to 3D classification. A majority of the 3D class averages displayed a region of density near the C-terminus of the KS-AT core that resembled previously reported crystal structures of dimeric DHs (30, 31) (Fig. S8). Further map refinement and model building of the M1-DD-1B2 complex supported assignment of this region as the dimeric DH°, thus providing a representative structure of a dehydratase dimer embedded between an AT and KR domain in an intact PKS module. Two noteworthy cryo-EM maps of M1-DD-1B2 emerged from 3D classification and subsequent homogenous refinement (Fig. 3, *A*–*D*). One of them features a single AT:CP interaction (3.96 Å; 37,950 particles), consistent with the conformation during AT-catalyzed extender unit transacylation (*transacylation-mode*, PDB 9PAT; Figs. 3, *A* and *B*, and S8–S11). The other features a single KS:CP interaction (3.22 Å; 177,509 particles), consistent with the conformation during KS-catalyzed chain elongation (*elongation-mode*, PDB 9PAV; Figs. 3, *C* and *D*, S8, 12, and S13). Experimental map-based modeling combined with structural homology analysis supported the existence of electrostatic and hydrophobic interactions nearby and within the KS active site tunnel occupied by the Ppant cofactor (Fig. S14). Importantly, no more than one KS- or AT-bound CP was observed in all 3D class averages of M1-DD-1B2 (Fig. S8). Comparison of the *transacylation-mode* (Fig. 3, *A* and *B*) and *elongation-mode* (Fig. 3, *C* and *D*), structures suggested that their interconversion would be met with a 23° (or 157°) rotation of the DH° dimer (or KS-AT core) relative to the module’s pseudo–C2 axis of symmetry (Fig. 3*E*). Supporting this notion, application of Gaussian mixture model–based heterogeneity analysis measured a trajectory wherein KS-bound CP density was strongest at a relative DH° dimer angle of 0° and weakest at 23° (Fig. 3*E*; Supporting Movie) (32). Assuming that KS-catalyzed elongation occurs between substrates bound to different subunits—in accordance with previous findings (Fig. 1*C*) (13, 33)—the simplest interpretation is that the CP domain interacts intramolecularly with its partner AT to receive a methylmalonyl extender unit before transiting to its nearest KS active site for intermolecular elongation (Fig. 3*E*). Conversely, extender unit transacylation may occur intermolecularly followed by intramolecular elongation, or the two reactions may occur on one or opposite subunits (*i.e.*, both are intramolecular or intermolecular). Further experiments will be required to differentiate among these four possibilities.

**Figure 4:**
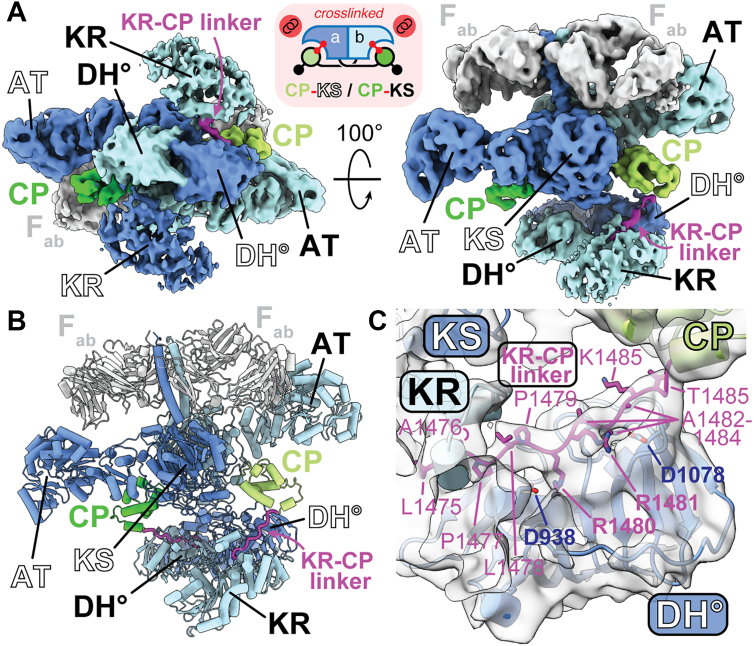
**Cryo-EM structural analysis of *crosslinked* rifamycin synthetase module 1**. *A* and *B*, the overall cryo-EM map and model of crosslinked RIFS M1-TEII bound to F_ab_ 1B2 (CL-M1-TEII-1B2) at 3.96 Å GSFSC resolution (Figs. S15–S17). A notable region of density corresponding to the KR-CP linker is highlighted in *purple*; slightly improved by focused refinement (see Figure S18 for a supplemental cryo-EM map). The equivalent linker in the opposite subunit was not well resolved. *C*, two Arg residues of the KR-CP linker (R1480 and R1481, in *bold*) were predicted to make electrostatic contacts with D938 and D1078 of the DH° domain (in *bold*) from the opposite subunit (Fig. S19). However, site-directed introduction of electrostatic repulsion at these positions followed by kinetic analysis of the protein variants was not supportive of an essential role of these residues for catalysis (Fig. S20).

To interrogate the putative **b3** structure harboring two intermolecular KS-CP crosslinks (Fig. 2, *B* and *C*), DBA-crosslinked M1-TEII bound to 1B2 (CL-M1-TEII-1B2) was prepared for similar cryo-EM analysis. Prior to sample vitrification, gel-filtration and SDS-PAGE analyses supported the existence of a crosslinked module–F_ab_ complex consisting of **b0**–**b3** (Fig. S7). Roughly 45% of the total particles of CL-M1-TEII-1B2 produced a cryo-EM map (3.96 Å; 91,575 particles) featuring unambiguous density for both CP domains bound with their respective KS partners (PDB 9PC6; Figs. 4, *A* and *B* and S15–S17). Although poor local resolution in the KS active site precluded direct measurement of KS-CP crosslinks, a fraction of the sample was inferred to be crosslinked at this site based on the above mutational (Fig. S2) and mass spectrometry (Figs. S4–S6) data. Therefore, DBA-dependent formation of **b3** in M1-TEII (but not in M1-DD) was attributed to its absence of a C-terminal dimerization motif which promoted two simultaneous KS:CP interactions (Fig. 2, *B* and *C*). A similar 3D class average contained density for only one KS-bound CP domain, consistent with the additional presence of **b1** and **b2** in the crosslinked sample mixture (Figs. S7 and S15, class 3). The cryo-EM map of CL-M1-TEII-1B2 also highlighted an unexpected interaction between the unstructured KR-CP linker and a shallow groove of the opposite subunit’s DH° domain (Figs. 4*C* and S18). Therein, putative electrostatic contacts occur between Arg1480/Arg1481 of the KR-CP linker and Asp938/Asp1078 of the DH°, respectively. Although three out of four of these residues are conserved in PKS modules with similar domain architectures (Fig. S19), genetic mutations at these sites did not inhibit diketide formation by the LM-M1 bimodule (see below for associated kinetic analysis and Fig. S20).

Overall, cryo-EM analysis of CL-M1-TEII-1B2, which featured 2-fold (pseudo-C2 symmetric) KS:CP interactions, provided further support of the hypothesis that dimerization motifs at the C-termini of PKS modules act to simultaneously confine both CP domains in proximity to a single KS active site. Considering that the DD may have introduced a behavioral artifact in M1-DD, given its affiliation with a different assembly line, a version of M1 was created that was C-terminally fused with the KS-AT didomain from RIFS module 2 (M2). The homodimeric nature of the KS-AT didomain was expected to restrict CP mobility analogous to the effect of the DD while providing M1 with a sizeable fragment (∼95 kDa) of its natural C-terminus. DBA addition to a variant of this protein (genetically modified to remove the catalytic Cys in the second KS domain) produced a crosslinking pattern consistent with the equivalents of **b1** and **b2**, but not **b3**. This assignment was further interrogated by including a standalone, fluorescent CP probe during DBA crosslinking reactions which served as a beacon for unoccupied KS active sites, as described earlier (Fig. S21) (15). Thus, dimerization of the KS-AT, a native element of the RIFS M1 C-terminus, also appears to enforce a single, intramodular KS-CP crosslinking event.

To explore the generality of this mechanism, we performed a similar crosslinking analysis on a module from the nocardiosis-associated PKS assembly line derived from the *Nocardia* genus (34). DBA treatment of this protein harboring a C-terminal DD or TEII revealed a strong dependence of its **b3** equivalent on TEII, suggesting that the effect of C-terminal dimerization motifs on KS–CP interaction stoichiometry is not unique to RIFS (Fig. S22). Moreover, this behavior appears to be independent of the type of dimerization motif, as multiple C-terminal domains have been associated with singular KS:CP interactions: namely, (i) the “post-AT dimerization element” present in Lsd14 (12), (ii) the dimeric type I thioesterase of erythromycin synthase (13, 15, 35), (iii) the dimeric α-helical DD of erythromycin synthase M2 (13, 15, 27), and (iv, reported here) the dimeric KS-AT fragment of RIFS M2 (Fig. S21). A recent publication also highlighted single and double KS occupancy by CP domains in PKS modules from the colibactin assembly line (18). Importantly, the observed 2-fold KS:CP interactions correlated with the absence of a C-terminal dimerization motif.

Having established a plausible basis for singular KS occupancy by a downstream CP domain in a homodimeric PKS module, the function of this behavior was questioned in the context of the PKS’s catalytic cycle. We considered that the C-terminal dimerization motif of M1-DD might prevent it from utilizing both subunits concurrently. From this, it was hypothesized that the presence of a C-terminal dimerization motif would only allow complete polyketide processing in one catalytic subunit, whereas its absence would permit complete processing in both. If true, then M1-TEII would be expected to produce two aryl diketide thioester (**d1**) products following substrate incubation (without product release from the module), whereas M1-DD would only produce one (Fig. 1*C*). As expressed above, evidence for partial module occupancy (*i.e.*, ≤1 product per two subunits) has been previously observed in PKS modules from both the rifamycin (8) and erythromycin (11) assembly lines. Notably, each of them harbored a C-terminal dimeric domain.

**Figure 5:**
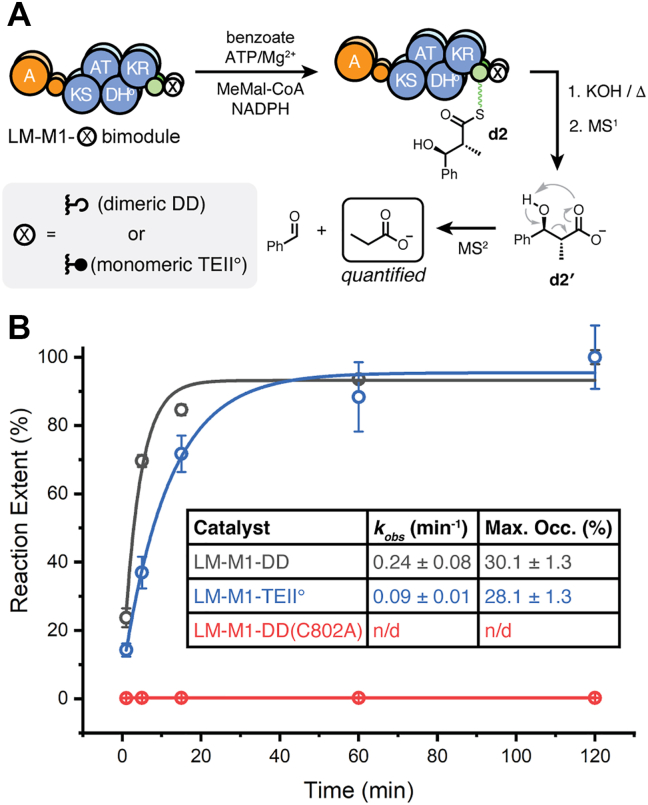
**Single-turnover kinetic analysis of a rifamycin synthetase hybrid bimodule.***A*, enzymatic production of covalently anchored diketide (**d2**) by the RIFS LM-M1-X bimodule, where X denotes either a C-terminal DD or TEII°. Following the reaction, the enzyme-bound diketide was released by KOH treatment and heat to generate **d2′**, which was quantified by LC-MS/MS in negative ion mode using MRM. Quantitation was based on the transition from the parent ion (C_10_H_11_O_3_^–^, *m/z* 179) to the propionate fragment (*m/z* 73; boxed in *gray*), with the *m/z* 117 fragment used as a qualifier ion, at a defined retention time (Fig. S23). *B*, time-dependent formation of **d2′** is shown for LM-M1-DD, LM-M1-TEII°, and LM-M1-DD(C802A), a KS-inactivated variant. Data are plotted as normalized reaction extents based on MRM transition intensities. The C802A substitution in LM-M1-DD corresponds to C203A in M1-DD. Curves were fit to a single-exponential growth function to obtain apparent rate constants (*k*_*obs*_). Error bars represent the SD of three technical replicates (n = 3). Quantification of **d2′** standards in the presence of LM-M1-DD or LM-M1-TEII° was used to calculate the maximum fractional occupancy of each protein with **d2** in enzymatic reactions (Fig. S23).

To test this prediction, a kinetic assay was developed to measure the concentrations of module-bound **d1**. Admiraal *et al.* previously demonstrated that, with the addition of ATP/Mg^2+^, methylmalonyl coenzyme A (MeMal-CoA), and NADPH substrates, the RIFS LM-M1 bimodule converts benzoate, a simple analog of the native starter unit, into 3-hydroxy-2-methyl-3-phenylpropanoyl-CP thioester (**d2**), the corresponding diketide analog of **d1** (Figs. 1*C* and 5*A*) (8). Using this strategy, and with the authentic hydrolyzed product (**d2′**) as a reference, we measured the concentrations of **d2′** in base- and heat-treated hydrolysates of enzymatic reactions by liquid chromatography-negative ion tandem mass spectrometry with multiple reaction monitoring (LC-MS/MS-MRM). We then purified recombinant forms of the RIFS LM-M1 bimodule C-terminally fused with DD or TEII to measure the influence of C-terminal domains on enzymatic **d2** formation. In these experiments, an inactive variant of the TEII domain was used—created by replacing its active site Ser with Ala (denoted TEII°) (28)—to prevent potential enzymatic release of **d2** and therefore ensure that the protein was limited to a single catalytic cycle per active subunit. To minimize possible steric effects of TEII° on enzymatic activity, a longer (G_4_S)_8_ linker between the CP and TEII° domains relative to M1-TEII was also employed in LM-M1-TEII°. DBA crosslinking reactions indicated that these changes remained compatible with **b1**–**b3** production and thus 2-fold KS:CP interactions (Fig. S3). Enzymatic production of **d2** was initiated by combining *holo*-form LM-M1-DD or LM-M1-TEII° with benzoate, ATP/Mg^2+^, MeMal-CoA (racemate), and NADPH and quenched at various time points from 1 min to 2 h by the addition of KOH and heating (Fig. 5*A*). LC-MS/MS-MRM quantification of **d2′** over time confirmed that both LM-M1-DD and LM-M1-TEII° were catalytically active, whereas LM-M1-DD(C802A), which lacked the essential Cys of the KS domain (equivalent to C203A above), exhibited no detectable activity (Fig. 5*B*). The maximum concentration of **d2′** approached roughly 30% of the concentration of protein in the LM-M1-DD–containing reaction (Figs. 5*B* and S23), recapitulating the observation that protein-bound diketide (**d2**) accumulated substoichiometrically prior to hydrolysis (despite presence of >250 equivalents of substrate) (8). Interestingly, a nearly identical maximum diketide occupancy was observed in reactions containing LM-M1-TEII° (Figs. 5*B* and S23). Thus, contrary to our prediction, the extent of **d2** formation did not increase when the C-terminal dimeric domain of the PKS module was replaced with a monomeric domain. This implied that the C-terminal dimerization motif of LM-M1-DD was not the sole determinant of incomplete polyketide processing by the homodimeric protein.

The observation that LM-M1-DD catalyzed **d2** formation approximately 2- to 3-fold faster than LM-M1-TEII° was also unexpected, considering that TEII° should have granted an increase in KS:CP interactions based on the crosslinking data (Figs. 2*B* and S3). We therefore isolated the rates of M1-catalyzed reactions by pre-incubating LM-M1-DD or LM-M1-TEII° with benzoate and ATP/Mg^2+^ to accumulate equal concentrations of the benzoylated protein prior to the addition of MeMal-CoA and NADPH cosubstrates. Similar kinetic analysis of **d2′** production again revealed that LM-M1-DD outperformed LM-M1-TEII° with an approximately 2-fold greater rate constant (Fig. S24), suggesting that TEII° and/or its associated (G_4_S)_8_ linker were inhibitory to the catalytic activities of M1. Together, our data indicate that LM-M1-DD catalyzed **d2** formation faster than LM-M1-TEII° but up to the same fractional occupancy, despite the ability of the TEII° domain to confer an increased proportion of KS:CP interactions in the PKS module.

Lastly, considering that LM-M1 might be capable of catalyzing two rounds of polyketide elongation but only a single round of KR-catalyzed ketoreduction en route to **d2**—which would also align with the observed substoichiometric (∼30%) product occupancy of LM-M1—an authentic standard of the unreduced β-keto version of **d2** (**keto-d2**) was synthesized for similar kinetic and occupancy studies (Fig. S25). While the expected time- and NADPH-dependent accumulation of hydrolyzed **keto-d2** (**keto-d2′**) was observed, instability of the β-keto acid under elevated pH and temperature required for diketide release prevented its accurate quantification (Figs. S26 and S27). In any case, previous measurements of substoichiometric occupancy argued against a model for simultaneous attachment of both reduced and unreduced intermediates on a PKS module, as the radioisotope-labeling strategy permitted unbiased detection of both forms of the growing polyketide (11).

## Discussion

This analysis of the rifamycin assembly line provides a molecular basis for previously observed conformational asymmetry in homodimeric PKS modules during KS-catalyzed C–C bond formation (12, 13, 18). In each of these cited cases, PKS modules bearing C-terminal domains with dimerization motifs were employed. We showed with protein crosslinking and single-particle cryo-EM that replacement of a C-terminal dimeric domain (α-helical DD or KS-AT didomain) with a monomeric domain (TEII or ΔDD) promoted up to 2-fold elevated KS:CP interactions per PKS module (Fig. 2). These findings substantiate a prior proposal by Bagde *et al.* that CP domains of a PKS module swing together like a pendulum between two equivalent KS active site chambers (12). Evidently, this pendulum is stabilized by C-terminal dimerizing forces that can be removed through protein engineering to create two pendulums that migrate independently.

Cryo-EM analysis of RIFS M1 provided snapshots of a PKS module bearing a KS-AT-DH°-KR-CP domain architecture. The characteristic shape of the DH° dimer could be readily detected in cryo-EM maps of RIFS M1, which in turn provided a tracer for measuring a putative twisting motion at the KS-AT/DH°-KR-CP interface during the catalytic cycle (Fig. 3*E*, Supporting Movie). These structures highlight the conformational plasticity of the module while unveiling a plausible structural change during the transition between two, asymmetric catalytic states.

Having established a structural basis for asymmetric KS-CP engagement, we next asked how these constraints relate to the previously reported partial product occupancy of PKS homodimers. The results of single-turnover kinetic analysis of the RIFS LM-M1 bimodule argued that even when both CP domains independently engage their KS partners—by virtue of removing dimeric elements that otherwise hold them together—only one of them facilitates the complete set of reactions leading to the diketide product (**d2**) (Fig. 5). Thus, increased KS:CP encounter frequency alone was insufficient to overcome the observed substoichiometric product occupancy, implying that additional regulatory features beyond CP mobility control the limited extent of polyketide processing by a homodimeric PKS.

One explanation consistent with these observations is that KS-catalyzed elongation in one catalytic subunit transiently suppresses the activities of its neighboring subunit through allosteric coupling. This interpretation is compatible with a previously proposed “turnstile” mechanism in which KS-catalyzed elongation induces a conformational change that sterically restricts substrate entry into both KS active sites (11, 13) (Fig. S1). In the current work, we show that increasing the migrational independence of CP domains—while it enhances KS-CP crosslinking—does not increase product occupancy. This finding places constraints on mechanistic models for asynchronous polyketide biosynthesis and is consistent with the notion that catalytic activities in one subunit may transiently disengage the other. Another possible explanation for the partial product occupancy is that asymmetric conformational states induced by neighboring catalytic domains and/or interdomain linkers bias catalysis toward the reaction chamber of a single catalytic subunit.

Although the present study focuses on the first module of the rifamycin assembly line, several observations suggest that the findings may apply broadly across modular PKSs. First, singular KS occupancy by CP domains was previously observed in PKS modules from the erythromycin, lasalocid A, and colibactin assembly lines. Second, this behavior is associated with a variety of structural motifs, each of which contributes a dimerization interface. Here, we add to this list of motifs through crosslinking analysis of RIFS M1 appended to its native KS-AT didomain of RIFS M2. We also performed crosslinking analysis of a PKS module from the nocardiosis-associated polyketide assembly line, lending credence to the generality of this mechanism across a variety of systems.

While theoretically introducing TEII° into the C terminus of LM-M1 could have increased the extent and/or rate of diketide formation—given its promotion of 2-fold higher KS:CP interactions—no effect on the extent and rather a reduced rate was observed, relative to reactions with LM-M1 terminating in a more natural dimeric DD (Fig. 5). This finding may reflect an evolved preference of multifunctional PKS modules to utilize one catalytic subunit at a time for increased fidelity at the expense of efficiency, unlike their monofunctional animal FAS counterparts which employ both subunits simultaneously to achieve extremely rapid rates of catalysis (16, 36, 37, 38).

Overall, this work deepens our understanding of molecular factors that control asynchronous C–C bond formation during assembly-line polyketide biosynthesis in bacteria. Future studies will be necessary to illuminate the full motion pictures of these molecular factories at work.

## Experimental procedures

### Materials

Bacterial growth media, enzymes, purification resins, Amicon Ultra Centrifugal Filters, and other chemicals were purchased from Thermo Fisher Scientific, Avantor (VWR International), or Millipore Sigma. Antibiotics, IPTG, DTT, and tris-(carboxyethyl) phosphine (TCEP) were purchased from Gold Biotechnology. (2*R*,3*S*)-3-hydroxy-2-methyl-3-phenylpropanoic acid (**d2′**, Fig. 5*A*) was purchased from Enamine (EN300-8130951). Cryo-EM grids were purchased from Electron Microscopy Sciences or MiTeGen. Polyacrylamide gels were purchased from Thermo Fisher Scientific or Bio-Rad. DNA oligonucleotides were purchased from Integrated DNA Technologies, and DNA sequencing services were provided by Azenta Life Sciences. Microbial strains used in this work were provided by the USDA-ARS Culture Collection (NRRL).

### General methods

Protein purification was carried out using an ÄKTA Pure chromatography system (Cytiva Life Sciences). Protein concentrations were determined either by (1) absorbance measurements at 280 nm using calculated molecular weights and extinction coefficients or (2) *via* the Bradford assay with bovine serum albumin standards and absorbance measurements at 595 nm. Absorbance at 280 nm was performed using the nanostage (pedestal) of a NanoDrop OneC (Thermo Fisher Scientific), whereas absorbance at 595 nm was performed using the 1 cm cuvette holder in the same device (39). Gels were imaged on an iBright FL1000 gel imager (Invitrogen), Bio-Rad ChemiDoc Imaging system, or with an iPhone 13 mini (Apple Inc.)

### Genomic DNA extraction

*A. mediterranei* NRRL B-3240 obtained as a freeze-dried pellet was streaked onto a plate of ISP Medium No. 4 (HiMedia) and grown at 28 °C for 4 days. The resulting colonies were scraped from the surface of the plate, and genomic DNA therein was extracted using the E.Z.N.A. Bacterial DNA Kit (Omega Bio-tek).

### Production of plasmids for protein overexpression

DNA oligonucleotides (0.3 μM) and template (4 ng/μl: gDNA; 0.4 ng/μl: plasmid DNA) were used to amplify the protein-coding regions with CloneAmp HiFi PCR Premix (Takara Bio USA) supplemented with 1 M betaine and 3% (ν/ν) dimethyl sulfoxide (DMSO) by employing a touchdown PCR protocol (40). Amplicons were analyzed and purified by agarose gel electrophoresis and ligated with similarly prepared pET21- or pET28-derived DNA fragments *via* In-Fusion Cloning (Takara Bio USA) to create circular DNA plasmids (Table S3). Initial plasmid products were used to transform *E. coli* Stellar competent cells (Takara Bio USA), and the resulting transformants were grown (12 h, 37 °C, 220 rpm) in 5 to 10 ml LB (Miller) broth supplemented with appropriate antibiotic (100 μg/ml carbenicillin or 50 μg/ml kanamycin). Plasmids were isolated from the liquid cultures *via* EasyPrep (Bioland Scientific LLC) and verified by Sanger or whole plasmid sequencing prior to downstream use.

### Protein expression and purification

Purification of the F_ab_ 1B2 was performed as before (13). For purification of all other proteins, expression plasmids (Table S3) were used to transform *E. coli* BL21(DE3) or BAP1 (26) competent cells to generate proteins in their *apo* or *holo* forms, respectively. Single colony transformants were selected to inoculate 10 to 15 ml LB (Miller) broth supplemented with appropriate antibiotic (100 μg/ml carbenicillin or 50 μg/ml kanamycin) and grown for 14 h (37 °C, 220 rpm). Two milliliters of the resulting cell suspensions were used to inoculate every 1 L of similar LB + antibiotic medium and incubated (37 °C, 220 rpm) until an absorbance at 600 nm (A_600_) of ≈0.5 was achieved (3–6 L total). Cell cultures were cooled for 15 min in an ice bath before addition of IPTG (0.25 mM final concentration) and continuing incubation (18 °C, 220 rpm, 18–20 h). Cells were harvested by centrifugation at 3000*g* for 30 min and resuspended in 5 ml of 0.45 M NaCl, 10 mM imidazole, 50 mM NaH_2_PO_4_, 20% glycerol, pH 7.8 (NaOH) per liter of cell culture. Cells were lysed by sonication (Branson Sonifier 450), and lysates were clarified by centrifugation at 23,400*g* in a Sorvall RC 5B centrifuge. Supernatants were added to HisPur Ni-NTA resin (Thermo Fisher Scientific) in a glass Econo-Column (Bio-Rad) equilibrated with 0.3 M NaCl, 50 mM imidazole, 50 mM NaH_2_PO_4_, 10% glycerol, pH 7.8 (NaOH) (wash buffer) at a ratio of 1 ml resin per liter of cell culture. The protein-bound resin was washed with 100 ml of wash buffer in 4 × 25 ml increments before adding 50 ml of 40 mM NaCl, 500 mM imidazole, 50 mM NaH_2_PO_4_, 10% glycerol, pH 7.6 (NaOH) in 4 × 12.5 ml increments to elute the bound protein. (Note: it was important to maintain NaCl at ≤50 mM in the eluant to maximize protein binding in the subsequent step.) The eluant was applied onto a 5 ml HiTrap Q HP anion exchange chromatography column (Cytiva Life Sciences) equilibrated with 5 mM 4-(2-hydroxyethyl)-1-piperazineethanesulfonic acid (Hepes), 50 mM citric acid, 10% glycerol, pH 7.6 (NaOH) (low-salt buffer) then washed with 50 ml of low-salt buffer before employing a 0 to 60% linear gradient of increasing 5 mM Hepes, 50 mM citric acid, 1 M NaCl, 10% glycerol, pH 7.6 (NaOH) (high-salt buffer) over 60 ml while collecting 3 ml fractions at a flow rate of 2 ml/min. For all RIFS protein variants, the major peak eluted at ≈0.3 M NaCl. Protein purity was assessed by SDS-PAGE, and purified protein fractions were pooled and concentrated in 30 kDa molecular weight cutoff (MWCO) Amicon Ultra Centrifugal Filters. Protein concentrated to ≤1 ml was injected onto a 120 ml Superdex 200 pg 16/600 column (Cytiva Life Sciences) for size-exclusion chromatography (SEC) and eluted isocratically with 0.1 M citric acid, 0.1 M NaCl, 10 mM Hepes, pH 7.2 (NaOH) (SEC buffer) while collecting 3 ml fractions at a flow rate of 1 ml/min. For protein samples that were subjected to single-particle cryo-EM analysis, the same buffer lacking 0.1 M NaCl was used during SEC purification, and fractions were collected manually to avoid undesired aggregated forms of the protein that eluted around 45 to 50 ml (Fig. S7*B*). Proteins purified by SEC were concentrated to ≈5 to 10 mg/ml in Amicon Ultra Centrifugal Filters (30 kDa MWCO) and, if not used immediately for experimentation, flash-frozen in ≤100 μl aliquots by immersion in liquid nitrogen (LN2) for storage at −80 °C.

### General DBA crosslinking

Prior to use, neat DBA (2 ml) was filtered through a small plug (≈0.5 ml) of anhydrous aluminum oxide in a Pasteur pipette under N_2_ gas and diluted to 0.5 M in anhydrous DMF for storage at −80 °C. This stock solution was diluted to 5 mM in anhydrous DMF and further diluted to 0.25 mM in 50% aqueous DMF to create working solutions for all crosslinking reactions. The working solution was prepared in small quantities (50–100 μl) and used within 15 min. Unless otherwise stated, all crosslinking reactions analyzed by SDS-PAGE were performed as single technical replicates.

### DBA crosslinking of WT and variants of RIFS M1-DD and M1-TEII

*Holo*- or *apo*-form M1-DD or M1-TEII harboring WT or genetically modified sequences were purified according to the above procedure, concentrated to ≈7 mg/ml, and incubated individually at 5 μM with 0.1 mM TCEP in 300 mM citric acid, 20 mM Hepes, pH 7.3 (NaOH) for 15 min before adding 15 μM DBA or an equal volume of 50% aqueous DMF (control). (Note: molarity values reflect final concentrations after addition of DBA to the 10 μl reaction.) Crosslinking was quenched after 30 s by the addition of an equal volume of 2× Laemmli buffer supplemented with 50 mM DTT and heated at 95 °C for 2 min prior to SDS-PAGE analysis using NuPAGE 3 to 8% Tris-Acetate Mini Protein Gels (Invitrogen).

### Proteolysis and mass spectrometry analysis to confirm the sites of DBA crosslinking

*Holo*-form RIFS M1-TEII was purified according to the above procedure, concentrated to ≈10 mg/ml, and incubated at 10 μM with 0.2 mM TCEP in 200 mM citric acid, 20 mM hepes, pH 7.3 (NaOH) for 15 min before adding 20 μM DBA or an equal volume of 50% aqueous DMF (control). (Note: molarity values reflect final concentrations after addition of DBA to the 40 μl reactions.) Crosslinking was quenched after 30 s by the addition of 4 mM β-mercaptoethanol (BME). Aliquots of the quenched reactions (5 μg) were diluted in 12 mM sodium lauroyl sarcosinate, 0.5% sodium deoxycholate, 50 mM triethylammonium bicarbonate, then reduced and alkylated using 10 mM TCEP and 40 mM chloroacetamide (20 min, 95 °C). For buffer exchange, SP3 SpeedBead Magnetic Carboxylate-Modified Particles (250 μg total, Cytiva; Cat# 65152105050250 and 4515210505250, mixed 1:1 ν/ν) were added. Ethanol was added (55% final concentration), and samples were gently agitated (15 min), followed by three washes with 80% ethanol. Proteins were eluted from the beads with 50 μl of 50 mM triethylammonium bicarbonate containing 0.25 μg of mass spectrometry-grade trypsin/Lys-C Mix (Promega, Cat# V50711) and digested overnight (37 °C, 16 h).

#### MS data collection for the DBA-crosslinked peptides

An aliquot (5 μl, 200 ng) of each sample was injected onto a PepMap Neo C18 HPLC column (Thermo Fisher Scientific, 150 μm × 150 mm, Cat# DNV150150PN) equilibrated in solvent A (0.1% formic acid) and separated using a Vanquish Neo UPLC system (Thermo Fisher Scientific) with an optimized 25-min gradient of solvent B (acetonitrile/water/formic acid, 80/20/0.1, ν/ν/ν) as follows (min/%B/flow rate μL/min): 0/5/2.45, 0.5/6/1.75, 12.6/32/1.75, 13.6/48/1.75, 13.7/99/2.45, 15/99/2.45 (Fig. S4). Eluted peptides were introduced into a nanospray ionization source coupled to an Orbitrap Astral mass spectrometer (Thermo Fisher Scientific) operated in data-dependent acquisition mode. Full MS scans were acquired in the Orbitrap (*m/z* 200–3000, automated gain control (AGC) target 300%, maximum injection time 5 ms, resolution 240,000 at *m/z* 200). MS2 scans were acquired in the Astral analyzer using a 1.5 *m/z* isolation window, 28% HCD collision energy, AGC target 70%, and a maximum injection time of 3 ms.

#### MS data collection for the DBA-reacted (uncrosslinked) peptides

An aliquot (5 μl, 200 ng) of each sample was injected onto a PepMap Neo C18 HPLC column (Thermo Fisher Scientific, 75 μm × 500 mm, Cat# DNV75500PN), equilibrated in solvent B (acetonitrile/water/formic acid, 80/20/0.1, ν/ν/ν), and eluted (100 nl/min) with an increasing concentration of solvent B (min/% B; 0/5, 40/32, 50/56, 52/100, 54/100, 56/2, 70/2) using an Easy nLC-1000 system (Thermo Fisher Scientific) (Figs. S5–S6). The effluent from the column was directed to a nanospray ionization source connected to a hybrid quadrupole-Orbitrap mass spectrometer (Q Exactive, Thermo Fisher Scientific) acquiring mass spectra in a data-dependent mode alternating between a full scan (*m/z* 350–1700, AGC target 3 × 106, 100 ms maximum injection time, FWHM resolution 70,000 at *m/z* 200) and up to 15 MS/MS scans (quadrupole isolation of charge states 2–7, isolation window 1.5 *m/z*) with previously optimized fragmentation conditions (normalized collision energy of 28, AGC target 1 × 105, 100 ms maximum injection time, FWHM resolution 17,500 at *m/z* 200).

#### MS data analysis and identification of modified peptides

Raw proteomic data were searched against databases containing the recombinant RifA protein sequence (NCBI: WP_013222547.1; Uniprot: O54666), the complete *E. coli* proteome, as well as common mass spectrometry contaminants using SEQUEST-HT in Proteome Discoverer (Version 3.3, Thermo Fisher Scientific), which provided measurements of relative abundance of the identified peptides (Figs. S4–S6). Dynamic modifications for the search included the following: oxidation (+15.995) on M, deamidation (+0.984) on N/Q, acetylation (+42.011) on K, phosphorylation (+79.966) on S, T, Y, methylation (+14.016) on K/R, 4′-phosphopantetheine (Ppant) addition (+340.086) on S, Ppant cofactor + carbamidomethylation (+397.107) on S, Ppant cofactor + DBA + hydroxylation (+412.107) on S, Ppant cofactor + DBA + TCEP (+645.164) on S, Ppant cofactor + DBA + BME (+472.110) on S, DBA + hydroxylation (+72.021) on C, DBA + TCEP (+305.242) on C, DBA + BME (+132.024) on C, and DBA + Ppant + DAGFDSLTAVELR (+1786.785) on C (*e.g.*, Fig. 2*C*). Decoy database searching was used to filter for high-confidence peptide identifications (FDR <1%). The detection of b and y fragment ions from tryptic peptides was used to confidently identify peptides belonging to specific proteins from the database within each sample. Chromatographic peak areas were utilized to assess the relative abundance of each peptide within each sample.

### Preparation of crosslinked RIFS M1-TEII for single-particle cryo-EM analysis

To isolate crosslinked M1-TEII for downstream single-particle cryo-EM analysis, multiple identical DBA crosslinking reactions were initiated, quenched, and pooled. This approach was necessary to avoid previously noted mixing effects at increased reaction volume that reduced crosslinking efficiency (15). A total of 20 × 55 μl reactions were carried out as above with slight adaptations. Specifically, *holo*-form M1-TEII was incubated at 12 μM concentration with 0.2 mM TCEP in 300 mM citric acid, 20 mM Hepes, pH 7.3 (NaOH) for 15 min before adding 24 μM DBA (total M1-TEII = 13.2 nmol). (Note: molarity values reflect final concentrations after addition of DBA to the 55 μl reaction.) Reactions were quenched after 30 s by the addition of 4 mM BME and buffer exchanged into SEC buffer using 7 kDa MWCO Zeba spin desalting columns (Thermo Fisher Scientific). Ten representative reactions were analyzed by SDS-PAGE (Fig. S7*C*). The crosslinked M1-TEII material was pooled and used immediately for complexation with F_ab_ 1B2 followed by SEC purification and cryo-EM analysis (Cryo-EM sample preparation and data collection).

### Isolation of crosslinked and uncrosslinked module + F_ab_ 1B2 complexes for cryo-EM analysis

RIFS modules (M1-DD and M1-TEII) and F_ab_ 1B2 were individually purified *via* SEC as above prior to repurification of the module–F_ab_ complexes. Module–F_ab_ complexes were prepared by adding 1.5 equivalents of 1B2 heterodimer per equivalent of PKS monomer, in accordance with the binding stoichiometry (21), and incubated on ice for 30 min before SEC purification of the complex (see Protein expression and purification and Fig. S7).

### Cryo-EM sample preparation and data collection

Crosslinked and uncrosslinked module–F_ab_ complexes were concentrated to ≈10 mg/ml using Amicon Ultra Centrifugal Filters (30 kDa MWCO) before adding 0.03% nonyl phenoxypolyethoxylethanol (NP-40), 0.8 mM NADPH, and 0.8 mM TCEP and applying 3 μl onto glow-discharged 300-mesh R 2/1 Quantifoil copper grids. The grids were glow-discharged for 30 s (10 s hold) at 15 mA with a PELCO easiGlow prior to sample addition, blotted for 4 s at 4 °C and 100% relative humidity, and vitrified in liquid ethane using a Vitrobot Mark IV (Thermo Fisher Scientific). The vitrified samples were imaged at 300 or 200 kV accelerating voltage with a Krios G3i or Glacios transmission cryo-electron microscope, respectively (Thermo Fisher Scientific). The Krios G3i was equipped with a K3 direct-electron detector (DED) and BioQuantum energy filter (Gatan), whereas the Glacios was equipped with a Falcon4 DED (Thermo Fisher Scientific) and no energy filter. The data were collected at nominal magnifications of 81,000 × (Krios G3i) or 130,000 × (Glacios), corresponding to a calibrated sampling of 1.1 Å/pixel or 0.73 Å/pixel, respectively. EPU software (Thermo Fisher Scientific) was used to record dose-fractionated movies in nongain normalized .tiff format with a total dose of 50 e^–^/Å^2^ and dose rates of 10.58 or 6.65 e^–^·pixel^−1^ s^−1^ on the Krios G3i or Glacios, respectively (Table S4).

### Single-particle Cryo-EM image processing, 3D reconstruction, and trajectory analysis

For details associated with cryo-EM data processing, see Figures S8 and S15. For all datasets, dose-fractionated movies were applied to motion correction, dose weighting, and contrast transfer function estimation in cryoSPARC v4.7.1 (41). Additional jobs performed in cryoSPARC v4.7.1 included particle picking, particle extraction, 2D classification, *ab initio* reconstruction, and homogenous refinement. Relion version 5.0 (42) was used for 3D classification by converting cryoSPARC particles in .cs format into .star format *via* the csparc2star.py script in pyem (https://zenodo.org/records/3576630). In each case, *ab initio* models generated from ≈10 to 20% of the total curated micrographs were used to create templates and perform reference-based particle picking from the entire sets of curated micrographs. C1 symmetry was specified for all 3D reconstructions and refinements. Finally, the particles of each class were imported to EMAN2 and subjected to Gaussian mixture model–based orientation refinement, as well as patch-by-patch refinement, improving the resolution of flexible domains (Figs. S11 and S18) (32). To characterize the continuous movement of the DH° domain, a mask was created to cover the domain, and the Gaussian mixture model–based heterogeneity analysis in EMAN2 was used to extract the motion trajectory. A series of volumes, each reconstructed from 10,000 particles, was generated along the first eigenvector in the conformational space to visualize movement of the DH° domain and associated CP occupancy (Supporting Movie).

### Model building and refinement

Atomic models for individual domains (DH°, KR, and CP) and didomains (KS-AT) of RIFS M1-DD and M1-TEII were generated in AlphaFold 3 and fit as rigid bodies into their corresponding cryo-EM maps using ChimeraX (43, 44). Linker regions with supporting map density were built manually in Coot (45), and the entire map/model combinations were automatically refined in Phenix (46) using *Real-space Refinement* (47). The 4′-phosphopantetheine (Ppant) cofactor was modeled by substituting the Ser residue that becomes 4′-phosphopantetheinylated with a “4HH” residue in Coot. The DBA crosslink could not be observed in any of the cryo-EM maps associated with crosslinked M1-TEII and was therefore not modeled.

### Preparation of fluorescein-labeled carrier protein probe

A 50 mM stock solution of FITC was freshly prepared in anhydrous DMSO before the reaction was initiated by adding 2.5 mM FITC (10 equiv.) to a solution of 250 μM *holo*-form CP from RIFS LM (CPL) in 0.3 M NaH_2_PO_4_ pH 8 (NaOH) (30 min, room temperature ‘RT’). (Note: molarity values reflect final concentrations after addition of FITC to the 130 μl reaction.) The reaction was terminated *via* buffer exchange into 0.1 M NaH_2_PO_4_ pH 7.3 (NaOH) using 7 kDa MWCO Zeba spin desalting columns (two cycles). Absorbance measurements of the eluant containing fluorescein-labeled *holo*-CPL (CPL∗) were performed using a NanoDrop OneC (pedestal) to estimate the CPL:fluorescein molar ratio. Roughly 40% of the CPL∗ was labeled with fluorescein based on the optical parameters of FITC (λ_max_ = 495 nm, ε = 74,000 M^−1^ cm^−1^) and CPL (λ_max_ = 280 nm, ε = 5500 M^−1^ cm^−1^), and the assumption that 30% of the absorbance at 280 nm stemmed from FITC (Fig. S21*A*).

### DBA crosslinking of CPL∗ with *holo*-form RIFS M1-DD, M1-KS2°AT2, or M1-TEII

Ten equivalents of CPL∗ (50 μM) were added to independent 20 μl reactions containing 5 μM *holo*-form RIFS M1-DD, M1-KS2°AT2 or M1-TEII, in 0.1 mM TCEP, 300 mM citric acid, 20 mM Hepes, pH 7.3 (NaOH) and incubated (15 min, RT). DBA (15 μM) or an equal volume of 50% aqueous DMF (control) was added to initiate crosslinking reactions (30 s, RT) followed by the addition of an equal volume of 2× Laemmli buffer supplemented with 50 mM DTT, heating at 95 °C for 2 min, and SDS-PAGE analysis using NuPAGE 3 to 8% Tris-Acetate Mini Protein Gels (Invitrogen). Similar reactions containing 0.1 M NaH_2_PO_4_ pH 7.3 (NaOH) buffer in place of CPL∗ were analyzed in parallel following the same protocol (Fig. S21, *B* and *C*).

### Single-turnover kinetic analysis of LM-M1–catalyzed diketide formation by LC-MS/MS-MRM

(Note: molarity values reflect final concentrations after addition of all components.)

#### Assays without pre-incubation

A 160 μl enzymatic reaction was initiated by combining 5 μM *holo*-form LM-M1-DD, LM-M1-TEII°, or LM-M1-DD(C802A) with 50 mM sodium phosphate (pH 7.3, NaOH; supplemented with 1/18 equiv. Hepes), 100 mM sodium citrate (pH 7.3, NaOH; supplemented with 1/18 equiv. Hepes), 10 mM TCEP, 15 mM MgCl_2_, 10% glycerol, 1 mM sodium benzoate, 5 mM ATP, 1 mM NADPH, and 1 mM (2*R,S*)-MeMal-CoA (racemate). After 1, 5, 15, 60, and 120 min at RT, 20 μl aliquots were subjected to alkaline hydrolysis by adding 5 M KOH to a final concentration of 350 mM and incubating at 65 °C for 20 min (Fig. 5*A*). Formic acid was then added to a final concentration of 2.5% (ν/ν), the hydrolysates were centrifuged (12,000*g*, 10 min), and a 10 μl aliquot from each sample was injected onto a Poroshell 120 EC-C18 column (4.6 × 100 mm, 2.7 μm, Agilent Technologies) using a 1290 Infinity II HPLC system (Agilent Technologies) and eluted at a flow rate of 600 μl/min with solvent A (aqueous 20 mM ammonium acetate) and solvent B (acetonitrile) using a 9.5-min gradient (min/% B: 0/10, 3/100, 3.5/100, 4/10, 9.5/10). The column effluent was directed to an OptiFlow Turbo V Electrospray ionization source connected to a quadrupole mass spectrometer (AB Sciex LLC, Triple Quad 5500^+^) acquiring data in the targeted multiple reaction monitoring negative ion mode. The hydrolyzed diketide (**d2′**) product precursor ion (*m/z* 179) was fragmented at a specific collision energy (−17 eV) to produce characteristic product ions (*m/z* 73 and *m/z* 117) at a specific LC retention time (2.6 min). To ensure specificity and accurate quantification in the complex biological samples, both transitions (*m/z* 179 → 117 and 73) were monitored, where the more intense transition (*m/z* 179 → 73) was used for quantitation, and the less intense transition (*m/z* 179 → 117) was used for qualification (Fig. S23). Chromatographic peaks were extracted and integrated using Analyst (AB Sciex LLC) software. Technical replicates of each enzymatic reaction were performed in triplicate.

#### Assays with pre-incubation

A 140 μl enzymatic reaction was initiated by combining 5 μM *holo*-form LM-M1-DD or LM-M1-TEII° with 50 mM sodium phosphate (pH 7.3, NaOH; supplemented with 1/18 equiv. Hepes), 100 mM sodium citrate (pH 7.3, NaOH; supplemented with 1/18 equiv. Hepes), 10 mM TCEP, 15 mM MgCl_2_, 10% glycerol, 1 mM sodium benzoate, and 5 mM ATP. The contents were incubated (150 min, RT) to allow LM-catalyzed benzoylation to reach a maximum extent (pre-incubation). Afterward, the reaction was supplemented with 1 mM NADPH and 1 mM MeMal-CoA (racemate) to initiate diketide (**d2**) formation (Fig. 5*A*). After 0.25, 0.5, 1, 5, 15, and 30 min at RT, 20 μl reaction aliquots were treated as above, and the supernatants were subjected to LC-MS/MS-MRM analysis as above. Technical replicates of each enzymatic reaction were performed in triplicate.

#### Diketide formation rates of site-directed LM-M1 bimodule variants

A 140 μl enzymatic reaction was initiated by combining 5 μM *holo*-form LM-M1-DD, LM-M1-DD-D1537R, LM-M1-DD-D1677R, or LM-M1-DD-D1537R/D1677R with 50 mM sodium phosphate (pH 7.3, NaOH; supplemented with 1/18 equiv. Hepes), 100 mM sodium citrate (pH 7.3, NaOH; supplemented with 1/18 equiv. Hepes), 10 mM TCEP, 15 mM MgCl_2_, 10% glycerol, 1 mM sodium benzoate, 5 mM ATP, 1 mM NADPH, 1 mM MeMal-CoA (racemate). After 1, 5, 15, 60, and 120 min at RT, 20 μl reaction aliquots were treated as above, and the supernatants were subjected to LC-MS/MS-MRM as above, except under slightly modified LC gradient conditions. That is, the hydrolyzed diketides (**d2′** and **keto-d2′**) were eluted at a flow rate of 500 μl/min with solvent A (water with 0.1% formic acid) and solvent B (60% methanol, 40% acetonitrile) using a 10.5-min gradient (min/% B: 0/15, 6/100, 8/100, 8.1/15, 10.5/15). Otherwise, the samples were analyzed and the data processed as above. Technical replicates of each enzymatic reaction were performed in triplicate.

#### Diketide formation in the presence or absence of NADPH

A 140 μl enzymatic reaction was initiated by combining 5 μM *holo*-LM-M1-DD with 50 mM sodium phosphate (pH 7.3, NaOH; supplemented with 1/18 equiv. Hepes), 100 mM sodium citrate (pH 7.3, NaOH; supplemented with 1/18 equiv. Hepes), 10 mM TCEP, 15 mM MgCl_2_, 10% glycerol, 1 mM sodium benzoate, 5 mM ATP, and 1 mM MeMal-CoA (racemate) in the presence or absence of 1 mM NADPH. After 1, 5, 30, 60,120 and 180 min at RT, a 20 μl reaction aliquot was treated as above, and the supernatants were subjected to LC-MS/MS-MRM analysis under the modified LC gradient conditions described above (Figs. S26 and S27). Again, technical replicates of each enzymatic reaction were performed in triplicate.

### Quantification of LM-M1–bound diketide by LC-MS/MS-MRM

(Note: molarity values reflect final concentrations after addition of all components.) Authentic **d2′** was prepared at 45 nM, 450 nM, and 4500 nM in the presence of 3.5 μM *holo*-LM-M1-DD or *holo*-LM-M1-TEII° in 50 mM sodium phosphate (pH 7.3, NaOH; supplemented with 1/18 equiv. Hepes), 100 mM sodium citrate (pH 7.3, NaOH; supplemented with 1/18 equiv. Hepes), 10 mM TCEP, 15 mM MgCl_2_, and 10% glycerol (reaction buffer). Enzymatic reactions (30 μl) were initiated separately by incubating 3.5 μM *holo*-LM-M1-DD or *holo*-LM-M1-TEII° in the above reaction buffer (15 min, RT) prior to addition of 1 mM sodium benzoate, 5 mM ATP, 1 mM NADPH, and 1 mM MeMal-CoA (racemate). Reactions were incubated (3 h, RT) and terminated *via* buffer exchange into reaction buffer using 7 kDa MWCO Zeba spin desalting columns (Thermo Fisher Scientific). (This protocol was originally designed to enable simultaneous detection of protein-bound benzoyl and diketide (**d2**) thioester groups. However, the buffer exchange step was later determined to be unnecessary for diketide quantification). The protein recovery following buffer exchange was determined by protein absorbance based on the calculated molecular weights and extinction coefficients. Each sample, including the above standards and enzymatic reactions, was subjected to alkaline hydrolysis by adding 5 M KOH to a final concentration of 350 mM and incubating at 65 °C for 20 min (Fig. 5*A*). Upon completion of hydrolysis, formic acid was added (2.5% final concentration, ν/ν), and the hydrolysates were centrifuged (12,000*g*, 10 min). The supernatants were subjected to LC-MS/MS-MRM analysis as above with a slightly modified 9.5-min gradient (min/% B: 0/10, 5/100, 6/100, 6.1/10, 9.5/10). Data corresponding to the **d2′** standards were used to generate linear standard curves (Fig. S23) from which **d2′** concentrations were calculated in the above enzymatic reactions. Note: (1) the reported concentrations of **d2′** in Figure S23*B* reflect their final concentrations after buffer exchange and sample workup and (2), to ensure reproducibility, all protein concentrations in this section were determined by protein absorbance based on the calculated molecular weights and extinction coefficients. While the concentrations determined by Bradford assay corresponded closely with those determined by absorbance (≤10% difference), the greater precision of absorbance-based quantification was deemed important for reproducibility across replicates. Technical replicates of each **d2′** standard and enzymatic reaction were performed in triplicate.

### Synthesis of diketide 2-methyl-3-oxo-3-phenylpropanote (keto-d2′)

The chemical synthesis of **keto-d2′** was conducted following the previously reported protocol (48). Ethyl 2-methyl-3-oxo-3-phenylpropanoate (50 mg, 0.242 mmol) was dissolved in two equivalents of 3% KOH (0.91 ml, 0.485 mmol) and (i) saponified by stirring at RT for 36 h, (ii) acidified to pH 2 with 2 M HCl, and (iii) extracted three times with methyl *tert*-butyl ether. The pooled organic phases were dried and the residue dissolved in acetonitrile for HPLC purification. The separation was conducted on an Agilent 1100 HPLC system with a Phenonenex Kinetex C18 column (150 × 10.0 mm, 5 μm) equilibrated in 70% eluant A (H_2_O/TFA, 100/1) and 30% eluant B (CH_3_CN/TFA, 100/0.1) and eluted over 18-min at 2 ml/min with an increasing concentration of eluant B (min/%B: 0/30, 15/70, 15.1/30, 18/30). The compound eluted at 8.0 min and was collected and dried *in vacuo* for structural analysis by LC-MS, LC-MS/MS, and NMR spectroscopy (Figs. S25 and S27). This species was confirmed to be the desired compound **keto-d2′** by the MS and MS/MS spectra and following proton signals (Oxford NMR AS400 MHz): 12.71 ppm (br s, COOH), 8.00 ppm (dd, 7.4 Hz, 1.2 Hz, H5/H5′), 7.69 ppm (tt, 7.4 Hz, 1.2 Hz, H7), 7.55 ppm (t, 7.4 Hz, H6/H6′), 4.59 ppm (q, 7.0 Hz, H2), and 1.30 ppm (d, 7.0 Hz, 8-CH_3_). Other unlabeled signals in the spectrum are derived from DMSO-*d*_6_ (2.50 ppm), CH_3_CN (2.07 ppm), and H_2_O (3.35 ppm).

## Data availability

Associated atomic coordinates and cryo-EM maps have been deposited to the Protein Data Bank under accession codes 9PAT (*transacylation-mode*), 9PAV (*elongation-mode*), and 9PC6 (CL-M1-TEII-1B2) and to the Electron Microscopy Data Bank under accession codes EMD-71445, EMD-71446, and EMD-71497, respectively. Raw cryo-EM data have been deposited to the Electron Microscopy Public Image Archive under accession codes EMPIAR-13347 and EMPIAR-13348. Any other data associated with this manuscript can be made available upon request.

## Supporting information

This article contains supporting information (8, 10, 11, 13, 15, 21, 22, 26, 31, 32, 33, 34, 40, 41, 42, 47, 49, 50, 51, 52, 53, 54, 55, 56, 57, 59).

## Conflicts of interest

The authors declare that they have no conflicts of interest with the contents of this article.
