# Supplementary material for: C-terminal dimerization motifs control asynchronous chain elongation during modular polyketide biosynthesis[image]
Source: J Biol Chem. 2026 May 6;302(6):113119. doi: 10.1016/j.jbc.2026.113119 (PMC13254586; doi:10.1016/j.jbc.2026.113119)
Supplement: Supporting Information [file mmc1.docx]

Supporting Information for:

**C-terminal dimerization motifs control asynchronous chain elongation during modular polyketide biosynthesis**

Chengli Liu^1^, Ryan C. West^1^, Muyuan Chen^2^, Whitaker Cohn^1^, George Wang^1^, Selena Kim^1^, Aryan M. Mandot^1^, Kym F. Faull^1^, and Dillon P. Cogan^1*^

^1^ Department of Pharmacology and Pharmaceutical Sciences, University of Southern California, Los Angeles, CA 90089, USA

^2^ Division of CryoEM and Bioimaging, Stanford Synchrotron Radiation Lightsource, SLAC National Accelerator Laboratory, Stanford University, Menlo Park, CA 94025, USA

*Corresponding author: [dcogan@usc.edu](mailto:dcogan@usc.edu)

**Table of Contents**

Supporting Figures s2–s31

Figure S1: Catalytic cycle of rifamycin synthetase (RIFS) starter unit loading and elongation s2

Figure S2: Crosslinking RIFS module 1 (M1) variants with 1,3-dibromoacetone (DBA) (pt. 1) s3

Figure S3: Crosslinking RIFS M1 variants with DBA (pt. 2) s4

Figure S4: Tryptic digestion and mass spectrometry analysis of crosslinked RIFS M1 (pt. 1) s5–s6

Figure S5: Tryptic digestion and mass spectrometry analysis of crosslinked RIFS M1 (pt. 2) s7

Figure S6: Tryptic digestion and mass spectrometry analysis of crosslinked RIFS M1 (pt. 3) s8

Figure S7: Size-exclusion chromatography analysis of RIFS M1 variants ± crosslinking s9

Figure S8: Single-particle cryo-EM data collection and processing of RIFS M1+F_ab_. s10

Figure S9: Cryo-EM map validation of the *transacylation mode* of RIFS M1+F_ab_ s11

Figure S10: Cryo-EM model validation of the *transacylation mode* of RIFS M1+F_ab_ s12

Figure S11: Supplemental cryo-EM map of the *transacylation mode* of RIFS M1+F_ab_ s13

Figure S12: Cryo-EM map validation of the *elongation mode* of RIFS M1+F_ab_ s14

Figure S13: Cryo-EM model validation of the *elongation mode* of RIFS M1+F_ab_ s15

Figure S14: Close-up view of carrier protein interactions with enzymatic domains of RIFS M1 s16

Figure S15: Single-particle cryo-EM data collection and processing of crosslinked RIFS M1+F_ab_ s17

Figure S16: Cryo-EM map validation of crosslinked RIFS M1+F_ab_ s18

Figure S17: Cryo-EM model validation of crosslinked RIFS M1+F_ab_ s19

Figure S18: Supplemental cryo-EM map of crosslinked RIFS M1+F_ab_ s20

Figure S19: WebLogo and ConSurf analysis of the ketoreductase-carrier protein linker of RIFS M1 s21

Figure S20: Kinetic and mutational analysis of RIFS loading and elongation bimodule (LM-M1) s22

Figure S21: DBA crosslinking of RIFS M1 fused with its native C-terminal KS-AT didomain s23

Figure S22: DBA crosslinking of the nocardiosis-associated polyketide synthase module 1 s24

Figure S23: LC-MS/MS-MRM quantification of hydrolyzed diketide analog (**d2′**) s25

Figure S24: Effect of substrate pre-incubation on LM-M1 kinetics s26

Figure S25: ^1^H-NMR spectrum of hydrolyzed unreduced diketide analog (**keto-d2′**) s27

Figure S26: Kinetics of reduced (**d2**) vs. unreduced (**keto-d2**) diketide formation ± NADPH s28–s29

Figure S27: MS/MS spectrum of **keto-d2′** s30

Figure S28: Uncropped gel images used in this study s31

Supporting Tables s32–s35

Table S1: Primary proteins used in this study s32

Table S2: Protein domain/linker color key s33

Table S3: DNA plasmids used in this study s34

Table S4: Cryo-EM data collection, refinement, and validation statistics s35

Protein Sequences s36–s46

Caption for Supporting Movie s47

Supporting References s48–s49

**Supporting Figures**


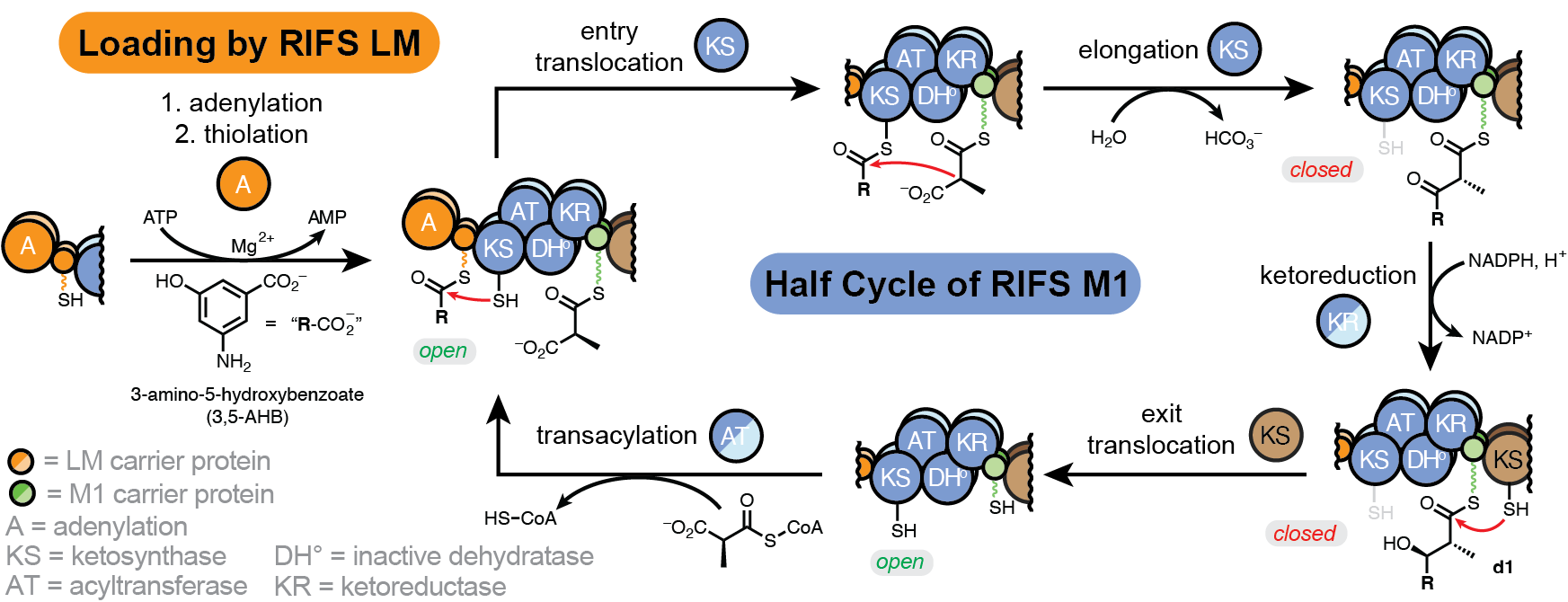


**Fig. S1.** Catalytic cycle of starter unit loading and elongation catalyzed by the rifamycin synthetase (RIFS) loading module (LM) + module 1 (M1). The adenylation (A) domain catalyzes two-step 3-amino-5-hydroxybenzoylation of its downstream carrier protein (CP) domain, in orange. The first step involves ATP-dependent adenylation of 3-amino-5-hydroxybenzoate (3,5-AHB) into 3-amino-5-hydroxybenzoyl-AMP. The second step involves transfer of the activated 3-amino-5-hydroxybenzoyl group onto the 4′-phosphopantetheine (Ppant) cofactor of the LM’s CP domain (CPL, squiggly lines = Ppant). The ketosynthase (KS) domain of the downstream PKS module (M1) then catalyzes transfer of the 3-amino-5-hydroxybenzoyl group onto its own catalytic Cys residue (entry translocation). (It was previously proposed that this transfer occurs after acyltransferase (AT)-catalyzed transacylation of 2*S*-methylmalonyl (MeMal) from MeMal-CoA onto the Ppant cofactor of M1’s CP domain, in light green(1).) Attachment of both acyl groups onto M1 primes KS-catalyzed decarboxylative Claisen condensation between the MeMal nucleophile and KS-bound electrophilic thioester, resulting in a β-ketoacyl-CP thioester product (elongation). The ketoreductase (KR) domain catalyzes stereospecific ketoreduction to the (2*R*,3*S*) diastereomer of the diketide (**d1**), which is then received by the downstream KS from RIFS module 2 (M2) (exit translocation). The dehydratase domain of M1 is inactive (denoted DH°), presumably due to presence of a Gly in place of the conserved catalytic His (position 1521 in RifA, WP_013222547.1)(2). This illustration depicts the activities of a single catalytic subunit defined as the complete set of enzymes required for one catalytic half-cycle. However, it does not necessarily reflect accurately which of the two catalytic subunits are employed from one half-cycle to the next, nor does it reflect the possibility that activities on both subunits happen concurrently. Note: in accordance with previous evidence, heavy and light shading of the two subunits was used to illustrate *intra*-molecular entry/exit translocation and *inter-*molecular elongation(3, 4). The KS Cys is shown in black and gray to indicate active (open) and inactive (closed) states, respectively, in accordance with the prior described ‘turnstile’ mechanism(5). That is, after KS-catalyzed elongation, the KS becomes transiently inactivated until exit translocation of the polyketide intermediate reverts the KS into an active state.

**
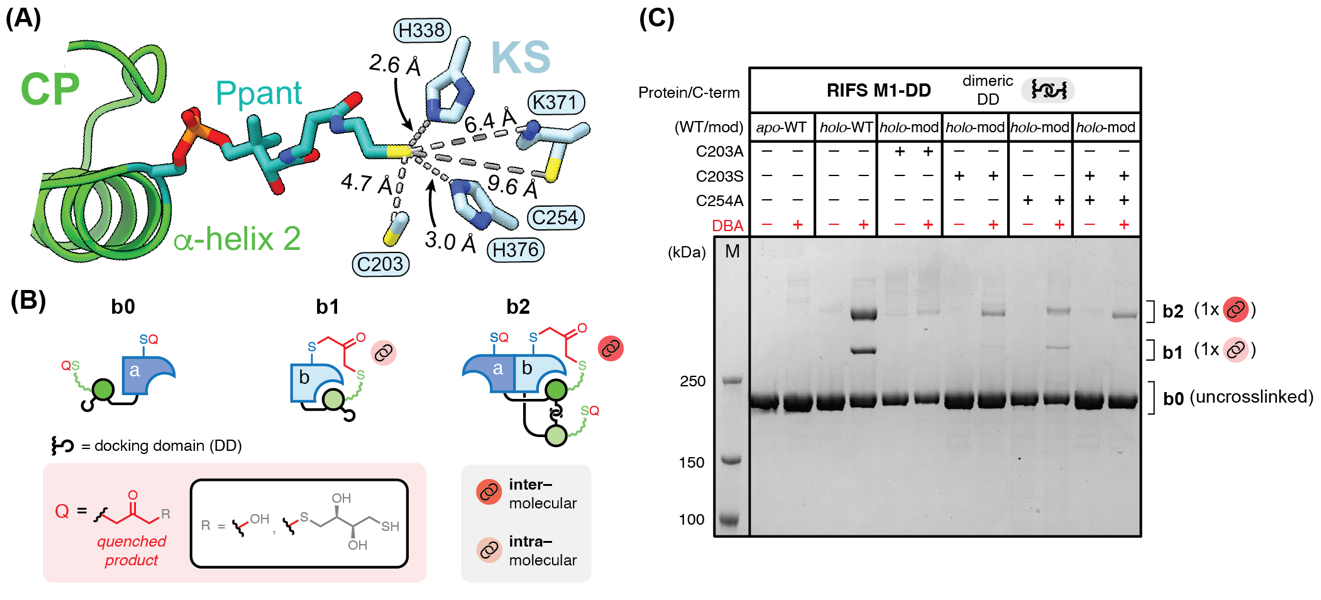
**

**Fig. S2.** Site-selectivity of DBA crosslinking was investigated through mutational analysis of RIFS M1. **(A)** The structure of RIFS M1 in the *elongation mode* (PDB 9PAV) was used to guide the selection of nucleophilic residues for genetic mutation and analysis of their contributions to DBA crosslinking. **(B)** Cartoon depictions of the three major products following addition of DBA to RIFS M1 before denaturation by SDS-PAGE. **(C)** Wild-type (WT) or genetically modified (mod) RIFS M1 in *holo*- or *apo*-forms were subjected to DBA crosslinking (or addition of DMF as a control) in the presence of 0.1 mM TCEP, 20 mM HEPES, and 300 mM citrate (pH 7.3), followed by reducing SDS-PAGE analysis (n=1, technical replicate). Only trace amounts of non-specific products, relative to starting materials, were observed in the *apo*-WT M1 reactions, indicating that the Ppant thiol group of the CP domain was essential for formation of the major crosslinked products observed in *holo*-WT M1: i.e., bands 1–2 (**b1**–**b2**). The uncrosslinked protein is labeled as band 0 (**b0**). Genetic mutation of the catalytic Cys residue of the KS domain to Ala (C203A) or Ser (C203S) significantly reduced crosslinking suggesting that **b1** and **b2** principally consisted of Ppant-Cys203 crosslinks. A similar reduction was observed when Cys254, which is positioned ~10 Å away from the Ppant in the cryo-EM structure, was converted to Ala (C254A). Combining two of these site-directed modifications (C203S/C254A) indicated that other nucleophilic residues besides Cys203 and Cys254 (e.g., His338, Lys371, or His376) are likely to partake in crosslinking with Ppant. The near complete loss of **b1** in the crosslinked C203A, C203S, and C203S/C254A proteins implied that this product is mainly dependent on Cys203. While these experiments established that other nucleophiles besides Cys203 can crosslink with Ppant, only the Ppant-Cys203 major crosslink is depicted in the graphical illustration for simplicity.


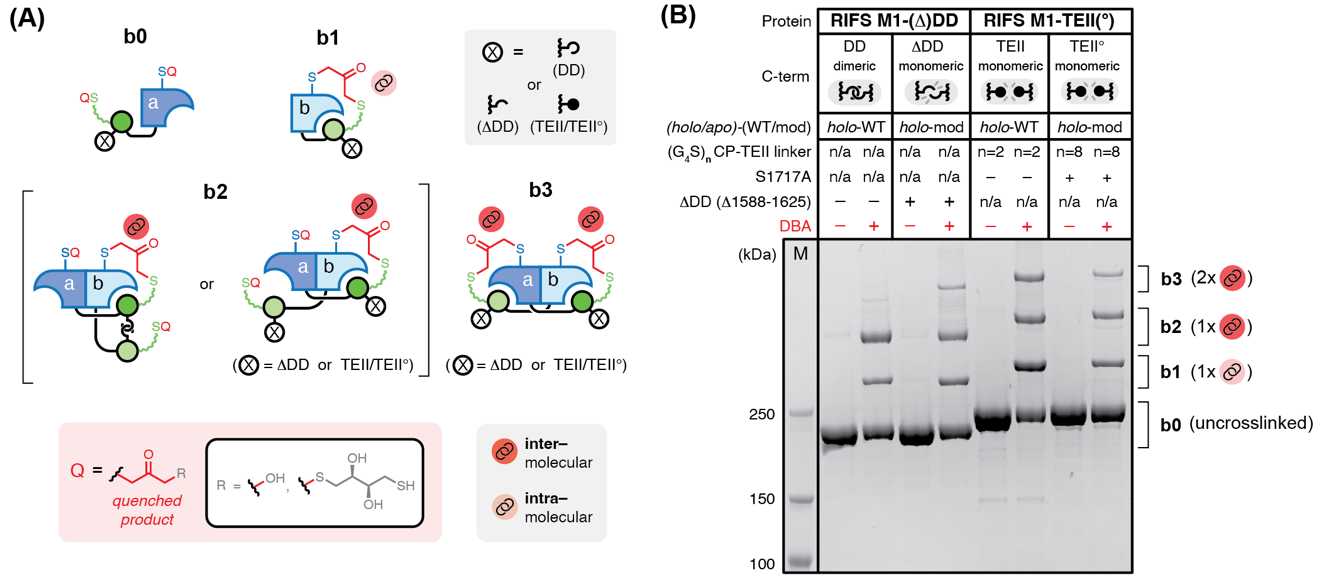


**Fig. S3.** DBA crosslinking analysis of RIFS M1-DD, M1-ΔDD, M1-TEII, and M1-TEII° (the ‘°’ implies introduction of a TEII-inactivating genetic mutation (S1717A) and a longer (G_4_S)_8_ M1-TEII linker). **(A)** Cartoon depictions of the four major products following addition of DBA to these proteins before denaturation by SDS-PAGE. **(B)** DBA or DMF (control) were added to the above proteins in the presence of 0.1 mM TCEP, 20 mM HEPES, and 300 mM citrate (pH 7.3) and analyzed by reducing SDS-PAGE (n=1, technical replicate). The appearance of a band distribution in M1-ΔDD like that of M1-TEII pointed to an inhibitory role for DD in the formation of band 3 (**b3**). Similar crosslinked products (**b1**–**b3**) observed in M1-TEII and M1-TEII° indicated that the linker between the CP and TEII domains (i.e., S(G_4_S)_2_ vs. S(G_4_S)_8_, respectively) was not essential for crosslinking, nor was the active site Ser1717 of TEII.

**
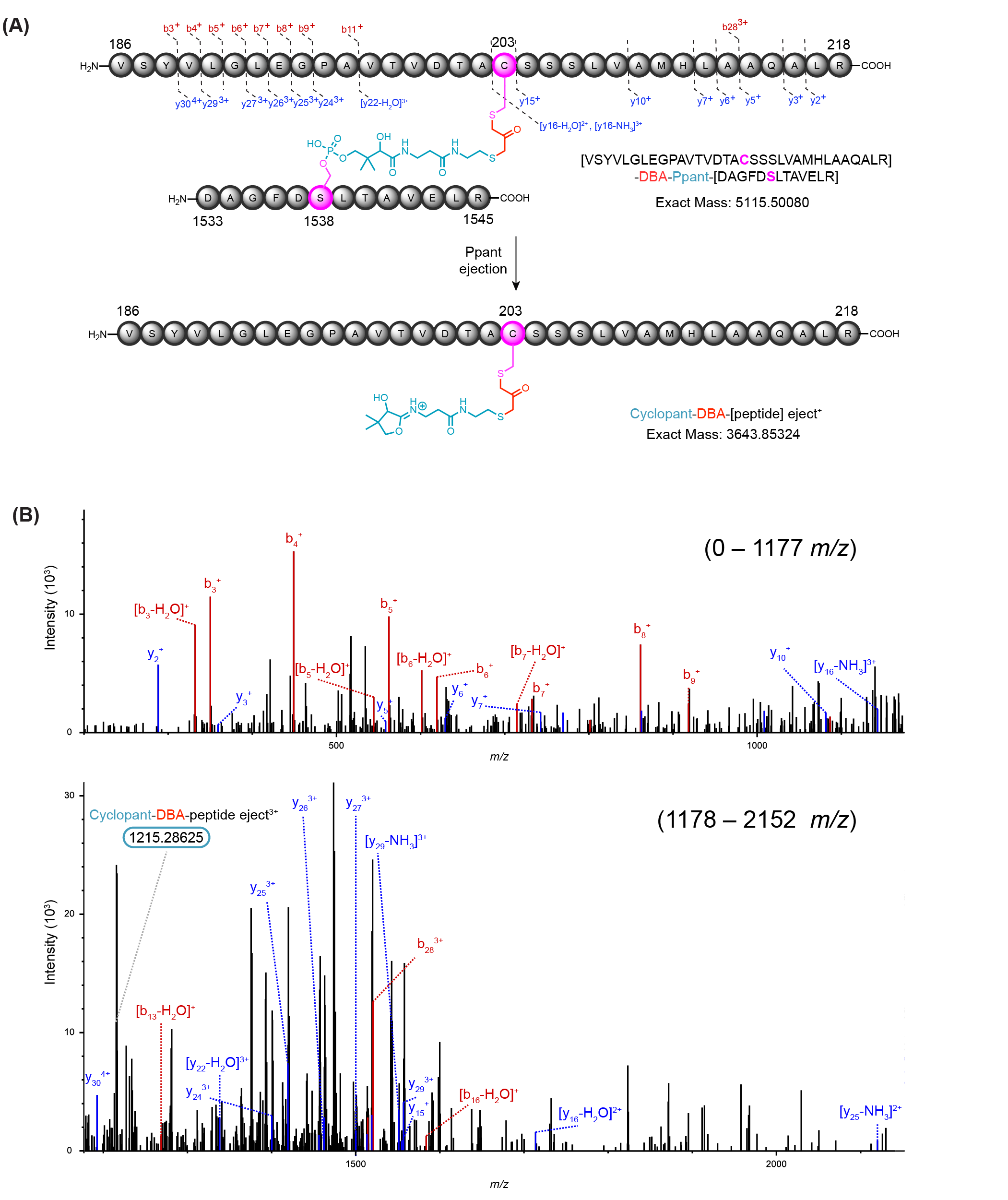
**

**Fig. S4 (Part 1 of 2).** *(See next page for Panel C + Legend)*

**
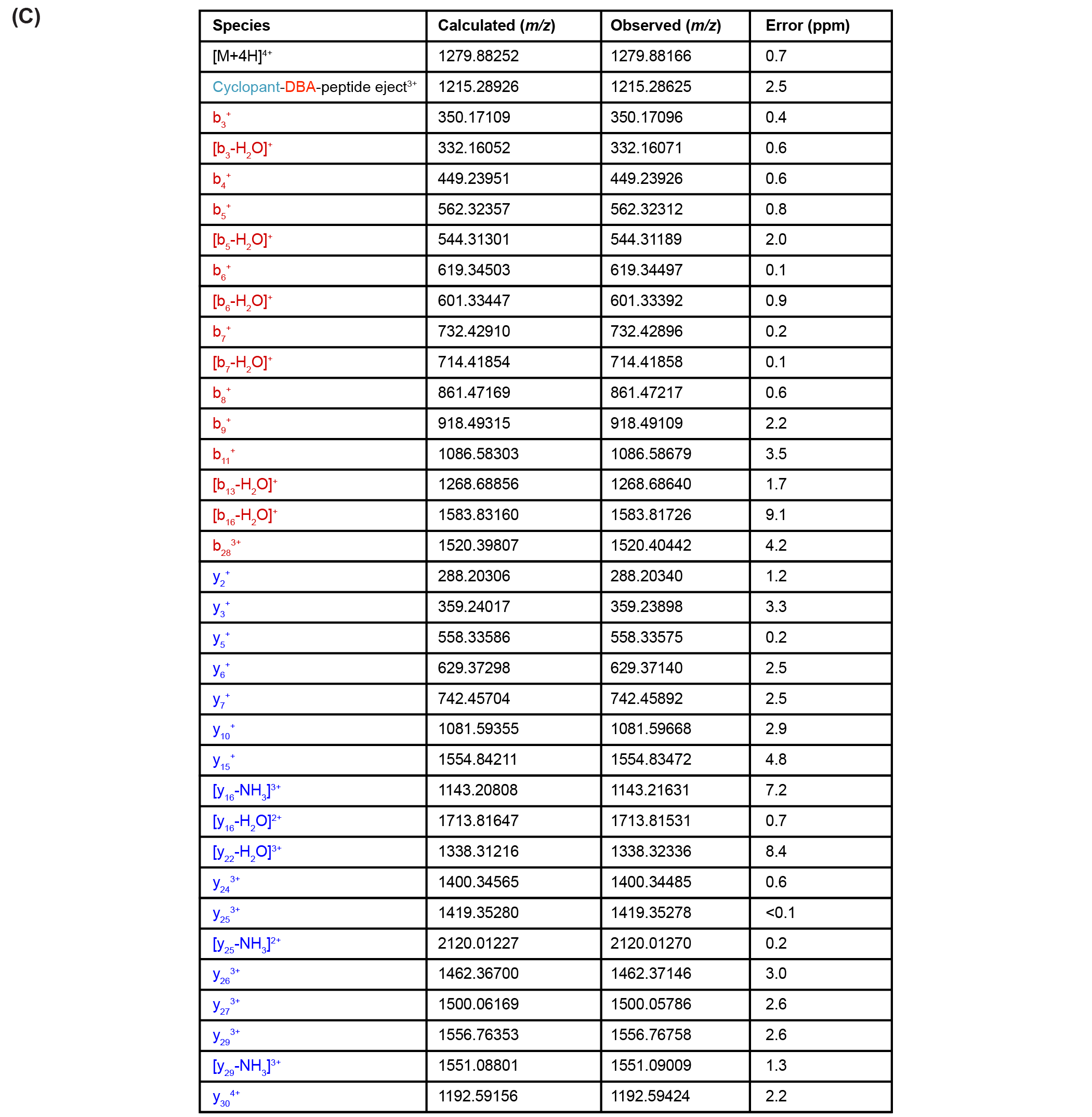
**

**Fig. S4 (Part 2 of 2).** Verification of crosslinking sites (Cys203 and Ser-Ppant1538, labeled in magenta) by tandem mass spectrometry analysis of DBA-treated and trypsinized, *holo*-form M1-TEII. **(A)** Cartoon schematic and **(B)** LC-MS/MS fragmentation pattern of the crosslinked, tryptic peptides and their detected b and y fragment ions. A spontaneous “Ppant ejection” reaction leading to formation of a cyclic pantetheine (cyclopant) residue along with loss of a peptide containing phosphoserine at position 1538 was predicted to occur inside the mass spectrometer, as reported earlier(6). A molecular ion that matched the exact mass of the cyclopant-linked species (cyclopant-DBA-[peptide] eject^+^) within 2.5 ppm mass error was detected in the triply charged state. **(C)** Table of observed molecular ions (*m/z*) and their associated mass errors (ppm). Ser1538 becomes 4′-phosphopantetheinylated by Sfp in *E. coli* BAP1 (the Ppant modification is shown in cyan)(7). The residue numbering follows the sequence of RIFS M1-TEII (see **Protein Sequences**).


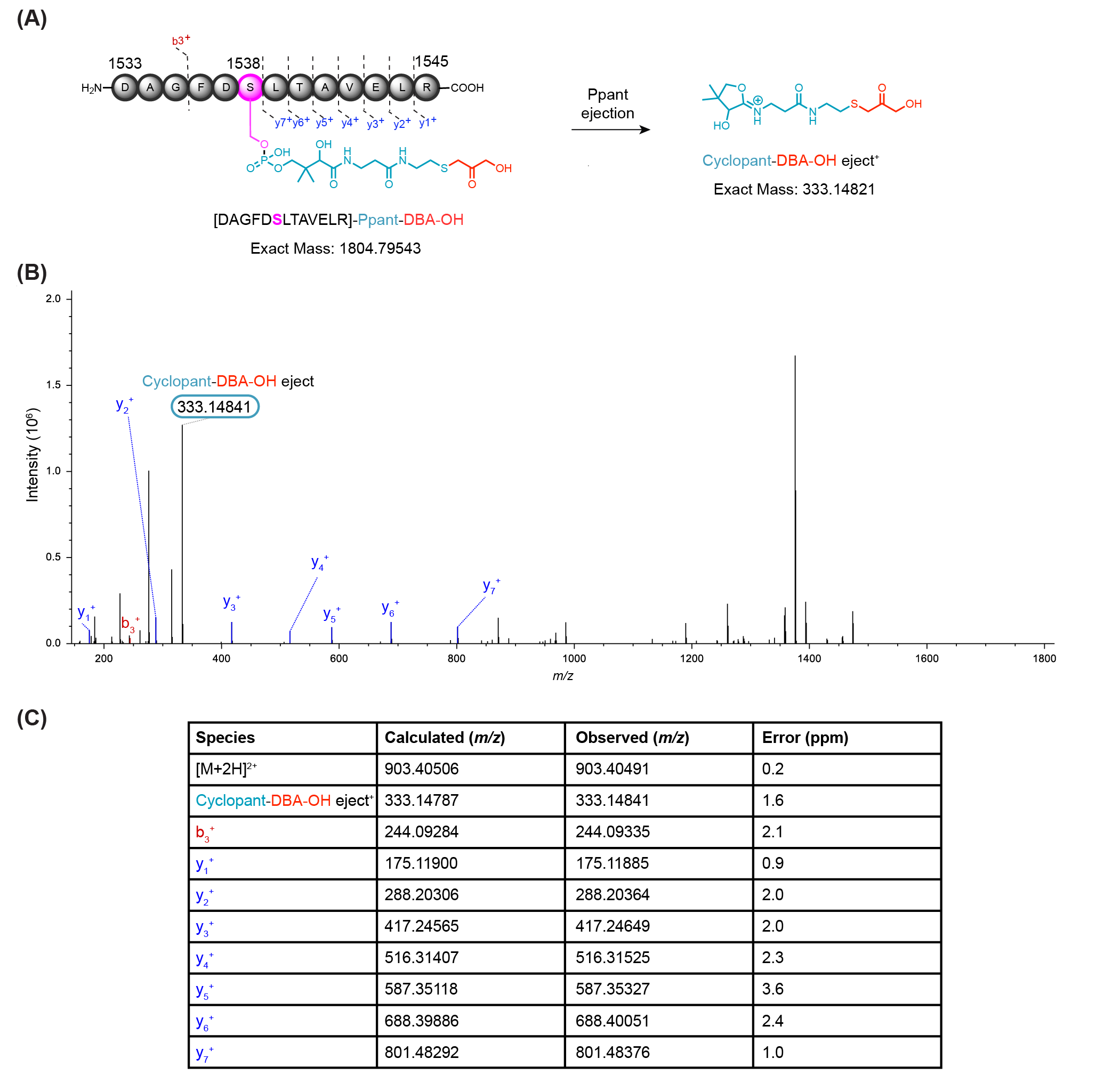


**Fig. S5.** Verification of a DBA reactive site (Ser-Ppant1538, labeled in magenta) by tandem mass spectrometry analysis of DBA-treated and trypsinized, *holo*-form M1-TEII. **(A)** Cartoon schematic and **(B)** LC-MS/MS fragmentation pattern of a DBA-reacted, tryptic peptide and its detected b and y fragment ions, which supported the existence of a covalent adduct located at Ser-Ppant1538 resulting from a two-step reaction with DBA and water (Fig. 2A,C). A spontaneous “Ppant ejection” reaction leading to formation of a cyclic pantetheine (cyclopant) residue along with loss of a peptide containing phosphoserine at position 1538 was predicted to occur inside the mass spectrometer, as reported earlier(6). A molecular ion that matched the exact mass of the cyclopant-linked species (cyclopant-DBA-OH eject^+^) within 1.6 ppm mass error was detected in the singly charged state. **(C)** Table of observed molecular ions (*m/z*) and their associated mass errors (ppm). Ser1538 becomes 4′-phosphopantetheinylated by Sfp in *E. coli* BAP1 (the Ppant modification is shown in cyan)(7). The residue numbering follows the sequence of RIFS M1-TEII (see **Protein Sequences**).

**
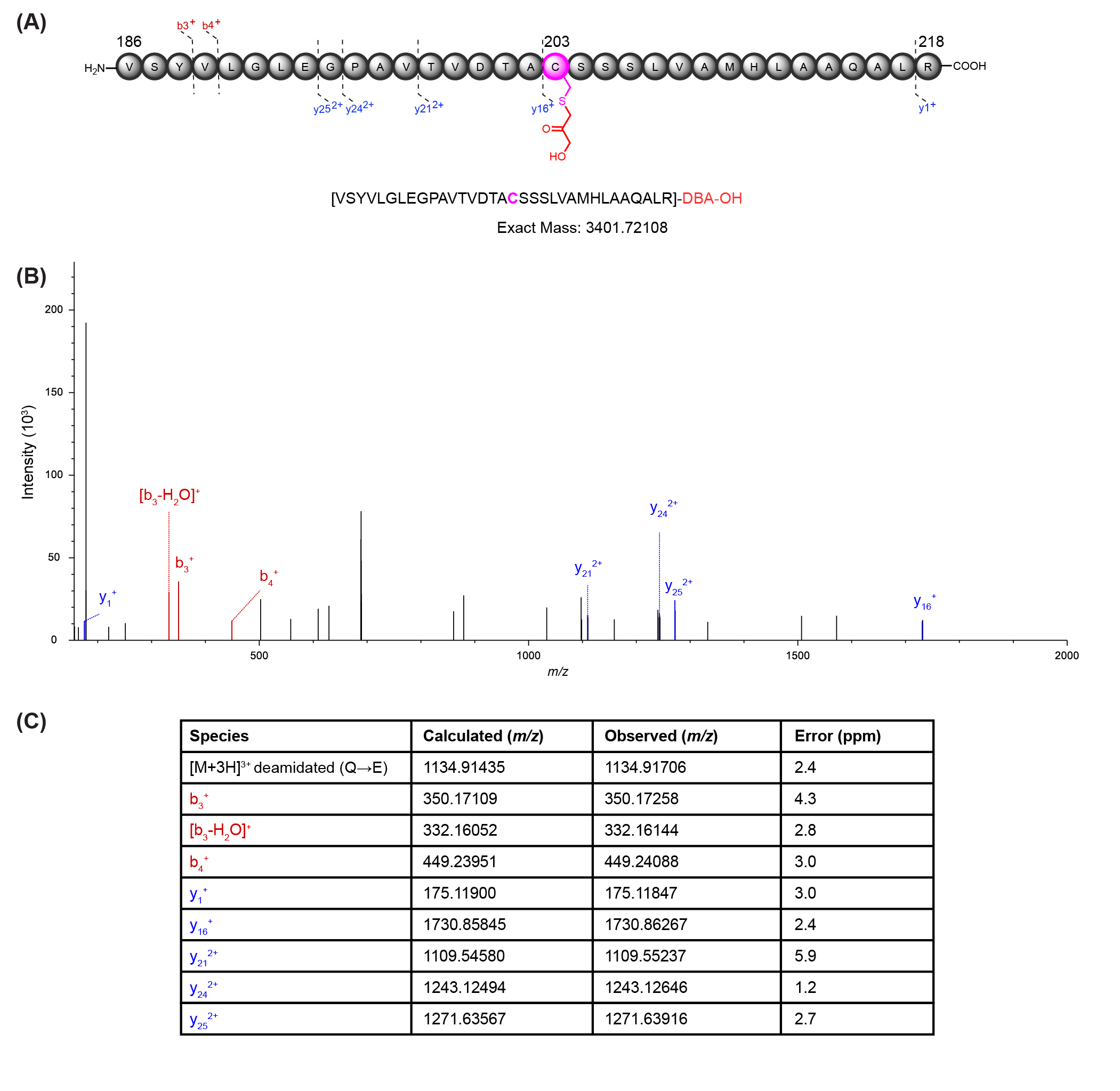
**

**Fig. S6.** Verification of a DBA reactive site (Cys203, labeled in magenta) by tandem mass spectrometry analysis of DBA-treated and trypsinized, *holo*-form M1-TEII. **(A)** Cartoon schematic and **(B)** LC-MS/MS fragmentation pattern of a DBA-reacted, tryptic peptide and its detected b and y fragment ions, which supported the existence of a covalent adduct located at Cys203 resulting from a two-step reaction with DBA and water (Fig. 2A,C). **(C)** Table of observed molecular ions (*m/z*) and their associated mass errors (ppm). Cys203 corresponds to the conserved catalytic residue of the KS domain. The residue numbering follows the sequence of RIFS M1-TEII (see **Protein Sequences**).

**
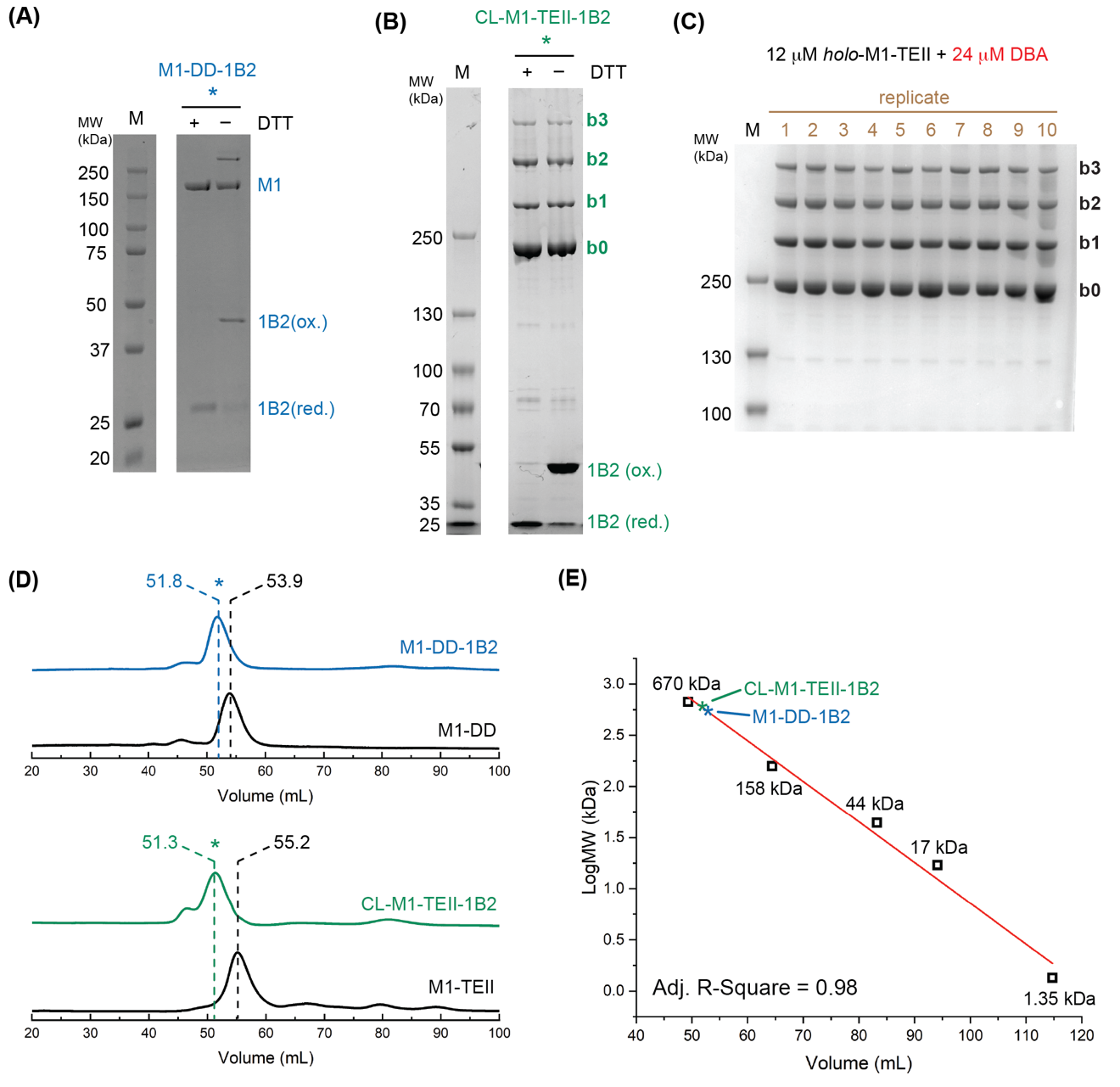
**

**Fig. S7.** SDS-PAGE and size-exclusion chromatography (SEC) analysis of each cryo-EM sample used in this study. SDS-PAGE analyses of the **(A)** M1-DD-1B2 and **(B)** CL-M1-TEII-1B2 complexes under reducing and non-reducing conditions (± 25 mM DTT). Electrophoresis in panel A was performed with a 4–20% Mini-PROTEAN® TGX™ Gel, whereas electrophoresis in panel B was performed with a 3–8% NuPAGE™ Tris-Acetate Mini Protein Gel. **(C)** 10 independent (technical) replicate crosslinking reactions featuring 12 μM *holo*-M1-TEII, 0.2 mM TCEP, 300 mM citric acid, 20 mM HEPES, pH 7.3 (NaOH), and 24 μM DBA were analyzed by SDS-PAGE. **(D)** SEC chromatograms of RIFS M1-DD or M1-TEII alone (black) and in complex with F_ab_ 1B2 (blue or green, respectively). Note: unlike M1-DD, M1-TEII was crosslinked prior to addition of 1B2 (see **Preparation of Crosslinked RIFS M1-TEII for Single-particle Cryo-EM Analysis)**. **(E)** A semi-log standard curve generated by similar SEC analysis of protein standards (Bio-Rad #1511901).

**
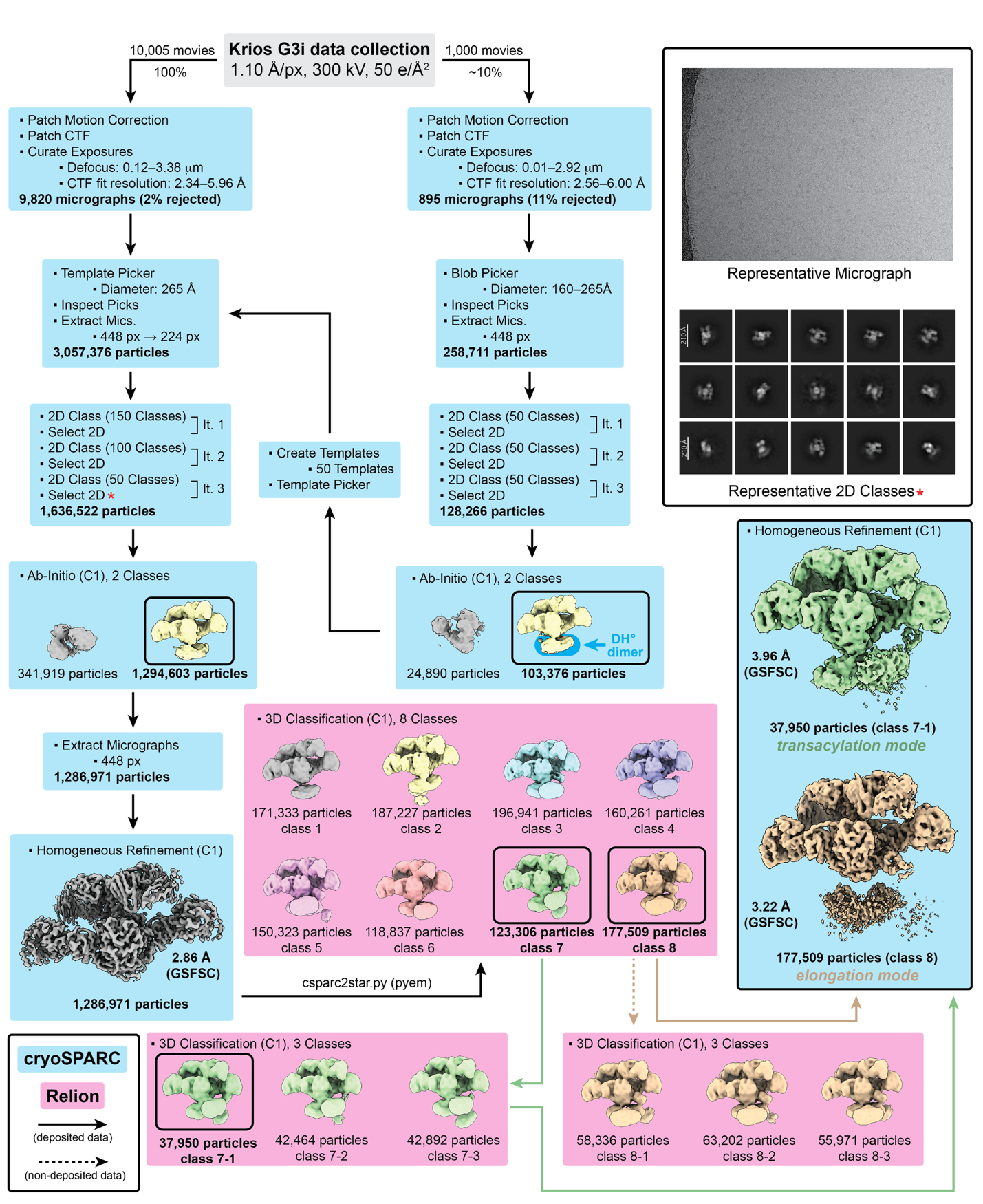
Fig. S8.** Workflow of single-particle cryo-EM data collection and processing of *uncrosslinked* M1-DD-1B2 (for sample details, see Fig. S7). A combination of *in silico* tools in Relion version 5.0(8) (pink) and cryoSPARC v4.7.1(9) (blue) were implemented to obtain the final cryo-EM maps (boxed).


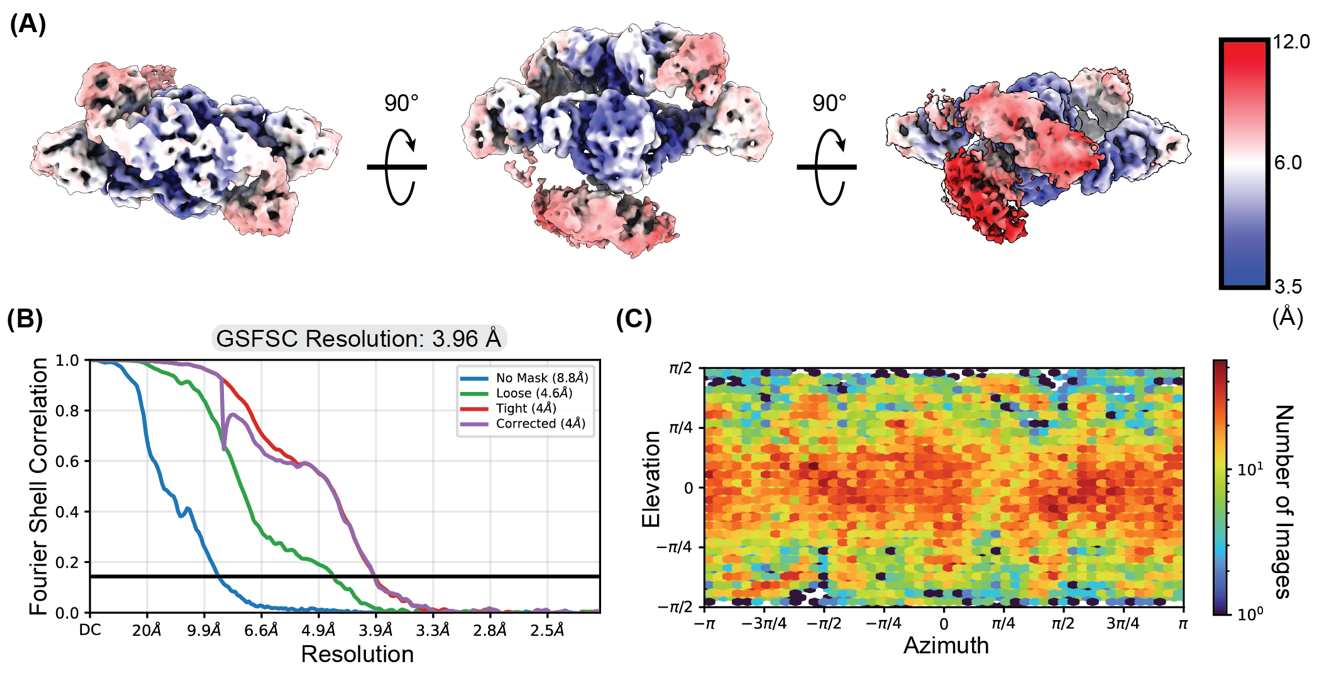


**Fig. S9.** Cryo-EM map validation of the *transacylation-mode* structure of M1-DD in complex with F_ab_ 1B2 (M1-DD-1B2; Fig. S8). CryoSPARC v4.7.1(9) was used to generate **(A)** a local resolution map, **(B)** Fourier shell correlation (FSC) curves, based on two independently refined half maps (GSFSC resolution = gold-standard FSC resolution, defined as the spatial frequency where FSC = 0.143)(10), and **(C)** an Euler angle distribution plot showing the number of images for each particle orientation.


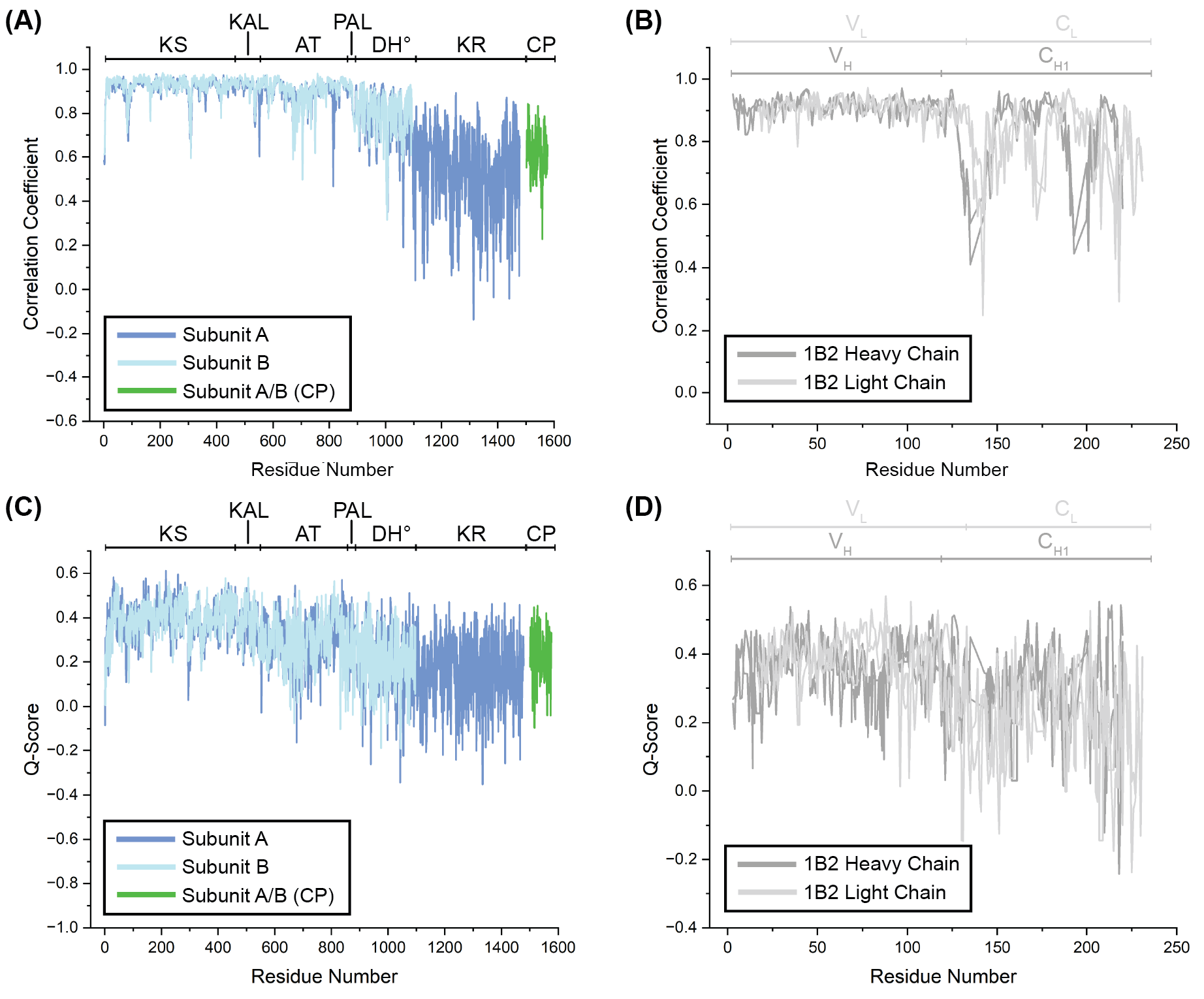


**Fig. S10.** Cryo-EM model validation of the *transacylation-mode* structure of M1-DD in complex with F_ab_ 1B2 (M1-DD-1B2; PDB 9PAT). Per-residue cryo-EM map and model correlation coefficients (CCs) determined by Phenix real-space refinement(11) are plotted for each subunit of **(A)** M1-DD and **(B)** F_ab_ 1B2. Likewise, per-residue Q-score assessments of atom resolvability within the cryo-EM map(12) are plotted for each subunit of **(C)** M1-DD and **(D)** F_ab_ 1B2. (KAL = KS-AT linker; PAL = post-AT linker; V_L_ = variable domain of the light chain; C_L_ = constant domain of the light chain; V_H_ = variable domain of the heavy chain; C_H1_ = first constant domain of the heavy chain.) AlphaFold 3 modeling and reference to structural homologs in the PDB guided initial model building in regions of the model with CC ~0.8 or less (i.e., PDB 4LN9 for DH°, PDB 3SLK for KR, and PDB 6C9U for C_L_ and C_H1_)(13).


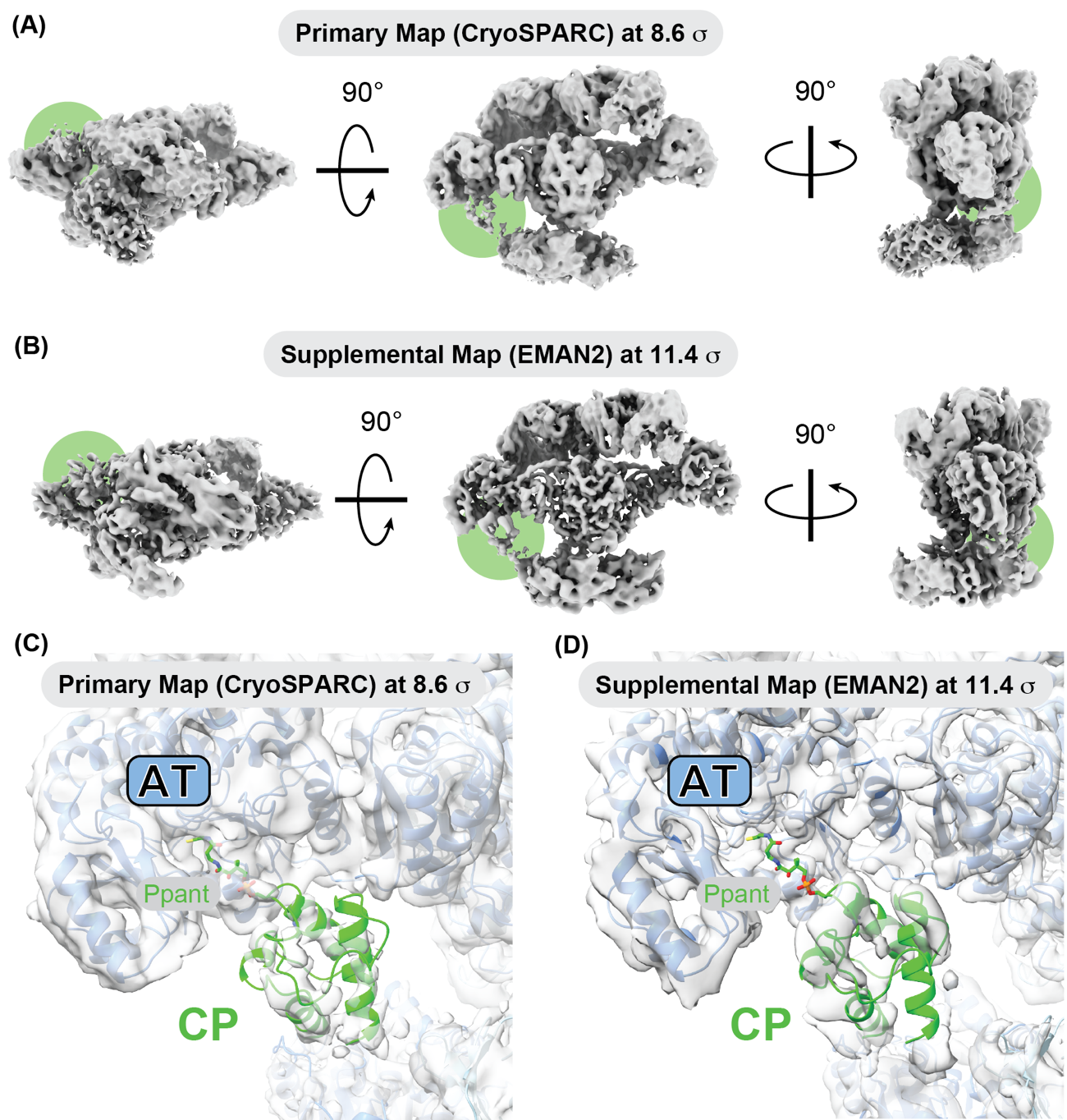


**Fig. S11.** Three different orientations of the **(A)** primary and **(B)** supplemental cryo-EM maps associated with the *transacylation-mode* structure (Figs. S8–S10) contoured at 8.6 σ and 11.4 σ, respectively. Green circles highlight regions of CP domain density. **(C–D)** Close-up views of the CP-bound AT active site cleft for the **(C)** primary and **(D)** supplemental maps highlight stronger signal for the CP domain in the supplemental map at higher stringency (sigma). Sigma values were calculated from the entire cryo-EM map volumes by the equation: σ = (threshold – mean) / standard deviation.


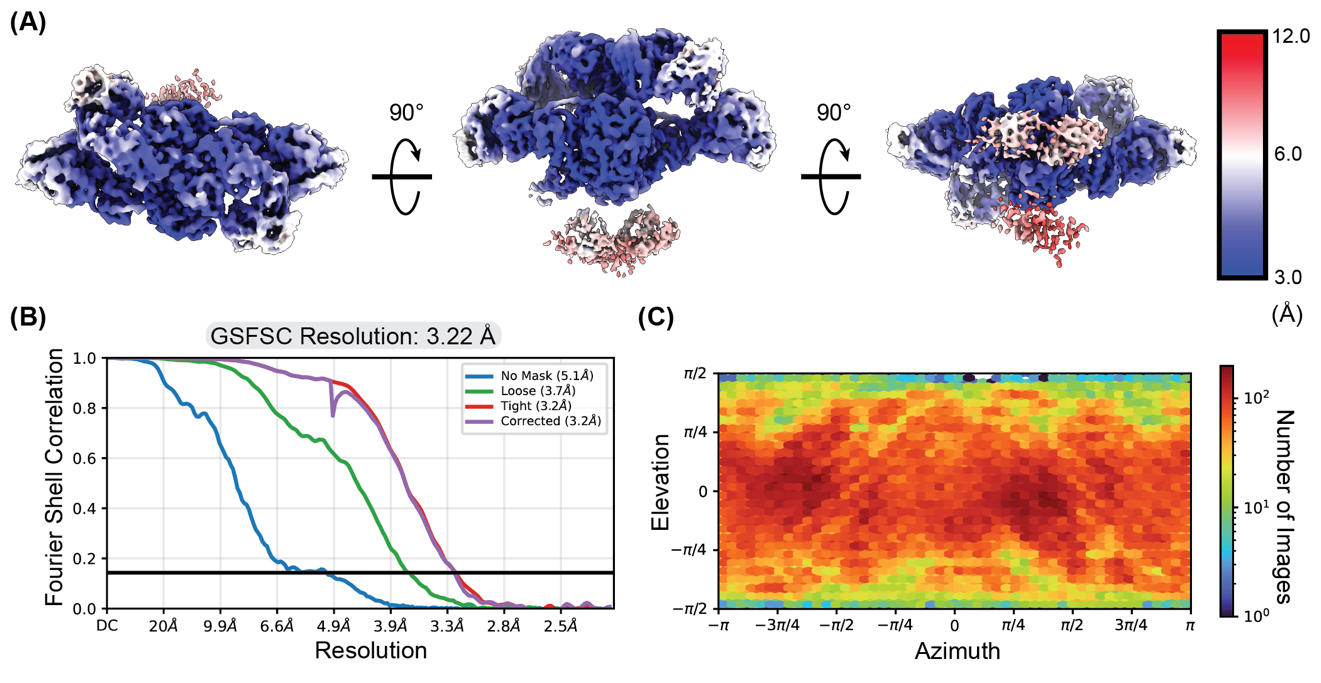


**Fig. S12.** Cryo-EM map validation of the *elongation-mode* structure of M1-DD in complex with F_ab_ 1B2 (M1-DD-1B2; Fig. S8). CryoSPARC v4.7.1(9) was used to generate **(A)** a local resolution map, **(B)** Fourier shell correlation (FSC) curves, based on two independently refined half maps (GSFSC resolution = gold-standard FSC resolution, defined as the spatial frequency where FSC = 0.143)(10), and **(C)** an Euler angle distribution plot showing the number of images for each particle orientation.


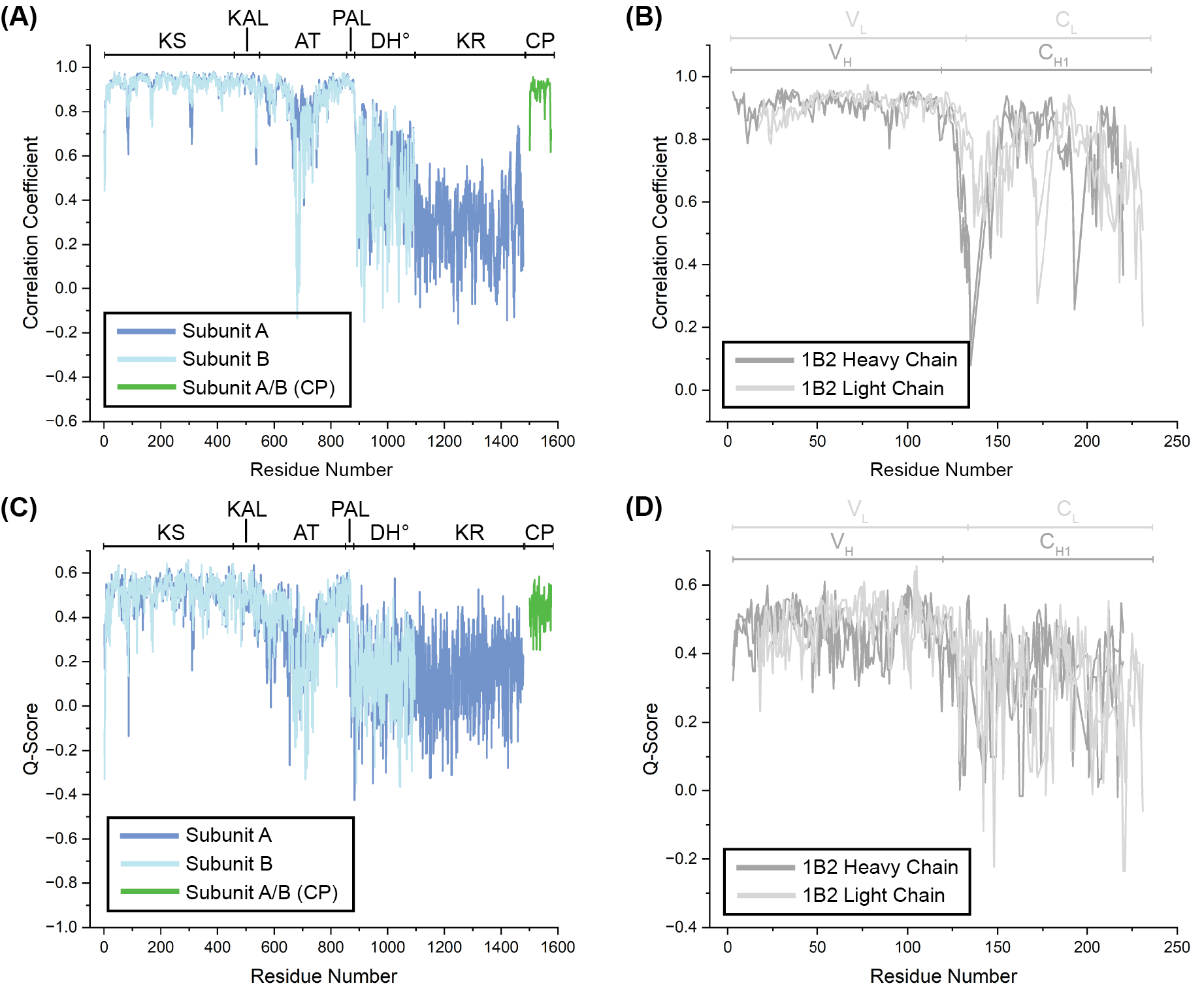


**Fig. S13.** Cryo-EM model validation of the *elongation-mode* structure of M1-DD in complex with F_ab_ 1B2 (M1-DD-1B2; PDB 9PAV). Per-residue cryo-EM map and model correlation coefficients (CCs) determined by Phenix real-space refinement(11) are plotted for each subunit of **(A)** M1-DD and **(B)** F_ab_ 1B2. Likewise, per-residue Q-score assessments of atom resolvability within the cryo-EM map(12) are plotted for each subunit of **(C)** M1-DD and **(D)** F_ab_ 1B2. (KAL = KS-AT linker; PAL = post-AT linker; V_L_ = variable domain of the light chain; C_L_ = constant domain of the light chain; V_H_ = variable domain of the heavy chain; C_H1_ = first constant domain of the heavy chain.) AlphaFold 3 modeling and reference to structural homologs in the PDB guided initial model building in regions of the model with CC ~0.8 or less (i.e., PDB 4LN9 for DH°, PDB 3SLK for KR, and PDB 6C9U for C_L_ and C_H1_)(13).


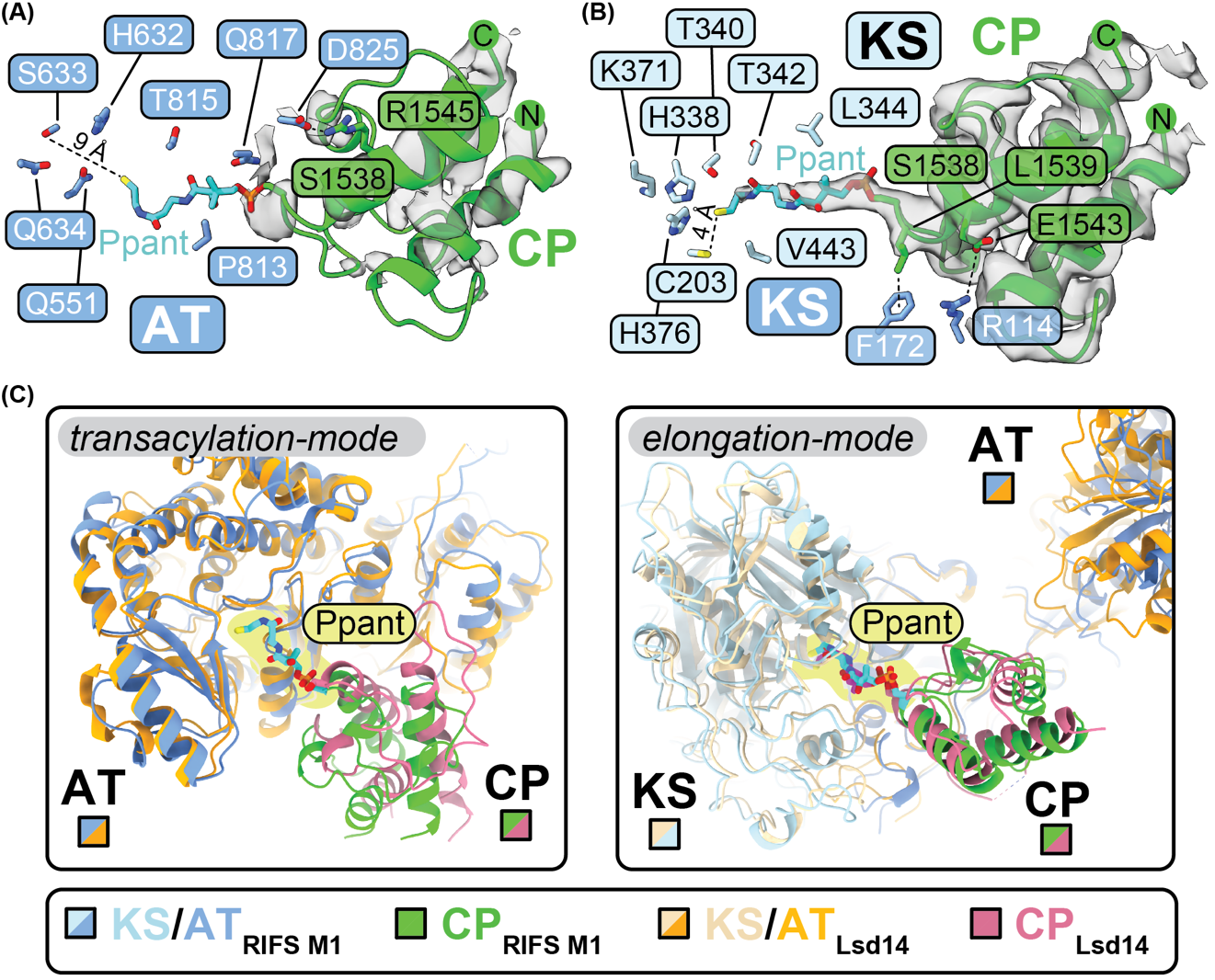


**Fig. S14.** Modeled interactions between the 4′-phosphopantetheine (Ppant)-attached CP domain and its **(A)** AT or **(B)** KS domain partners in the *transacylation-mode* (9PAT) and *elongation-mode* (9PAV) structures of RIFS M1, respectively (Figs. S8–S13). Focused refinement employing a spherical mask around the CP binding site yielded a supplemental cryo-EM map with increased signal in this region in the *transacylation-mode* structure (Fig. S11). Side chain coordinates were approximated by AlphaFold 3, given the insufficient local resolution for experimental map-based modeling (Figs. S9–S13)(13). Note: these cryo-EM maps were generated from uncrosslinked material. **(C)** Structural superposition of models in panels A and B with their structural homologs identified in FoldSeek(14). In each superposition, only the KS-AT didomain and CP domain coordinates were used. The *transacylation-mode* coordinates were superposed with those from PDB 7S6B (2.35 Å resolution, solved by X-ray diffraction) which features a similar binding interaction between the AT and *apo*-form CP domains from module 7 of the lasalocid A synthase (Lsd14)—root mean square deviation (RMSD) = 6.1 Å over 864 Cα atoms. Likewise, the *elongation-mode* coordinates were superposed with those from PDB 7S6C (3.1 Å GSFSC resolution, solved by single-particle cryo-EM) which features a similar binding interaction between the KS and *holo*-form CP domains from Lsd14—RMSD = 3.9 Å over 867 Cα atoms. The Ppant cofactor is displayed as cyan (RIFS M1) or magenta (Lsd14) sticks and highlighted in yellow.


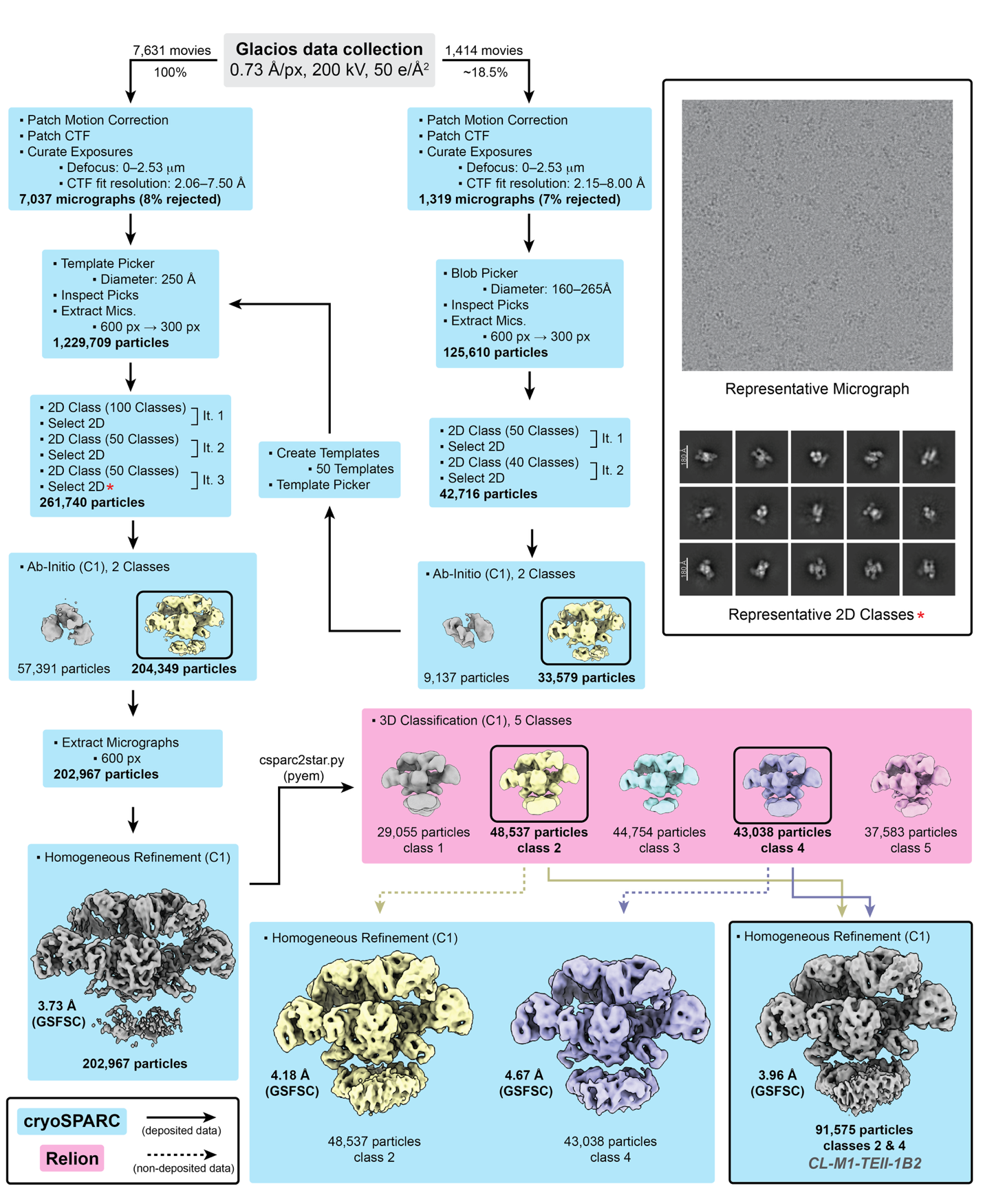


**Fig. S15.** Workflow of single-particle cryo-EM data collection and processing of CL-M1-TEII-1B2 (for sample details, see Fig. S7). A combination of *in silico* tools in Relion version 5.0(8) (pink) and cryoSPARC v4.7.1(9) (blue) were implemented to obtain the final cryo-EM map (boxed). Note that ‘class 3’ under 3D Classification contains density for only one CP domain, as expected for **b1** and **b2** (Figs. 2B,C and S7).

**
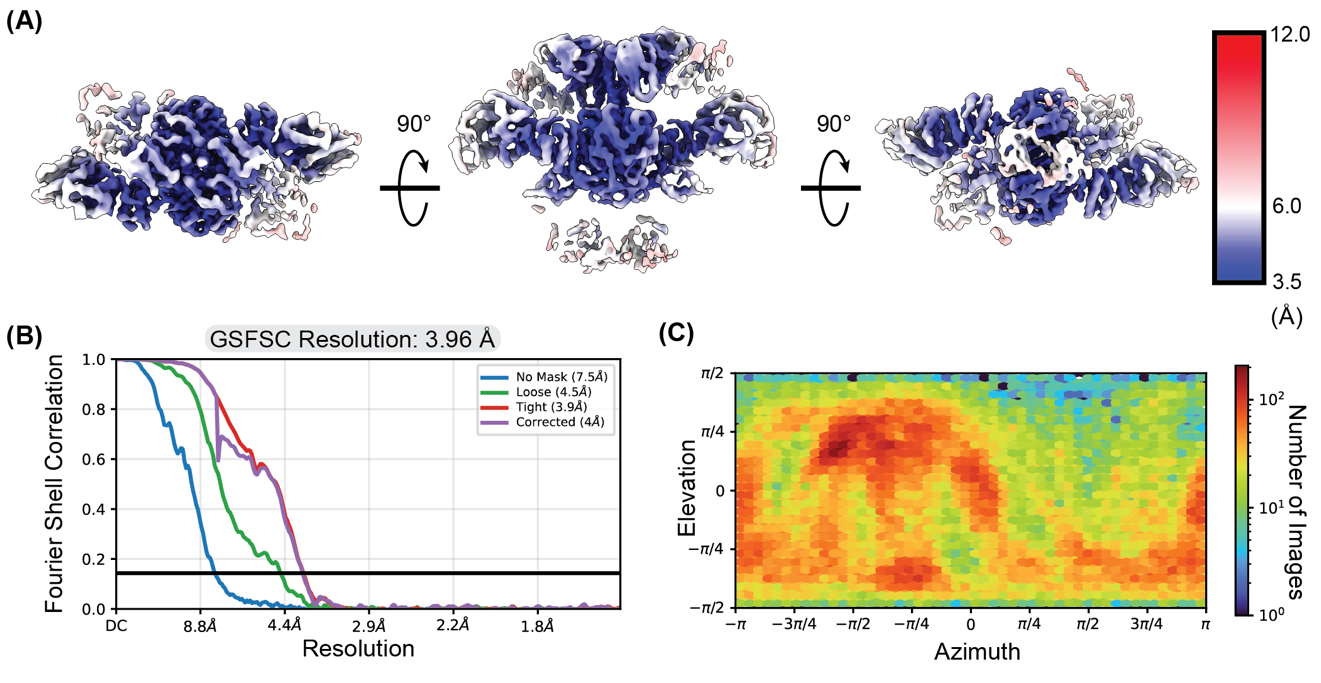
**

**Fig. S16.** Cryo-EM map validation of crosslinked M1-TEII in complex with F_ab_ 1B2 (CL-M1-TEII-1B2; Fig. S15). CryoSPARC v4.7.1(9) was used to generate **(A)** a local resolution map, **(B)** Fourier shell correlation (FSC) curves, based on two independently refined half maps (GSFSC resolution = gold-standard FSC resolution, defined as the spatial frequency where FSC = 0.143)(10), and **(C)** an Euler angle distribution plot showing the number of images for each particle orientation.


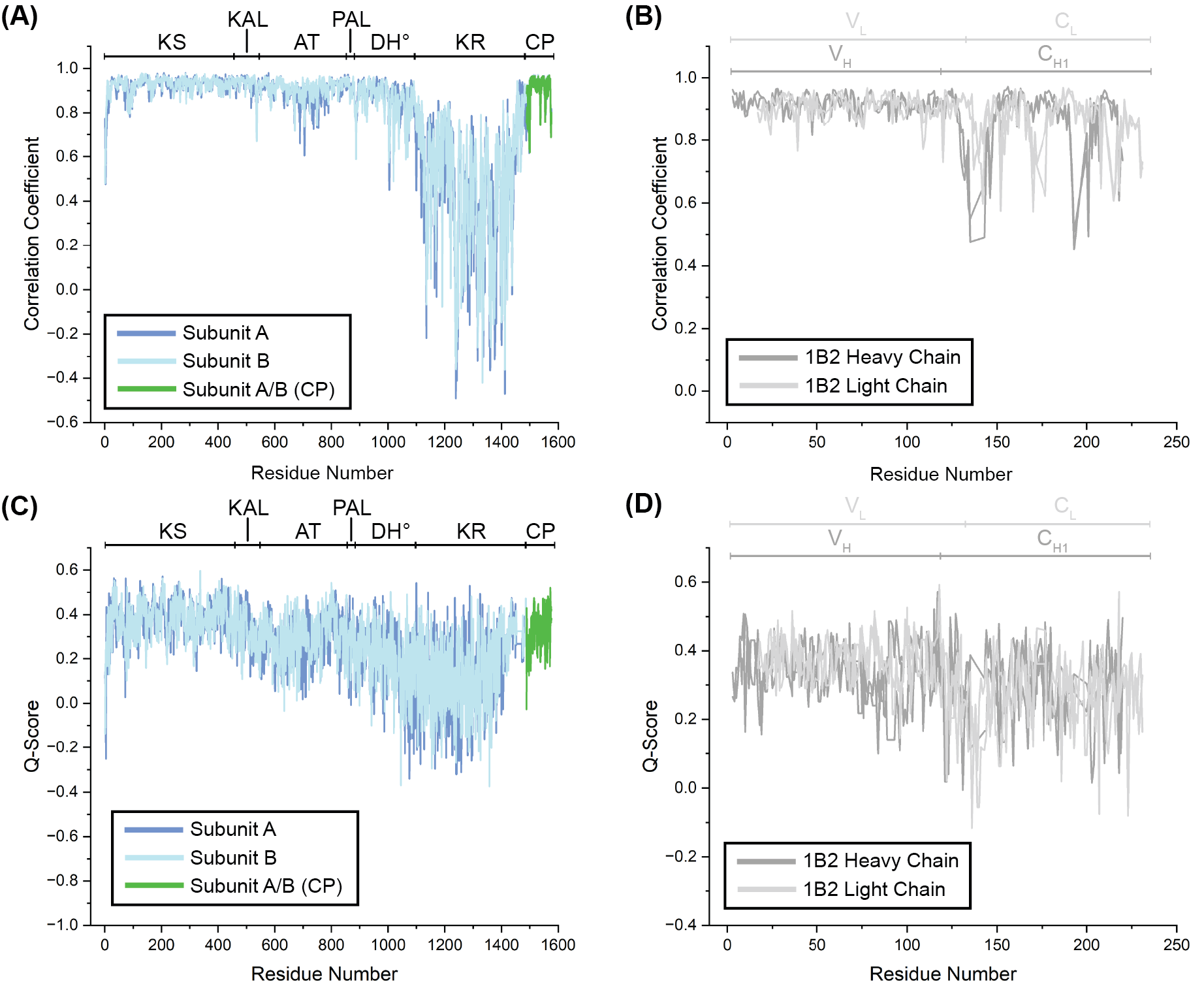


**Fig. S17.** Cryo-EM model validation of crosslinked M1-TEII in complex with F_ab_ 1B2 (CL-M1-TEII-1B2; PDB 9PC6). Per-residue cryo-EM map and model correlation coefficients (CCs) determined by Phenix real-space refinement(11) are plotted for each subunit of **(A)** M1-DD and **(B)** F_ab_ 1B2. Likewise, per-residue Q-score assessments of atom resolvability within the cryo-EM map(12) are plotted for each subunit of **(C)** M1-DD and **(D)** F_ab_ 1B2. (KAL = KS-AT linker; PAL = post-AT linker; V_L_ = variable domain of the light chain; C_L_ = constant domain of the light chain; V_H_ = variable domain of the heavy chain; C_H1_ = first constant domain of the heavy chain.) AlphaFold 3 modeling and reference to structural homologs in the PDB guided initial model building in regions of the model with CC ~0.8 or less (i.e., PDB 4LN9 for DH°, PDB 3SLK for KR, and PDB 6C9U for C_L_ and C_H1_)(13).


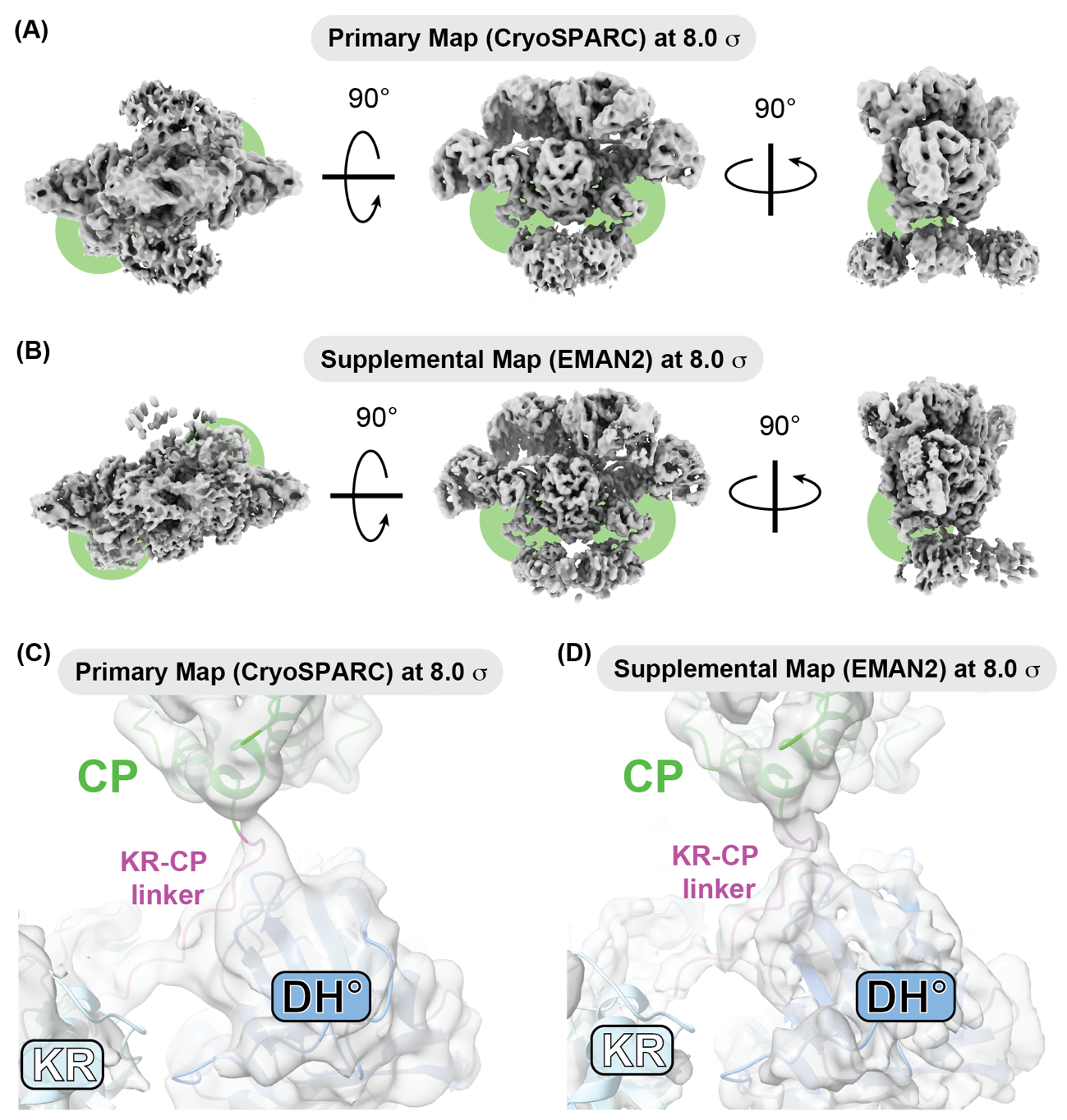


**Fig. S18.** Three different orientations of the **(A)** primary and **(B)** supplemental cryo-EM maps associated with the CL-M1-TEII-1B2 structure (Figs. S15–S17) contoured at 8.6 σ and 11.4 σ, respectively. Green circles highlight regions of CP domain density. **(C–D)** Close-up view of the KR-CP linker for the **(C)** primary and **(D)** supplemental maps at similar threshold (sigma) highlights slightly improved local resolution in the supplemental map. Sigma values were calculated from the entire cryo-EM map volumes by the equation: σ = (threshold – mean) / standard deviation.

**
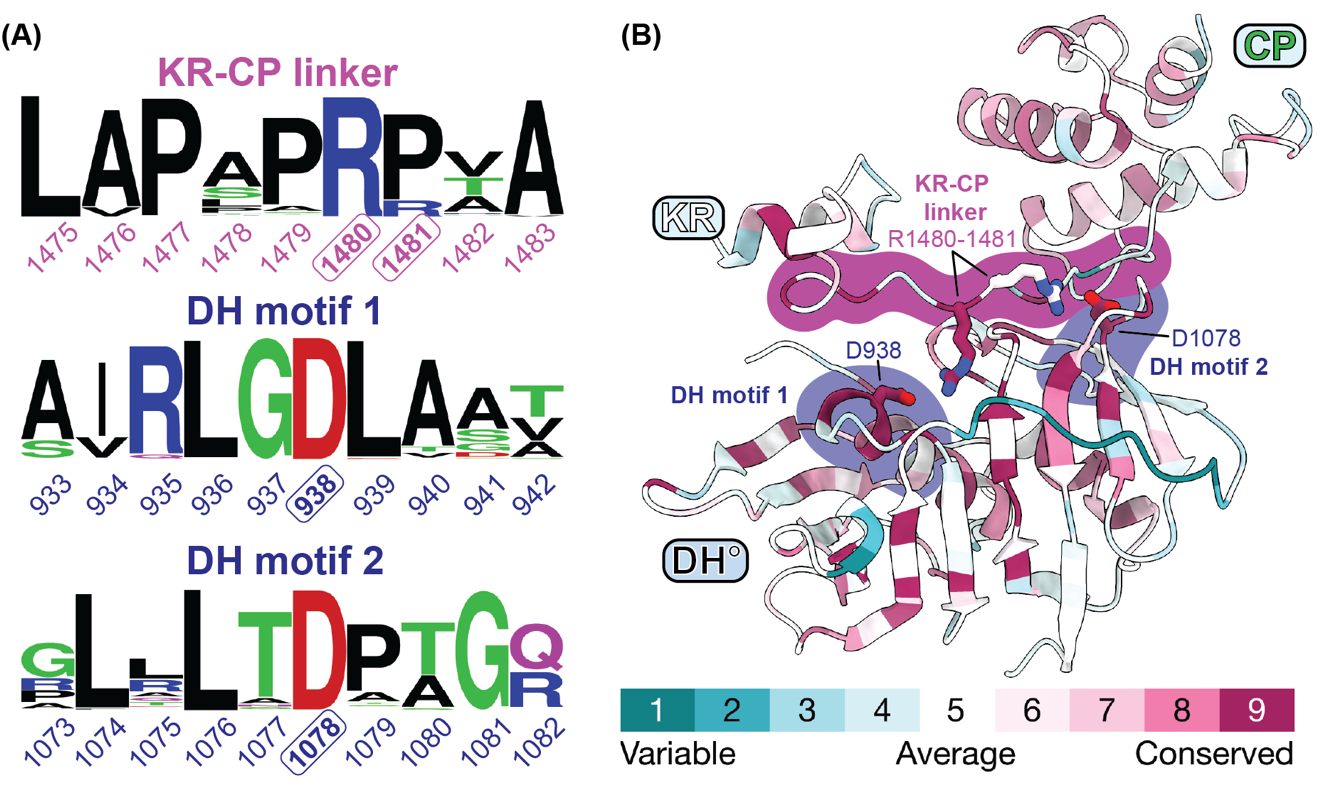
**

**Fig. S19. (A)** WebLogos displaying residue type and frequency at selected regions harboring putative Arg-Asp interactions (i.e., KR-CP linker and DH motifs 1 & 2) were generated from a multiple sequence alignment of 250 homologs of the DH°-KR-CP fragment of RIFS M1 (WP_013222547.1) identified by NCBI BLAST against the non-redundant protein sequences database(15, 16). From this analysis, three out of four of the residues putatively involved in electrostatics are invariant, whereas one of them (R1481) is often replaced by a Pro. **(B)** The ConSurf server was used to represent sequence conservation as a numerical color scale on the CL-M1-TEII-1B2 model (PDB 9PC6)(17). Residues involved in putative inter-subunit interactions between the KR-CP linker (R1480–R1481) and DH° domain (D938 from motif 1 and D1078 from motif 2; panel A) are shown. Sequence conservation scores for each residue were generated from a similar multiple sequence alignment of 250 homologs of the DH°-KR-CP fragment of RIFS M1.

**Fig. S20.** Comparison of the reaction rates of LM-M1 bimodule proteins: **(A)** WT LM-M1-DD, **(B)** LM-M1-DD-D1537R, **(C)** LM-M1-DD-D1677R, and **(D)** LM-M1-DD-D1537R/D1677R. The primary fragment ion of **d2′** (*m/z* = 73) was harnessed for quantification like in Figure 5B, except under an optimized gradient and fragmentation method (see **Single-Turnover Kinetic Analysis of LM-M1 Catalyzed Diketide Formation by LC-MS/MS-MRM**). (Note: D1537 is the equivalent of D938 in Fig. 4C, and D1677 is the equivalent of D1078 in Fig. 4C.) These data were fit to a single-phase exponential growth function in Origin 2023b to obtain approximate rate constants (*k_obs_*). Error bars represent the standard deviation of three technical replicates (n = 3) for each enzymatic reaction. A corresponding negative control reaction was analyzed featuring a version of LM-M1-DD whose catalytic Cys of the KS domain was genetically converted to Ala (i.e., LM-M1-DD(C802A), Fig. 5B).

**
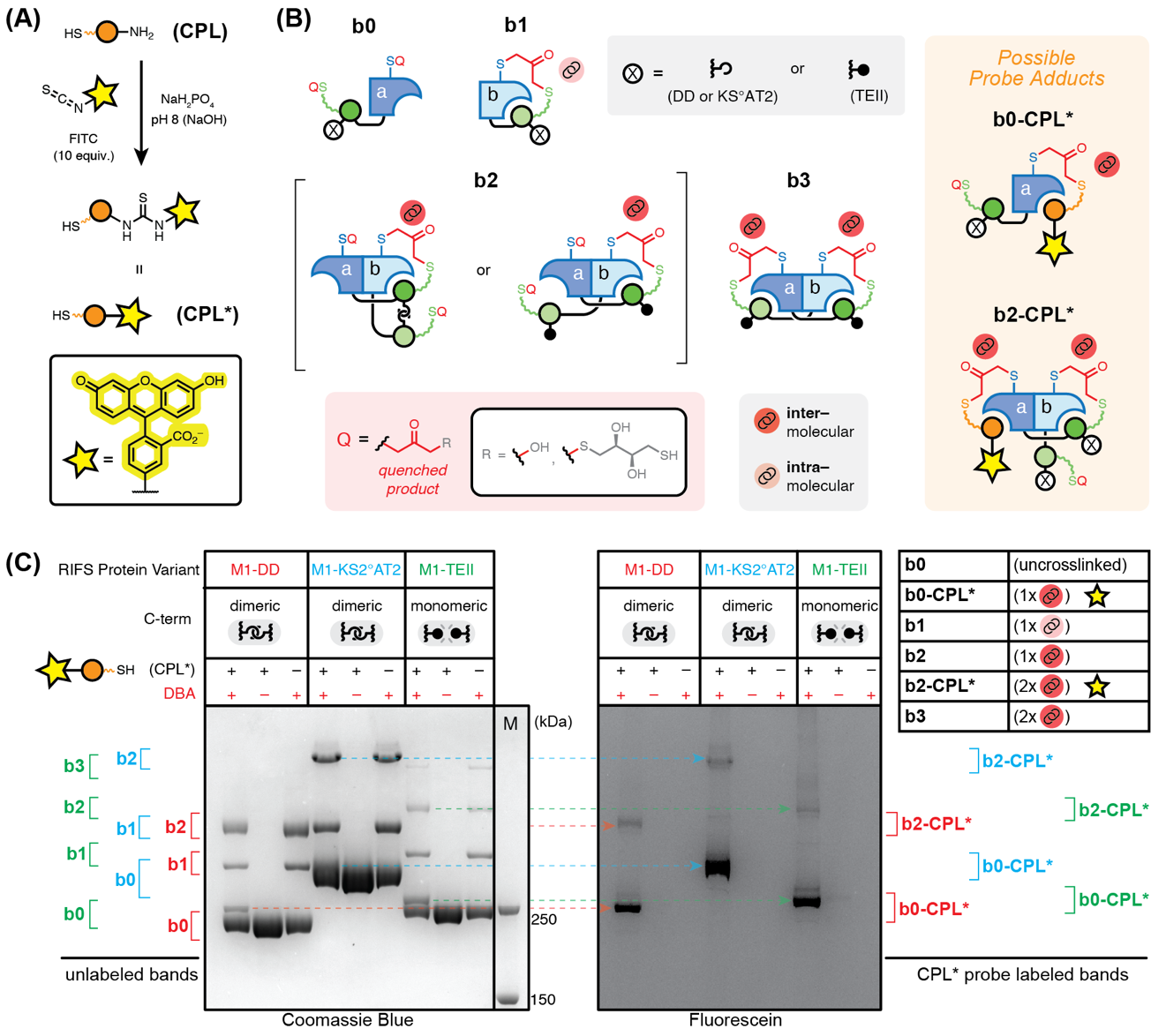
**

**Fig. S21.** Probing the effect of the native C-terminal dimerization motif of RIFS M1 on its module conformations by DBA crosslinking. The C-terminal DD from M1-DD was replaced with the KS-AT didomain from RIFS M2 while preserving the natural linkage between M1 and M2 in RifA (WP_013222547.1). To minimize competing crosslinking reactions between the CP of M1 (CP1) and the KS from M2 (KS2), the catalytic Cys of KS2 was genetically modified to Ala (C1767A). This protein is hereafter referred to as M1-KS2°AT2 and was isolated in its *holo*-form for DBA crosslinking analysis alongside *holo*-form M1-DD and M1-TEII. **(A)** Prior to crosslinking, a standalone *holo*-form CP from the LM (CPL) was reacted with fluorescein isothiocyanate (FITC) in sodium phosphate buffer (pH 8) to afford CPL*. Given the dual-specificity of the KS from M1 (KS1) for CPL and CP1, CPL* was included in DBA crosslinking reactions as a probe of vacant KS1 active sites, as reported earlier(18). **(B)** Cartoon depictions of the six anticipated products following addition of DBA to M1-DD, M1-KS2°AT2, and M1-TEII in the absence and presence of CPL* before denaturation by SDS-PAGE. **(C)** DBA or DMF (control) were added to the above proteins in the presence of 0.1 mM TCEP, 20 mM HEPES, and 300 mM citrate (pH 7.3) and analyzed by reducing SDS-PAGE (n=1, technical replicate; see **Methods** for details). Coomassie and fluorescence imaging suggested that species **b0** and **b2** for all proteins participated in a second crosslinking event with CPL* *in trans*, leading to **b0-CPL*** and **b2-CPL***. This observation was consistent with the KS-AT didomain of M2 enforcing a single KS1-CP1 crosslink, akin to the effect of DD in M1-DD (Fig. 2B,C). See **Protein Sequences** for CPL and M1-KS2°AT2 details.


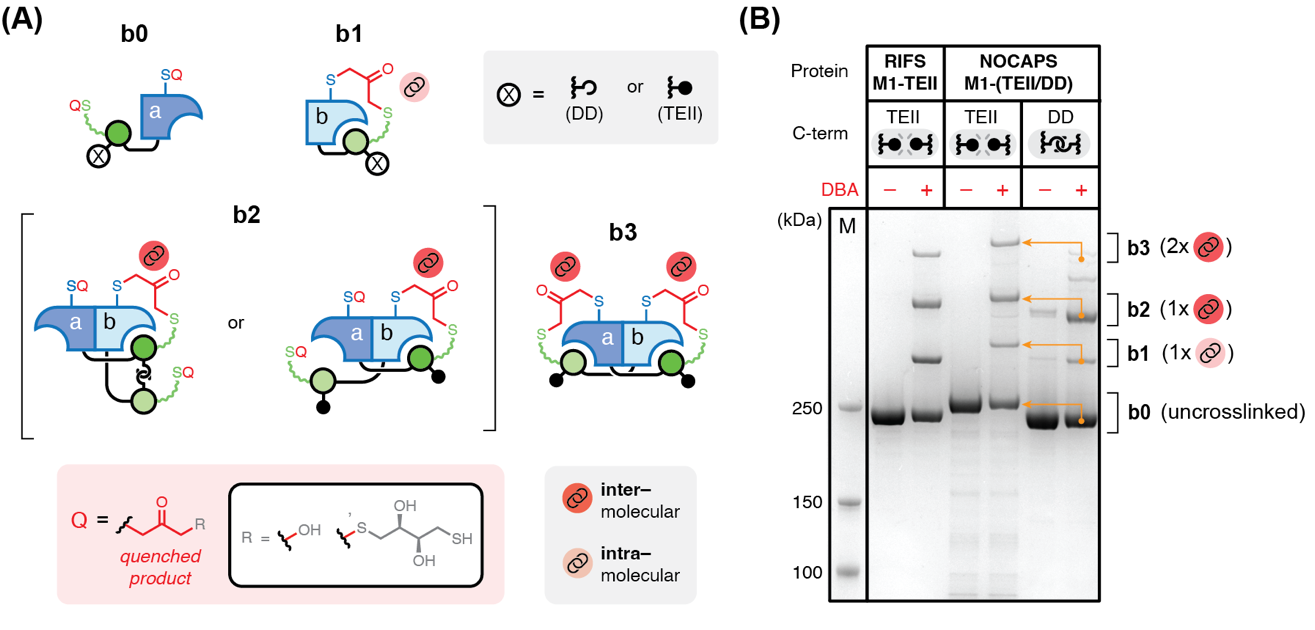


**Fig. S22.** DBA crosslinking analysis of RIFS M1-TEII and module 1 from the nocardiosis-associated PKS (NOCAPS) assembly line fused with either a C-terminal TEII (NOCAPS M1-TEII) or DD (NOCAPS M1-DD). While the DD in NOCAPS M1-DD is equivalent to the DD employed in M1-DD (i.e., from the erythromycin synthase), the TEII associated with NOCAPS M1-TEII is derived from *nocapC* in the nocardiosis-associated polyketide biosynthetic gene cluster(19) (see **Protein Sequences**). **(A)** Cartoon depictions of the four major products following addition of DBA to these proteins before denaturation by SDS-PAGE. **(B)** DBA or DMF (control) were added to the above proteins in the presence of 0.1 mM TCEP, 20 mM HEPES, and 300 mM citrate (pH 7.3) and analyzed by reducing SDS-PAGE (n=1, technical replicate). The appearance of a band distribution in NOCAPS M1-TEII like that of RIFS M1-TEII pointed to an inhibitory role for DD in the formation of **b3**. Orange circles connected by arrows indicate the positions where protein bands are expected to occur in crosslinked NOCAPS M1-DD (+ DBA) if they differ only from the indicated bands in crosslinked NOCAPS M1-TEII (+ DBA) by the mass difference between DD and TEII. While bands at these positions are observed for **b0**, **b1**, and **b2** in M1-DD (+ DBA), a relatively lower abundance band is observed at the position corresponding to **b3**.


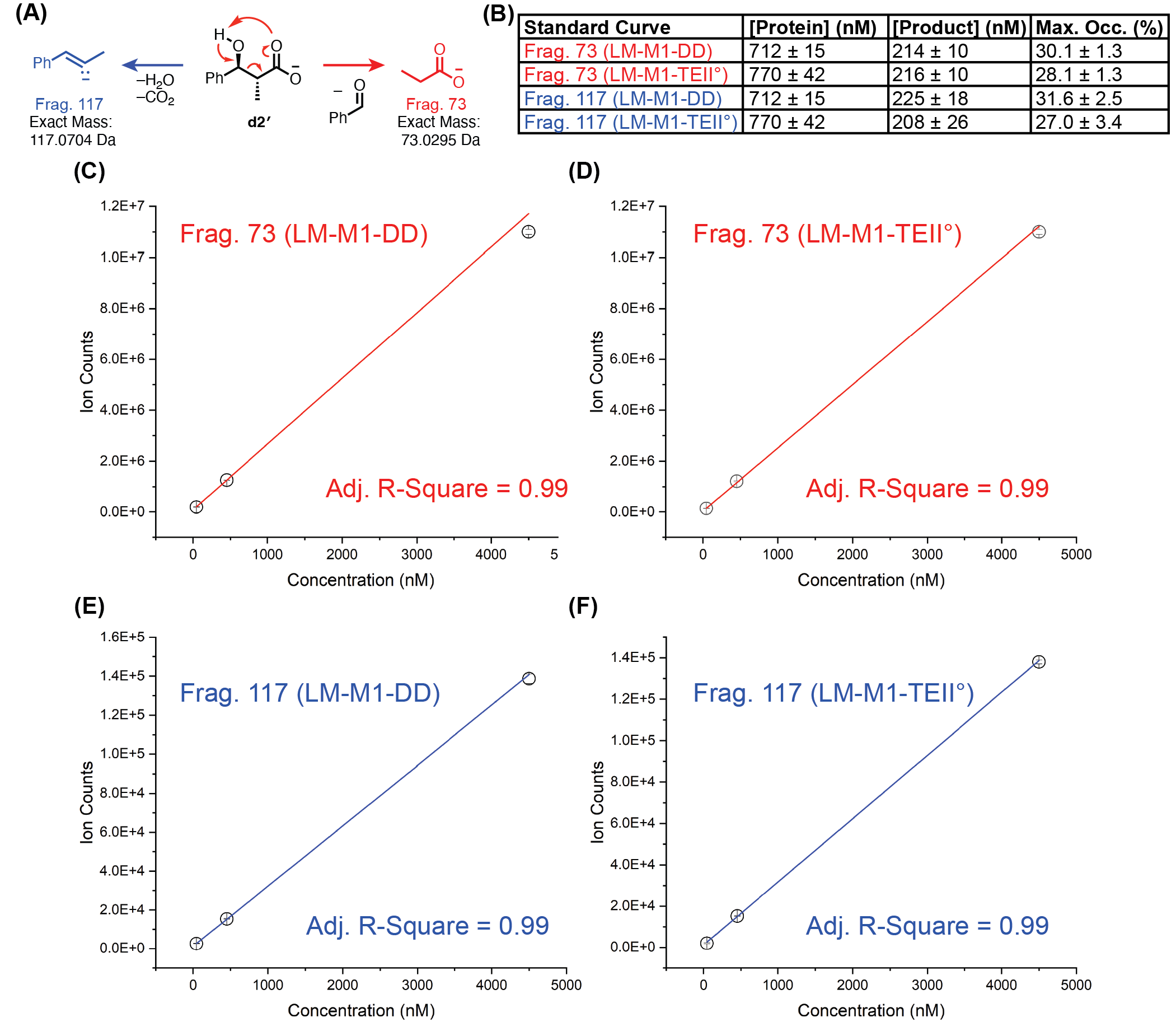


**Fig. S23.** LC-MS/MS-MRM quantitation of **d2′**. **(A)** Proposed fragmentation patterns for **d2′** based on detection of fragment ions at *m/z* 73 Da and 117 Da in negative ion mode. Standard curves for quantification of **d2′** in enzymatic reactions were generated by preparing **d2′** at 45 nM, 450 nM, and 4,500 nM in the presence of LM-M1-DD or LM-M1-TEII° in reaction buffer lacking ATP, NADPH, and MeMal-CoA (racemate). Standards were treated with KOH, heating, and formic acid in the same manner as enzymatic reactions prior to LC-MS/MS-MRM analysis. **(B)** The concentrations of **d2′** and maximum occupancy of **d2** (Fig. 5) for LM-M1-DD and LM-M1-TEII° were determined from **(C–F)** standard curves corresponding to the 73 Da and 117 Da fragment ions quantified in the presence of each protein. The protein concentrations were determined by absorbance values measured on a NanoDrop OneC (pedestal) and the calculated molecular weights and extinction coefficients of *holo*-form LM-M1-DD (238,589 Da; ε = 216,230 M^-1^cm^-1^) or LM-M1-TEII° (260,968 Da; ε = 234,680 M^-1^cm^-1^). Note: the reported concentrations of protein and product (**d2′**) in panel B reflect their final concentrations after buffer exchange and sample workup (see **Quantification of LM-M1 Bound Diketide by LC-MS/MS-MRM**). Error bars represent the standard deviation of three technical replicates (n = 3) for each **d2′** standard.

**
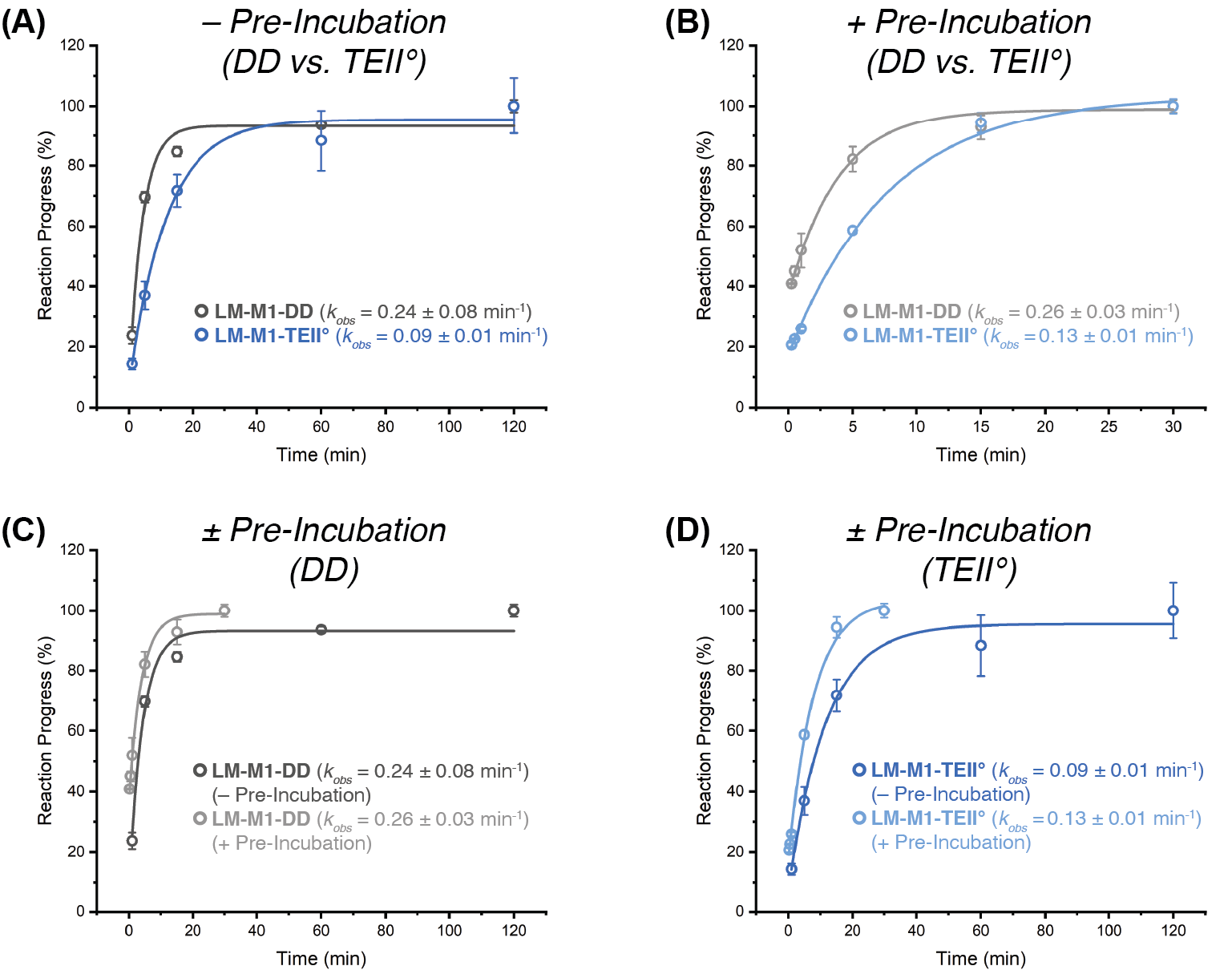
**

**Fig. S24.** The effect of pre-incubating ATP/Mg^2+^ and benzoate with LM-M1 harboring a C-terminal DD or TEII° on the rates of **d2** formation (Fig. 5). Black/gray traces indicate reactions with LM-M1-DD ± pre-incubation, respectively. Blue/light blue traces indicate reactions with LM-M1-TEII° ± pre-incubation, respectively (see **Single-Turnover Kinetic Analysis of LM-M1 Catalyzed Diketide Formation by LC-MS/MS-MRM**). Comparison of **(A)** the rates of **d2** formation by LM-M1-DD and LM-M1-TEII° without pre-incubation (replotted from Fig. 5B), **(B)** the rates of **d2** formation by LM-M1-DD and LM-M1-TEII° with pre-incubation, **(C)** the rates of **d2** formation by LM-M1-DD ± pre-incubation, and **(D)** the rates of **d2** formation by LM-M1-TEII° ± pre-incubation. Rate constants (*k_obs_)* were obtained by fitting these data to a single-phase exponential growth function in Origin 2023b. In both cases (± pre-incubation), LM-M1-DD catalyzed **d2** formation faster than LM-DD-TEII° by a factor ≥2. Interestingly, pre-incubation had no effect on the rate of **d2** formation by LM-M1-DD, whereas it had a measurable effect on that of LM-M1-TEII°. This implied that LM-M1-TEII° was more rate-limited by LM-catalyzed benzoylation than LM-M1-DD. We note that these enzymatic reactions contained elevated amounts of citrate (100 mM)—unlike prior kinetic measurements of LM-M1(20)—which is an additive known to enhance the catalytic activity of PKS modules *in vitro*(4, 21). Error bars represent the standard deviation of three technical replicates (n = 3) for each enzymatic reaction.


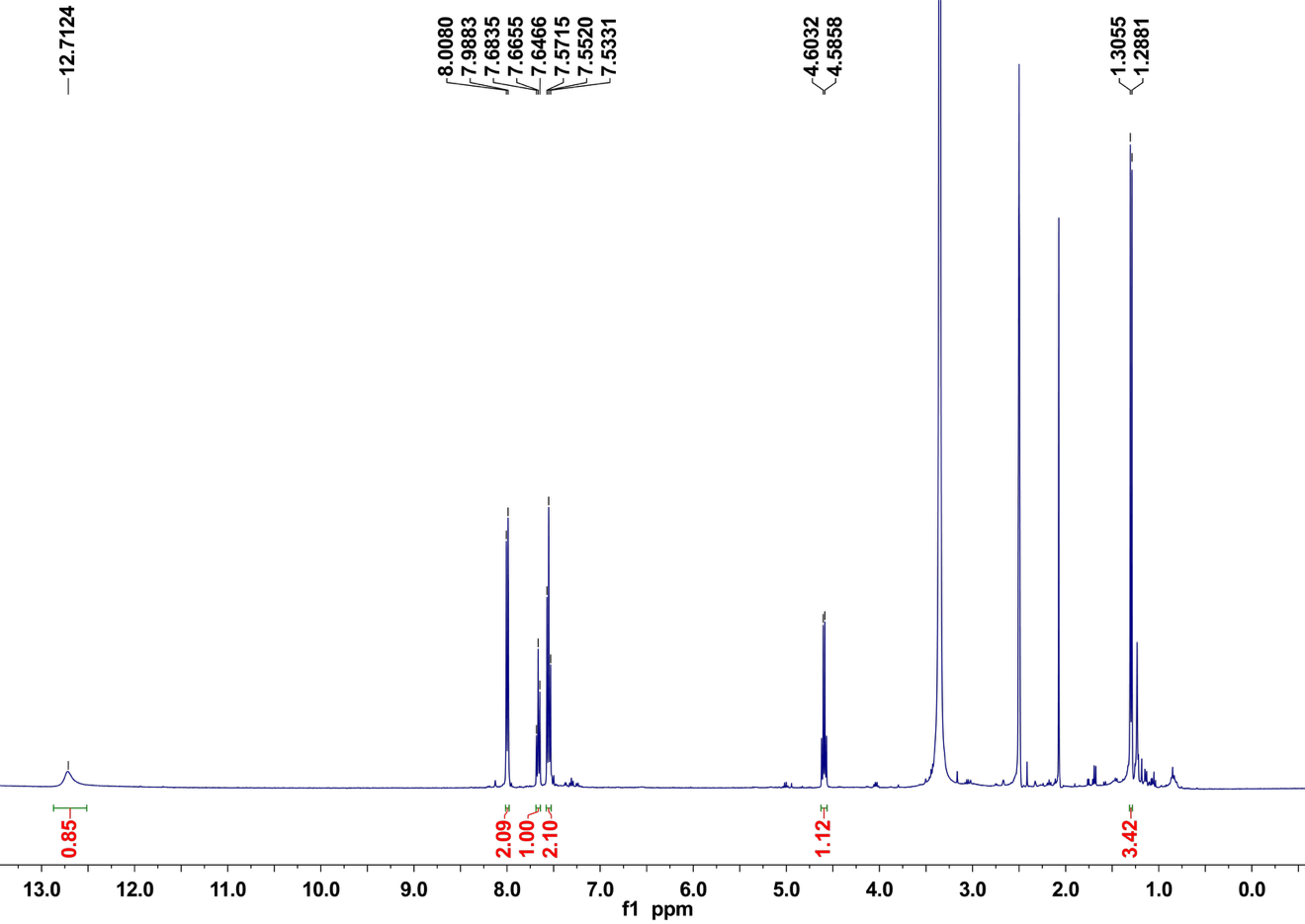


**Fig. S25.** ^1^H NMR spectrum of **keto-d2′** in DMSO-*d*_6_ (400 MHz).

**
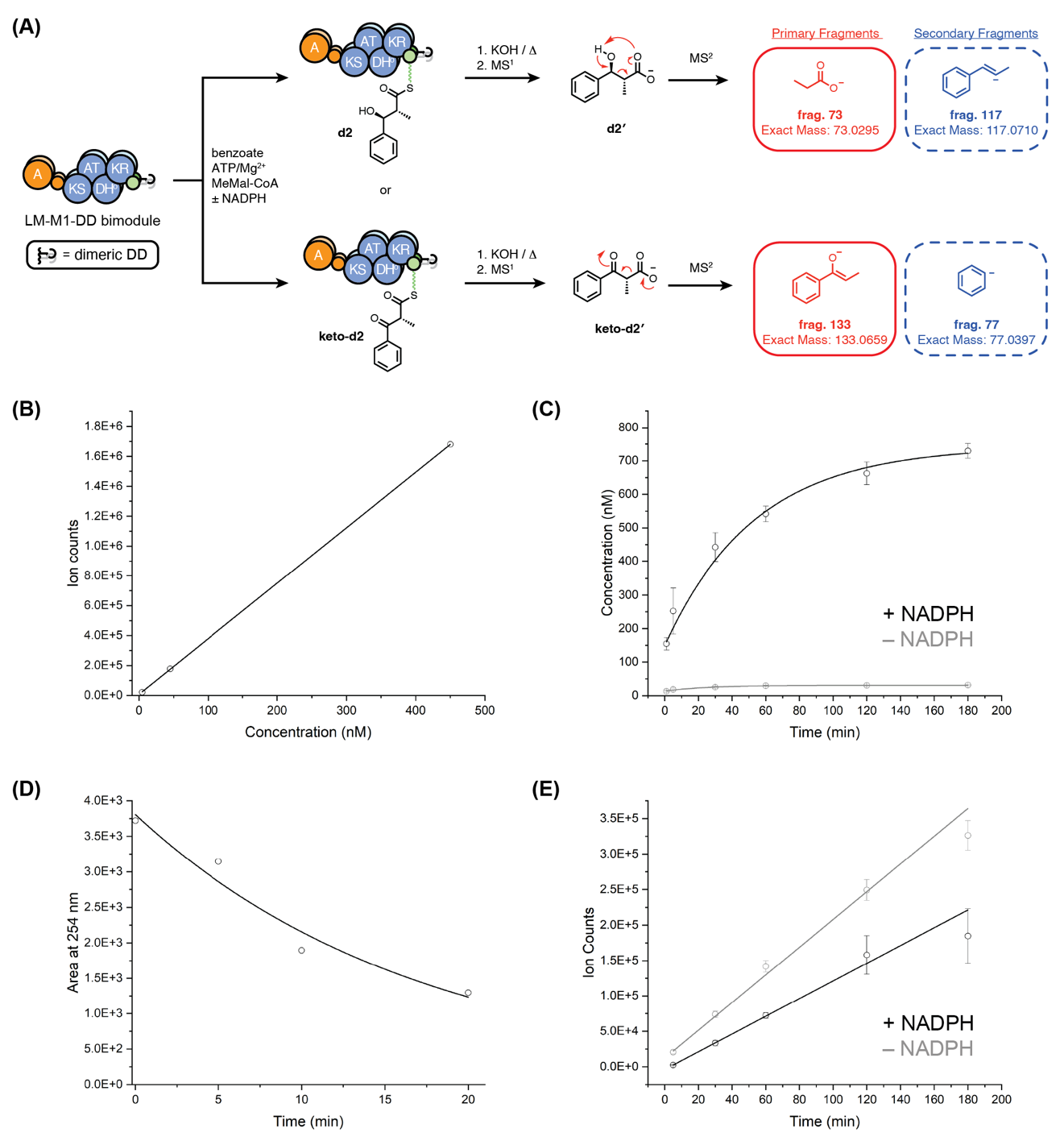
**

**Fig. S26.** LC-MS/MS-MRM quantitation of **d2′** and **keto-d2′**. **(A)** Enzymatic production of covalently anchored diketides (**d2** and **keto-d2**) by the RIFS LM-M1-DD bimodule. KOH and heat treatment of LM-M1-DD liberated **d2′** and **keto-d2′** which were detected by LC-MS/MS-MRM. The primary fragment ions of **d2′** (*m/z* = 73, propionate) and **keto-d2′** (*m/z* = 133) were harnessed for quantification, respectively. **(B)** Standard curve corresponding to the 73 Da fragment ion quantified in the absence of protein. **(C)** The rates of **d2** formation by LM-M1-DD ± NADPH. The trace amounts of **d2′** detected in the NADPH-deficient samples may have stemmed from the recombinant protein carrying NADPH and/or biosynthetic **d2** from *E. coli* BAP1 post purification. The data were fit to a single-phase exponential growth function in Origin 2023b to obtain apparent rate constants (*k_obs_*). (Differences in the *k_obs_* observed here relative to those reported in Figures 5B and S20 are believed to reflect differences in the quality of protein between different preparations. Therefore, we limited our discussion of LC-MS/MS-MRM–based kinetic data to relative rates in each experiment, without invoking absolute rate differences between experiments.) LC-MS/MS-MRM quantification of **d2′** (Fig. S23) was used to determine the maximum fractional occupancy of each protein with **d2** in enzymatic reactions. The LM-M1-DD bimodule showed a 32% maximum occupancy (~700 nM, relative to 2,200 nM LM-M1-DD post workup), comparable to the occupancies reported in Figures 5B and S23. **(D)** Examination of the stability of **keto-d2′** under alkaline hydrolysis conditions. Briefly, 20 µL of **keto-d2′** standard aliquots were treated with KOH, heating, and formic acid in the same manner as above (see **Single-Turnover Kinetic Analysis of LM-M1 Catalyzed Diketide Formation by LC-MS/MS-MRM**) for different durations (5 min, 10 min, and 20 min). The resulting sample mixtures were subjected to HPLC analysis in the same manner as above (see **Synthesis of Diketide 2-methyl-3-oxo-3-phenylpropanote**). Gradual decline in the peak area at 254 nm over time indicated that **keto-d2′** was unstable under these conditions, therefore preventing accurate determination of **keto-d2** occupancy in the enzymatic reactions. **(E)** The relative progress of **keto-d2** formation by LM-M1-DD ± NADPH. Based on previous reports of ~20–50% occupancy of a PKS module with its growing intermediate(5, 20), a ≤20% occupancy of the **keto-d2** intermediate would accord with the 30% **d2** occupancy in the homodimeric protein (Figs. 5B and S23). This distribution could be explained by conformational regulation of ketoreduction and/or KR-catalyzed equilibration between the reduced (**d2**) and unreduced (**keto-d2**) states. If indeed only one subunit can become acylated with **d2** or **keto-d2** after a single catalytic cycle, then a <50% combined occupancy should be indicative of a fraction of the recombinant protein which was catalytically inactive. Error bars represent the standard deviation of three technical replicates (n = 3) for each enzymatic reaction (panels C and E). Data in panels B and D correspond to a single technical replicate for each data point.

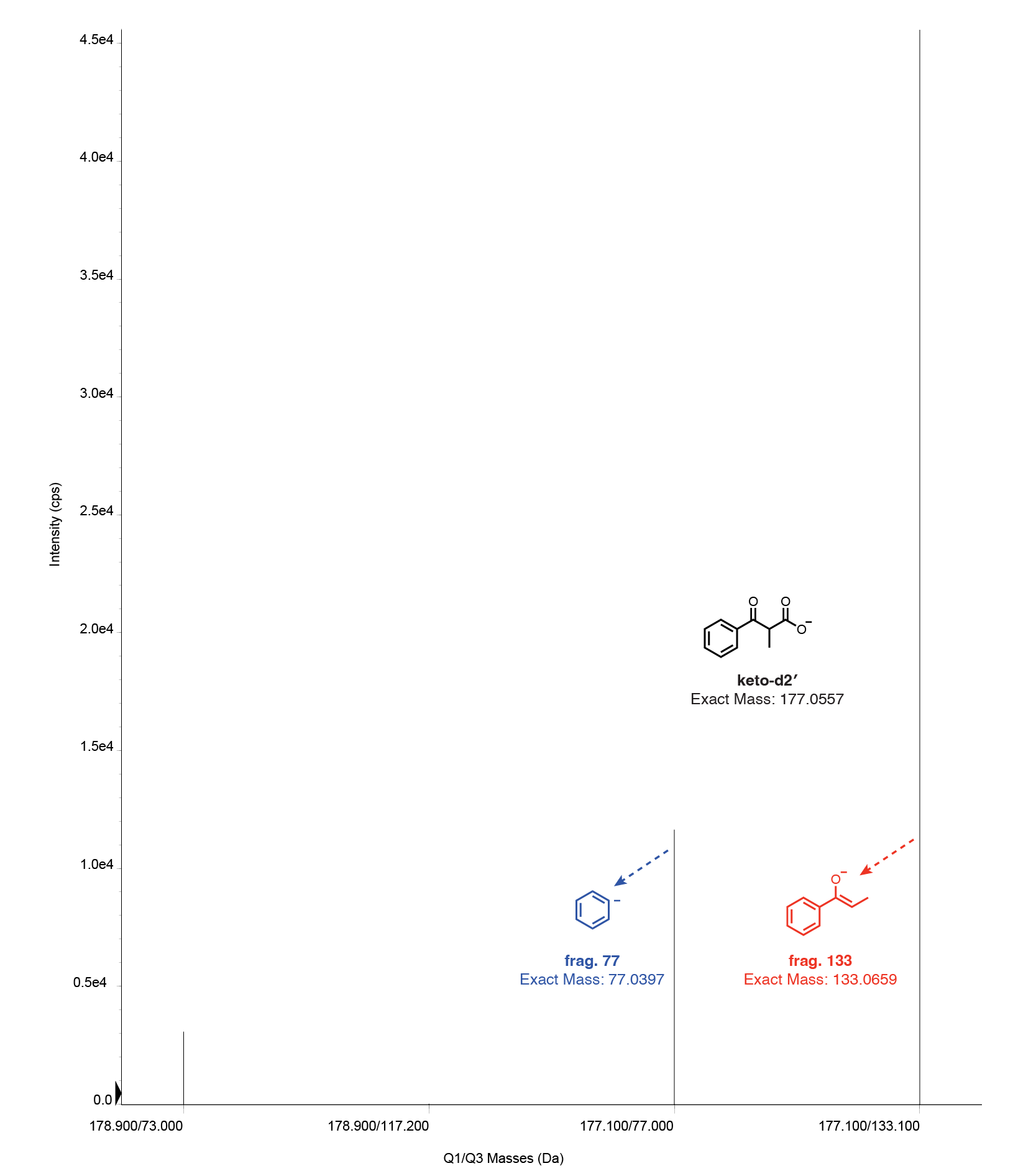


**Fig. S27.** MS/MS spectrum of hydrolyzed unreduced diketide **keto-d2**′ (Figs. S25–S26).

**
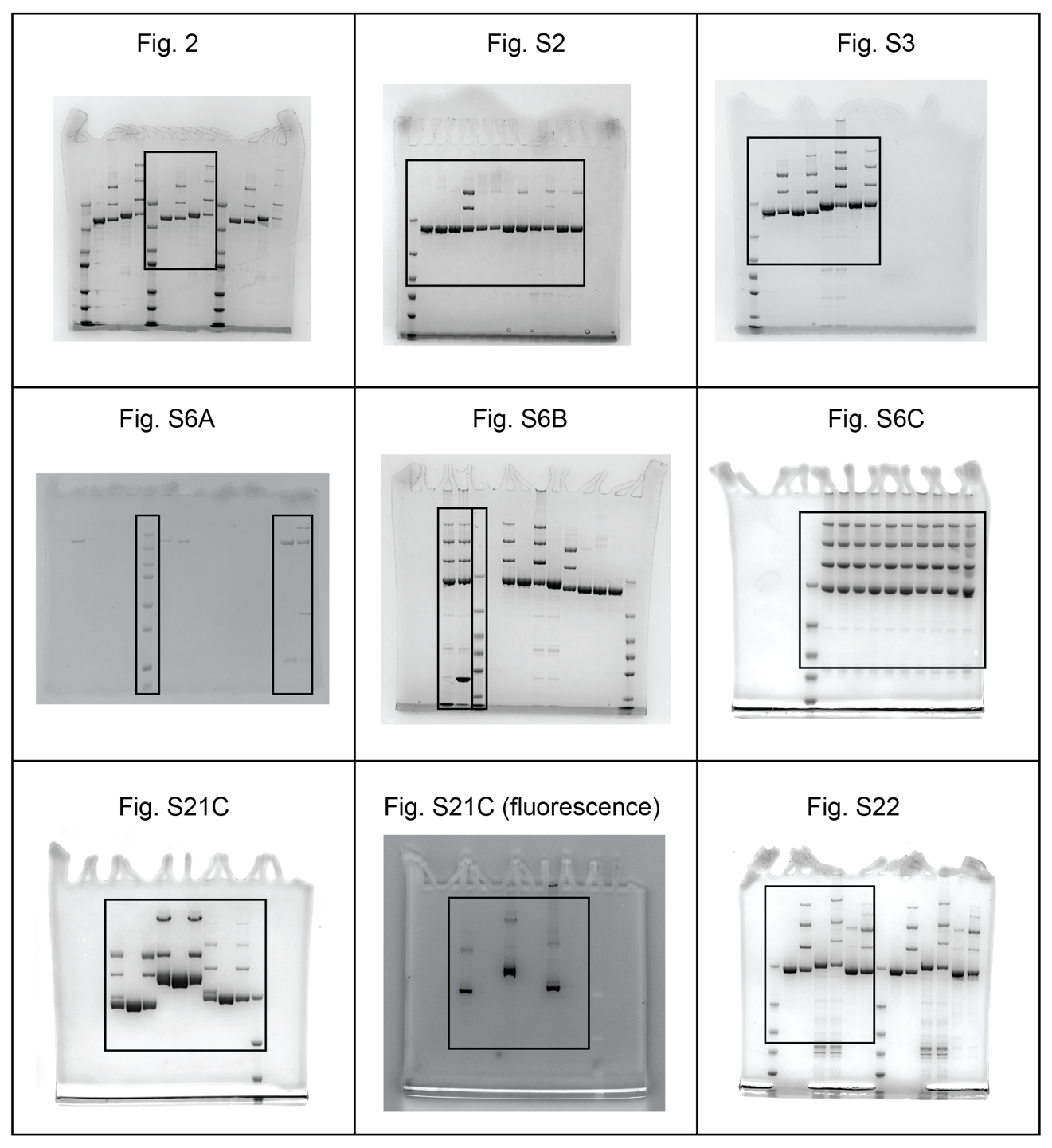
**

**Fig. S28.** Uncropped gel images used in this work. Black boxes are drawn to delineate where images were cropped in each figure. Aside from cropping, the only modification made to these images was uniform conversion from color to grayscale. All gel images were obtained after staining with InstantBlue® Coomassie Protein Stain (abcam, ab119211) except for the gel labeled “Fig. S21C (fluorescence)” which was imaged using the fluorescein excitation and emission wavelengths of the iBright FL1000 gel imager (Invitrogen) prior to Coomassie staining.

**Supporting Tables**

Table S1. Primary biosynthetic proteins used in this study. See accompanying Table S2 for the color-coded domain/linker key, Table S3 for plasmid details, and primary amino acid sequences below (Protein Sequences).

| **Protein**  **(Plasmid)** | **Description**  **(Purpose)** | **Domain Architecture** |
| --- | --- | --- |
| RIFS LM-M1-TEII°  (pAM6) | RIFS LM-M1 bimodule with C-terminal TEII (a.k.a. RifR) genetically inactivated by S2316A and separated by a (G_4_S)_8_ linker  (used for kinetic analysis) | A-CPL-KS-AT-DH°-KR-CP1-(G_4_S)_8_-TEII°-His_6_ |
| RIFS CPL  (pCL3) | RIFS CP domain from the loading module (CPL)  (used for fluorescent probe construction and crosslinking) | His_6_-CPL |
| RIFS M1-KS2°AT2  (pCL62) | RIFS M1 with N-terminal docking domain from the 6-deoxyerythronolide B synthase and C-terminal KS-AT didomain from RIFS M2 with native M1-M2 linker and a genetically inactivated KS from M2 (C1767A)  (used for crosslinking) | (DD3)-KS-AT-DH°-KR-CP1-KS2°-AT2-His_6_ |
| RIFS LM-M1-DD  (pDC42) | RIFS LM-M1 bimodule with C-terminal docking domain from 6-deoxyerythronolide B synthase  (used for kinetic analysis) | A-CPL-KS-AT-DH°-KR-CP1-(DD2)-His_6_ |
| RIFS M1-DD  (pDC43) | RIFS M1 with N- and C-terminal docking domains from 6-deoxyerythronolide B synthase  (used for crosslinking/cryo-EM) | (DD3)-KS-AT-DH°-KR-CP1-(DD2)-His_6_ |
| NOCAPS M1-DD  (pDC57) | NOCAPS M1 with N- and C-terminal docking domains from the 6-deoxyerythronolide B synthase  (used for crosslinking) | (DD3)-KS-AT-DH-KR-CP1-(DD2)-His_6_ |
| NOCAPS M1-TEII  (pDC81) | NOCAPS M1 with C-terminal TEII derived from NocapC  (used for crosslinking) | KS-AT-DH-KR-CP1-TEII-His_6_ |
| RIFS M1-TEII  (pDC85) | RIFS M1 with N-terminal docking domain from 6-deoxyerythronolide B synthase and C-terminal TEII (a.k.a. RifR) separated by a (G_4_S)_2_ linker  (used for crosslinking/cryo-EM) | (DD3)-KS-AT-DH°-KR-CP1-(G_4_S)_2_-TEII-His_6_ |
| RIFS M1-ΔDD  (pDC155) | RIFS M1 with N- and C-terminal docking domains from the 6-deoxyerythronolide B synthase and a 38-residue deletion (Δ1588–1625) within the C-terminal docking domain corresponding to its dimeric α-helical fragment  (used for crosslinking) | (DD3)-KS-AT-DH°-KR-CP1-(ΔDD2)-His_6_ |

**Table S2.** Color-coded domain/linker key for protein sequences used in this study.

| KS = ketosynthase |
| --- |
| AT = acyltransferase |
| KR = ketoreductase |
| DH = dehydratase |
| DH° = inactive dehydratase |
| CP = carrier protein |
| TEII = type II thioesterase |
| RIFS LM (RifA) |
| RIFS M1 (RifA) / NOCAPS M1 (NocapA) |
| RIFS M2 (RifA) |
| C-terminal Docking Domain of DEBS1 (a.k.a. EryAI) Module 2 (DD2) |
| N-terminal Docking Domain of DEBS2 (a.k.a. EryAII) Module 3 (DD3) |
| TEII (RifR) / TEII (NocapC) |
| F_ab_ 1B2 |
| Linkers/Tags |

Table S3. Plasmids used in this study (Km = kanamycin; Cb = carbenicillin; Am = ampicillin).

| **Plasmid** | **Encoded Protein** | **Antibiotic** | **Reference** |
| --- | --- | --- | --- |
| pAM6 | RifA LM-M1-TEII(S2316A)-His_6_ (**RIFS** **LM-M1-TEII°**) | Cb/Am | This study |
| pCL3 | RifA His_6_-CPL (**RIFS CPL**) | Km | This study |
| pCL17 | RifA LM-M1-DD(C802A)-His_6_ | Cb/Am | This study |
| pCL26 | RifA LM-M1-DD(D1537R)-His_6_ | Cb/Am | This study |
| pCL27 | RifA LM-M1-DD(D1537R/D1677R)-His_6_ | Cb/Am | This study |
| pCL28 | RifA LM-M1-DD(D1677R)-His_6_ | Cb/Am | This study |
| pCL62 | RifA M1-KS2°AT2-His_6_ (**RIFS** **M1-KS2°AT2**) | Cb/Am | This study |
| pDC42 | RifA LM-M1-DD-His_6_ (**RIFS** **LM-M1-DD**) | Cb/Am | This study |
| pDC43 | RifA M1-DD-His_6_ (**RIFS** **M1-DD**) | Cb/Am | This study |
| pDC57 | NocapA M1-DD (**NOCAPS M1-DD**) | Cb/Am | This study |
| pDC81 | NocapA M1-TEII (**NOCAPS M1-TEII**) | Cb/Am | This study |
| pDC85 | RifA M1-TEII-His_6_ (**RIFS** **M1-TEII**) | Cb/Am | This study |
| pDC147 | RifA M1-TEII(S1717A)-His_6_ | Cb/Am | This study |
| pDC155 | RifA M1-DD(Δ1588–1625)-His_6_ (**RIFS** **M1-ΔDD**) | Cb/Am | This study |
| pRSG56 | His_6_-Sfp | Km | (22) |
| pRW9 | RifA M1-DD(C203A)-His_6_ | Cb/Am | This study |
| pRW20 | RifA M1-DD(C254A)-His_6_ | Cb/Am | This study |
| pRW22 | RifA M1-DD(C203S/C254A)-His_6_ | Cb/Am | This study |
| pRW24 | RifA M1-DD(C203S)-His_6_ | Cb/Am | This study |
| n/a | F_ab_ 1B2-His_6_ (**1B2**) | Cb/Am | (23) |

**Table S4.** Cryo-EM data collection, refinement, and validation statistics.

| *Structure*  EMPIAR ID  EMDB ID PDB ID | *Transacylation-mode*  EMPIAR-13347  EMDB-71445  PDB 9PAT | *Elongation-mode*  EMPIAR-13347  EMDB-71446  PDB 9PAV | *CL-M1-TEII-1B2*  EMPIAR-13348  EMDB-71497  PDB 9PC6 |
| --- | --- | --- | --- |
| **Data collection and processing**  Sample  Microscope  Camera | M1-DD-1B2  Krios G3i  K3 | M1-DD-1B2  Krios G3i  K3 | CL-M1-TEII-1B2  Glacios  Falcon4 |
| Magnification | 81,000 | 81,000 | 130,000 |
| Voltage (kV)  Exposure time (s)  Dose rate (e– pixel^-1^ s^-1^) | 300  5.72  10.58 | 300  5.72  10.58 | 200  4.00  6.65 |
| Electron exposure (e–/Å^2^) | 50 | 50 | 50 |
| Defocus range (μm) | -0.5 – -3.0 | -0.5 – -3.0 | -0.5 – -3.0 |
| Pixel size (Å)  Number of micrographs | 1.10  10,005 | 1.10  10,005 | 0.73  7,631 |
| Symmetry imposed | C1 | C1 | C1 |
| Initial particle images (no.) | 1,294,603 | 1,294,603 | 204,349 |
| Final particle images (no.) | 37,950 | 177,509 | 91,575 |
| Map resolution (Å)  (FSC threshold) | 3.96  (0.143) | 3.22  (0.143) | 3.96  (0.143) |
| Map resolution range (Å) | 3.52–40.14 | 2.84–32.34 | 3.51–9.92 |
|  |  |  |  |
| **Refinement** |  |  |  |
| Initial model used (PDB code) | N/A | N/A | N/A |
| Model resolution (Å)  (FSC threshold) | 3.96  (0.143) | 3.22  (0.143) | 3.96  (0.143) |
| Map sharpening *B* factor (Å^2^) | 47.8 | 77.6 | 71.7 |
| Model composition  Non-hydrogen atoms  Protein residues  Ligands | 25,653  3,465  0 | 25,647  3,465  0 | 29,408  3,977  0 |
| R.m.s. deviations  Bond lengths (Å)  Bond angles (°) | 0.003  0.538 | 0.004  0.569 | 0.002  0.583 |
| Validation  MolProbity score  Clashscore  Poor rotamers (%) | 2.24  11.07  3.89 | 2.03  8.40  2.92 | 2.10  11.73  3.39 |
| Ramachandran plot  Favored (%)  Allowed (%)  Disallowed (%) | 96.50  3.50  0.00 | 96.59  3.41  0.00 | 97.44  2.56  0.00 |

**Protein Sequences**

*RIFS CPL (pCL3)* **|** His_6_-CPL

| MGSSHHHHHHSSGLLPRGSHMLRARLSGLDERAQCELLEDLVRTQAADVLGQPVPDGRAFRDLGFTSLAIVELRNRLTEHTGLWLPASAVFDHPTPAALAARVRAELLG |
| --- |

*RIFS M1 with N-terminal docking domain from 6-deoxyerythronolide B synthase and* *C-terminal KS-AT didomain from RIFS M2 with native M1-M2 linker (****RIFS M1-KS2°AT2****, pCL62)* **|** (DD3)-KS-AT-DH°-KR-CP1-KS2°-AT2-His_6_

| MASTDSEKVAEYLRRATLDLRAARQRIRELEGEPIAIVGMACRLPGGVASPEDLWRLVAERVDAVSEFPGDRGWDLDSLIDPDRERAGTSYVGQGGFLHDAGEFDAGFFGISPREAVAMDPQQRLLLETSWEALENAGVDPIALKGTDTGVFSGLMGQGYGSGAVAPELEGFVTTGVASSVASGRVSYVLGLEGPAVTVDTACSSSLVAMHLAAQALRQGECSMALAGGVTVMATPGSFVEFSRQRALAPDGRCKAFAAAADGTGWSEGVGVVVLERLSVARERGHRILAVLRGSAVNQDGASNGLTAPNGLSQQRVIRRALAAAGLAPSDVDVVEAHGTGTTLGDPIEAQALLATYGQERKQPLWLGSLKSNIGHAQAAAGVAGVIKMVQALRHETLPPTLHVDKPTLEVDWSAGAIELLTEARAWPRNGRPRRAGVSSFGVSGTNAHLILEEAPAEEPVAAPELPVVPLVVSARSTESLSGQAERLASLLEGDVSLTEVAGALVSRRAVLDERAVVVAGSREEAVTGLRALNTAGSGTPGKVVWVFPGQGTQWAGMGRELLAESPVFAERIAECAAALAPWIDWSLVDVLRGEGDLGRVDVLQPACFAVMVGLAAVWESVGVRPDAVVGHSQGEIAAACVSGALSLEDAAKVVALRSQAIAAELSGRGGMASVALGEDDVVSRLVDGVEVAAVNGPSSVVIAGDAHALDATLEILSGEGIRVRRVAVDYASHTRHVEDIRDTLAETLAGISAQAPAVPFYSTVTSEWVRDAGVLDGGYWYRNLRNQVRFGAAATALLEQGHTVFVEVSAHPVTVQPLSELTGDAIGTLRREDGGLRRLLASMGELFVRGIDVDWTAMVPAAGWVDLPTYAFEHRHYWLEPAEPASAGDPLLGTVVSTPGSDRLTAVAQWSRRAQPWAVDGLVPNAALVEAAIRLGDLAGTPVVGELVVDAPVVLPRRGSREVQLIVGEPGEQRRRPIEVFSREADEPWTRHAHGTLAPAAAAVPEPAAAGDATDVTVAGLRDADRYGIHPALLDAAVRTVVGDDLLPSVWTGVSLLASGATAVTVTPTATGLRLTDPAGQPVLTVESVRGTPFVAEQGTTDALFRVDWPEIPLPTAETADFLPYEATSAEATLSALQAWLADPAETRLAVVTGDCTEPGAAAIWGLVRSAQSEHPGRIVLADLDDPAVLPAVVASGEPQVRVRNGVASVPRLTRVTPRQDARPLDPEGTVLITGGTGTLGALTARHLVTAHGVRHLVLVSRRGEAPELQEELTALGASVAIAACDVADRAQLEAVLRAIPAEHPLTAVIHTAGVLDDGVVTELTPDRLATVRRPKVDAARLLDELTREADLAAFVLFSSAAGVLGNPGQAGYAAANAELDALARQRNSLDLPAVSIAWGYWATVSGMTEHLGDADLRRNQRIGMSGLPADEGMALLDAAIATGGTLVAAKFDVAALRATAKAGGPVPPLLRGLAPLPRRAAAKTASLTERLAGLAETEQAAALLDLVRRHAAEVLGHSGAESVHSGRTFKDAGFDSLTAVELRNRLAAATGLTLSPAMIFDYPKPPALADHLRAKLFGSAANRPAEIGTAAAEEPIAIVAMACRFPGGVHSPEDLWRLVADGADAVTEFPADRGWDTDRLYHEDPDHEGTTYVRHGAFLDDAAGFDAAFFGISPNEALAMDPQQRLLLETSWELFERAAIDPTTLAGQDIGVFAGVNSHDYSMRMHRAAGVEGFRLTGGSASVLSGRVAYHFGVEGPAVTVDTAASSSLVALHMAVQALQRGECSMALAGGVMVMGTVETFVEFSRQRGLAPDGRCKAFADGADGTGWSEGVGLLLVERLSEAQRRGHQVLAVVRGSAVNSDGASNGLTAPNGPSQQRVIRKALAAAGLSTSDVDAVEAHGTGTTLGDPIEAEALLATYGQNRETPLWLGSVKSNLGHTQAAAGVAGVIKMVMAMRHGVLPRTLHVDRPSSYVDWSAGAVELLTEARDWVSNGHPRRAGVSSFGIGGTNAHVVLEEVAAPITTPQPEPAEFLVPVLVSARTAAGLRGQAGRLAAFLGDRTDVRVPDAAYALATTRAQLDHRAVVLASDRAQLCADLAAFGSGVVTGTPVDGKLAVLFTGQGSQWAGMGRELAETFPVFRDAFEAACEAVDTHLRERPLREVVFDDSALLDQTMYTQGALFAVETALFRLFESWGVRPGLLAGHSIGELAAAHVSGVLDLADAGELVAARGRLMQALPAGGAMVAVQATEDEVAPLLDGTVCVAAVNGPDSVVLSGTEAAVLAVADELAGRGRKTRRLAVSHAFHSPLMEPMLDDFRAVAERLTYRAGSLPVVSTLTGELAALDSPDYWVGQVRNAVRFSDAVTALGAQGASTFLELGPGGALAAMALGTLGGPEQSCVATLRKNGAEVPDVLTALAELHVRGVGVDWTTVLDEPATAVGTVLPTYAFQHQRFWVDVDETAAVSVTPPPAEPIVDRPVQDVLELVGNSSSVDKLAAALEHHHHHH |
| --- |

*RIFS LM-M1 with C-terminal docking domain from 6-deoxyerythronolide B synthase (****RIFS*** ***LM-M1-DD****, pDC42)* **|** A-CPL-KS-AT-DH°-KR-CP1-(DD2)-His_6_

| MRTDLIKPLHVALLENATRFAGKPAFADDHRTVTYGDLEARTRRLAGHLAGLGVRHGDRVAICLGNRVSTVESYFAILRAGAVGVPLNPGSATAELEHPLTDSGATVVVTDAAQAARLRLAPHVELLVTGDDVPEGAHSYDELALSEPAEPAADDLELDEPAWMFYTSGTTGRPKGVVSTQRNCLWSVASCYVPFPGLSDQDRVLWPLPLFHSLSHIACVLSATVVGASVRIADGSSADDVMRLIEAESSTFLAGVPTTYHHLVRAARQRGFSAPSLRIGLAGGAVLGAGLRSEFEETFGVPLIDAYGSTETCGAITMNPPDGARVEGSCGLAVPGVDVRVVDPDTGLDVPAGEEGEVWVSGPNVMLGYHNSPEATAAAMRDGWFRTGDLARRDDAGYFTICGRIKELIIRGGANIHPGEVEAVLRTVDGVADAAVGGVPHDTLGEVPVAYVIPGPTGFDPAALIEKCREQLSAYKVPDRILEVAHIPRTASGKIRRGLLTDEPAQLRYAATEHEEQSRHADESVAAALRARLSGLDERAQCELLEDLVRTQAADVLGQPVPDGRAFRDLGFTSLAIVELRNRLTEHTGLWLPASAVFDHPTPAALAARVRAELLGITQAVAEPVVAADPGEPIAIVGMACRLPGGVASPEDLWRLVAERVDAVSEFPGDRGWDLDSLIDPDRERAGTSYVGQGGFLHDAGEFDAGFFGISPREAVAMDPQQRLLLETSWEALENAGVDPIALKGTDTGVFSGLMGQGYGSGAVAPELEGFVTTGVASSVASGRVSYVLGLEGPAVTVDTACSSSLVAMHLAAQALRQGECSMALAGGVTVMATPGSFVEFSRQRALAPDGRCKAFAAAADGTGWSEGVGVVVLERLSVARERGHRILAVLRGSAVNQDGASNGLTAPNGLSQQRVIRRALAAAGLAPSDVDVVEAHGTGTTLGDPIEAQALLATYGQERKQPLWLGSLKSNIGHAQAAAGVAGVIKMVQALRHETLPPTLHVDKPTLEVDWSAGAIELLTEARAWPRNGRPRRAGVSSFGVSGTNAHLILEEAPAEEPVAAPELPVVPLVVSARSTESLSGQAERLASLLEGDVSLTEVAGALVSRRAVLDERAVVVAGSREEAVTGLRALNTAGSGTPGKVVWVFPGQGTQWAGMGRELLAESPVFAERIAECAAALAPWIDWSLVDVLRGEGDLGRVDVLQPACFAVMVGLAAVWESVGVRPDAVVGHSQGEIAAACVSGALSLEDAAKVVALRSQAIAAELSGRGGMASVALGEDDVVSRLVDGVEVAAVNGPSSVVIAGDAHALDATLEILSGEGIRVRRVAVDYASHTRHVEDIRDTLAETLAGISAQAPAVPFYSTVTSEWVRDAGVLDGGYWYRNLRNQVRFGAAATALLEQGHTVFVEVSAHPVTVQPLSELTGDAIGTLRREDGGLRRLLASMGELFVRGIDVDWTAMVPAAGWVDLPTYAFEHRHYWLEPAEPASAGDPLLGTVVSTPGSDRLTAVAQWSRRAQPWAVDGLVPNAALVEAAIRLGDLAGTPVVGELVVDAPVVLPRRGSREVQLIVGEPGEQRRRPIEVFSREADEPWTRHAHGTLAPAAAAVPEPAAAGDATDVTVAGLRDADRYGIHPALLDAAVRTVVGDDLLPSVWTGVSLLASGATAVTVTPTATGLRLTDPAGQPVLTVESVRGTPFVAEQGTTDALFRVDWPEIPLPTAETADFLPYEATSAEATLSALQAWLADPAETRLAVVTGDCTEPGAAAIWGLVRSAQSEHPGRIVLADLDDPAVLPAVVASGEPQVRVRNGVASVPRLTRVTPRQDARPLDPEGTVLITGGTGTLGALTARHLVTAHGVRHLVLVSRRGEAPELQEELTALGASVAIAACDVADRAQLEAVLRAIPAEHPLTAVIHTAGVLDDGVVTELTPDRLATVRRPKVDAARLLDELTREADLAAFVLFSSAAGVLGNPGQAGYAAANAELDALARQRNSLDLPAVSIAWGYWATVSGMTEHLGDADLRRNQRIGMSGLPADEGMALLDAAIATGGTLVAAKFDVAALRATAKAGGPVPPLLRGLAPLPRRAAAKTASLTERLAGLAETEQAAALLDLVRRHAAEVLGHSGAESVHSGRTFKDAGFDSLTAVELRNRLAAATGLTLSPAMIFDYPKPPALADHLRAKLFGTEVRGEAPSALAGLDALEAALPEVPATEREELVQRLERMLAALRPVAQAADASGTGANPSGDDLGEAGVDELLEALGRELDGDGNSSSVDKLAAALEHHHHHH |
| --- |

*RIFS LM-M1 with C-terminal TEII (a.k.a. RifR) inactivated by S2316A and separated by a* (G_4_S)_8_ linker *(****RIFS*** ***LM-M1-TEII°****, pAM6)* **|** A-CPL-KS-AT-DH°-KR-CP1-(G_4_S)_8_-TEII°-His_6_

| MRTDLIKPLHVALLENATRFAGKPAFADDHRTVTYGDLEARTRRLAGHLAGLGVRHGDRVAICLGNRVSTVESYFAILRAGAVGVPLNPGSATAELEHPLTDSGATVVVTDAAQAARLRLAPHVELLVTGDDVPEGAHSYDELALSEPAEPAADDLELDEPAWMFYTSGTTGRPKGVVSTQRNCLWSVASCYVPFPGLSDQDRVLWPLPLFHSLSHIACVLSATVVGASVRIADGSSADDVMRLIEAESSTFLAGVPTTYHHLVRAARQRGFSAPSLRIGLAGGAVLGAGLRSEFEETFGVPLIDAYGSTETCGAITMNPPDGARVEGSCGLAVPGVDVRVVDPDTGLDVPAGEEGEVWVSGPNVMLGYHNSPEATAAAMRDGWFRTGDLARRDDAGYFTICGRIKELIIRGGANIHPGEVEAVLRTVDGVADAAVGGVPHDTLGEVPVAYVIPGPTGFDPAALIEKCREQLSAYKVPDRILEVAHIPRTASGKIRRGLLTDEPAQLRYAATEHEEQSRHADESVAAALRARLSGLDERAQCELLEDLVRTQAADVLGQPVPDGRAFRDLGFTSLAIVELRNRLTEHTGLWLPASAVFDHPTPAALAARVRAELLGITQAVAEPVVAADPGEPIAIVGMACRLPGGVASPEDLWRLVAERVDAVSEFPGDRGWDLDSLIDPDRERAGTSYVGQGGFLHDAGEFDAGFFGISPREAVAMDPQQRLLLETSWEALENAGVDPIALKGTDTGVFSGLMGQGYGSGAVAPELEGFVTTGVASSVASGRVSYVLGLEGPAVTVDTACSSSLVAMHLAAQALRQGECSMALAGGVTVMATPGSFVEFSRQRALAPDGRCKAFAAAADGTGWSEGVGVVVLERLSVARERGHRILAVLRGSAVNQDGASNGLTAPNGLSQQRVIRRALAAAGLAPSDVDVVEAHGTGTTLGDPIEAQALLATYGQERKQPLWLGSLKSNIGHAQAAAGVAGVIKMVQALRHETLPPTLHVDKPTLEVDWSAGAIELLTEARAWPRNGRPRRAGVSSFGVSGTNAHLILEEAPAEEPVAAPELPVVPLVVSARSTESLSGQAERLASLLEGDVSLTEVAGALVSRRAVLDERAVVVAGSREEAVTGLRALNTAGSGTPGKVVWVFPGQGTQWAGMGRELLAESPVFAERIAECAAALAPWIDWSLVDVLRGEGDLGRVDVLQPACFAVMVGLAAVWESVGVRPDAVVGHSQGEIAAACVSGALSLEDAAKVVALRSQAIAAELSGRGGMASVALGEDDVVSRLVDGVEVAAVNGPSSVVIAGDAHALDATLEILSGEGIRVRRVAVDYASHTRHVEDIRDTLAETLAGISAQAPAVPFYSTVTSEWVRDAGVLDGGYWYRNLRNQVRFGAAATALLEQGHTVFVEVSAHPVTVQPLSELTGDAIGTLRREDGGLRRLLASMGELFVRGIDVDWTAMVPAAGWVDLPTYAFEHRHYWLEPAEPASAGDPLLGTVVSTPGSDRLTAVAQWSRRAQPWAVDGLVPNAALVEAAIRLGDLAGTPVVGELVVDAPVVLPRRGSREVQLIVGEPGEQRRRPIEVFSREADEPWTRHAHGTLAPAAAAVPEPAAAGDATDVTVAGLRDADRYGIHPALLDAAVRTVVGDDLLPSVWTGVSLLASGATAVTVTPTATGLRLTDPAGQPVLTVESVRGTPFVAEQGTTDALFRVDWPEIPLPTAETADFLPYEATSAEATLSALQAWLADPAETRLAVVTGDCTEPGAAAIWGLVRSAQSEHPGRIVLADLDDPAVLPAVVASGEPQVRVRNGVASVPRLTRVTPRQDARPLDPEGTVLITGGTGTLGALTARHLVTAHGVRHLVLVSRRGEAPELQEELTALGASVAIAACDVADRAQLEAVLRAIPAEHPLTAVIHTAGVLDDGVVTELTPDRLATVRRPKVDAARLLDELTREADLAAFVLFSSAAGVLGNPGQAGYAAANAELDALARQRNSLDLPAVSIAWGYWATVSGMTEHLGDADLRRNQRIGMSGLPADEGMALLDAAIATGGTLVAAKFDVAALRATAKAGGPVPPLLRGLAPLPRRAAAKTASLTERLAGLAETEQAAALLDLVRRHAAEVLGHSGAESVHSGRTFKDAGFDSLTAVELRNRLAAATGLTLSPAMIFDYPKPPALADHLRAKLFGSAASGGGGSGGGGSGGGGSGGGGSGGGGSGGGGSGGGGSGGGGSHRPEAEKWLRRFERAPDARARLVCLPHAGGSASFFFPLAKALAPAVEVLAVQYPGRQDRRHEPPVDSIGGLTNRLLEVLRPFGDRPLALFGHAMGAIIGYELALRMPEAGLPAPVHLFASGRRAPSRYRDDDVRGASDERLVAELRKLGGSDAAMLADPELLAMVLPAIRSDYRAVETYRHEPGRRVDCPVTVFTGDHDPRVSVGEARAWEEHTTGPADLRVLPGGHFFLVDQAAPMIATMTEKLAGPALTGSTGGNSGNSSSVDKLAAALEHHHHHH |
| --- |

*RIFS M1 with N- and C-terminal docking domains from 6-deoxyerythronolide B synthase (****RIFS*** ***M1-DD****, pDC43)* **|** (DD3)-KS-AT-DH°-KR-CP1-(DD2)-His_6_

| MASTDSEKVAEYLRRATLDLRAARQRIRELEGEPIAIVGMACRLPGGVASPEDLWRLVAERVDAVSEFPGDRGWDLDSLIDPDRERAGTSYVGQGGFLHDAGEFDAGFFGISPREAVAMDPQQRLLLETSWEALENAGVDPIALKGTDTGVFSGLMGQGYGSGAVAPELEGFVTTGVASSVASGRVSYVLGLEGPAVTVDTACSSSLVAMHLAAQALRQGECSMALAGGVTVMATPGSFVEFSRQRALAPDGRCKAFAAAADGTGWSEGVGVVVLERLSVARERGHRILAVLRGSAVNQDGASNGLTAPNGLSQQRVIRRALAAAGLAPSDVDVVEAHGTGTTLGDPIEAQALLATYGQERKQPLWLGSLKSNIGHAQAAAGVAGVIKMVQALRHETLPPTLHVDKPTLEVDWSAGAIELLTEARAWPRNGRPRRAGVSSFGVSGTNAHLILEEAPAEEPVAAPELPVVPLVVSARSTESLSGQAERLASLLEGDVSLTEVAGALVSRRAVLDERAVVVAGSREEAVTGLRALNTAGSGTPGKVVWVFPGQGTQWAGMGRELLAESPVFAERIAECAAALAPWIDWSLVDVLRGEGDLGRVDVLQPACFAVMVGLAAVWESVGVRPDAVVGHSQGEIAAACVSGALSLEDAAKVVALRSQAIAAELSGRGGMASVALGEDDVVSRLVDGVEVAAVNGPSSVVIAGDAHALDATLEILSGEGIRVRRVAVDYASHTRHVEDIRDTLAETLAGISAQAPAVPFYSTVTSEWVRDAGVLDGGYWYRNLRNQVRFGAAATALLEQGHTVFVEVSAHPVTVQPLSELTGDAIGTLRREDGGLRRLLASMGELFVRGIDVDWTAMVPAAGWVDLPTYAFEHRHYWLEPAEPASAGDPLLGTVVSTPGSDRLTAVAQWSRRAQPWAVDGLVPNAALVEAAIRLGDLAGTPVVGELVVDAPVVLPRRGSREVQLIVGEPGEQRRRPIEVFSREADEPWTRHAHGTLAPAAAAVPEPAAAGDATDVTVAGLRDADRYGIHPALLDAAVRTVVGDDLLPSVWTGVSLLASGATAVTVTPTATGLRLTDPAGQPVLTVESVRGTPFVAEQGTTDALFRVDWPEIPLPTAETADFLPYEATSAEATLSALQAWLADPAETRLAVVTGDCTEPGAAAIWGLVRSAQSEHPGRIVLADLDDPAVLPAVVASGEPQVRVRNGVASVPRLTRVTPRQDARPLDPEGTVLITGGTGTLGALTARHLVTAHGVRHLVLVSRRGEAPELQEELTALGASVAIAACDVADRAQLEAVLRAIPAEHPLTAVIHTAGVLDDGVVTELTPDRLATVRRPKVDAARLLDELTREADLAAFVLFSSAAGVLGNPGQAGYAAANAELDALARQRNSLDLPAVSIAWGYWATVSGMTEHLGDADLRRNQRIGMSGLPADEGMALLDAAIATGGTLVAAKFDVAALRATAKAGGPVPPLLRGLAPLPRRAAAKTASLTERLAGLAETEQAAALLDLVRRHAAEVLGHSGAESVHSGRTFKDAGFDSLTAVELRNRLAAATGLTLSPAMIFDYPKPPALADHLRAKLFGTEVRGEAPSALAGLDALEAALPEVPATEREELVQRLERMLAALRPVAQAADASGTGANPSGDDLGEAGVDELLEALGRELDGDGNSSSVDKLAAALEHHHHHH |
| --- |

*RIFS M1 with N-terminal docking domain from 6-deoxyerythronolide B synthase and C-terminal TEII (a.k.a. RifR) separated by a* (G_4_S)_2_ linker *(****RIFS*** ***M1-TEII****, pDC85)* **|** (DD3)-KS-AT-DH°-KR-CP1-(G_4_S)_2_-TEII-His_6_

| MASTDSEKVAEYLRRATLDLRAARQRIRELEGEPIAIVGMACRLPGGVASPEDLWRLVAERVDAVSEFPGDRGWDLDSLIDPDRERAGTSYVGQGGFLHDAGEFDAGFFGISPREAVAMDPQQRLLLETSWEALENAGVDPIALKGTDTGVFSGLMGQGYGSGAVAPELEGFVTTGVASSVASGRVSYVLGLEGPAVTVDTACSSSLVAMHLAAQALRQGECSMALAGGVTVMATPGSFVEFSRQRALAPDGRCKAFAAAADGTGWSEGVGVVVLERLSVARERGHRILAVLRGSAVNQDGASNGLTAPNGLSQQRVIRRALAAAGLAPSDVDVVEAHGTGTTLGDPIEAQALLATYGQERKQPLWLGSLKSNIGHAQAAAGVAGVIKMVQALRHETLPPTLHVDKPTLEVDWSAGAIELLTEARAWPRNGRPRRAGVSSFGVSGTNAHLILEEAPAEEPVAAPELPVVPLVVSARSTESLSGQAERLASLLEGDVSLTEVAGALVSRRAVLDERAVVVAGSREEAVTGLRALNTAGSGTPGKVVWVFPGQGTQWAGMGRELLAESPVFAERIAECAAALAPWIDWSLVDVLRGEGDLGRVDVLQPACFAVMVGLAAVWESVGVRPDAVVGHSQGEIAAACVSGALSLEDAAKVVALRSQAIAAELSGRGGMASVALGEDDVVSRLVDGVEVAAVNGPSSVVIAGDAHALDATLEILSGEGIRVRRVAVDYASHTRHVEDIRDTLAETLAGISAQAPAVPFYSTVTSEWVRDAGVLDGGYWYRNLRNQVRFGAAATALLEQGHTVFVEVSAHPVTVQPLSELTGDAIGTLRREDGGLRRLLASMGELFVRGIDVDWTAMVPAAGWVDLPTYAFEHRHYWLEPAEPASAGDPLLGTVVSTPGSDRLTAVAQWSRRAQPWAVDGLVPNAALVEAAIRLGDLAGTPVVGELVVDAPVVLPRRGSREVQLIVGEPGEQRRRPIEVFSREADEPWTRHAHGTLAPAAAAVPEPAAAGDATDVTVAGLRDADRYGIHPALLDAAVRTVVGDDLLPSVWTGVSLLASGATAVTVTPTATGLRLTDPAGQPVLTVESVRGTPFVAEQGTTDALFRVDWPEIPLPTAETADFLPYEATSAEATLSALQAWLADPAETRLAVVTGDCTEPGAAAIWGLVRSAQSEHPGRIVLADLDDPAVLPAVVASGEPQVRVRNGVASVPRLTRVTPRQDARPLDPEGTVLITGGTGTLGALTARHLVTAHGVRHLVLVSRRGEAPELQEELTALGASVAIAACDVADRAQLEAVLRAIPAEHPLTAVIHTAGVLDDGVVTELTPDRLATVRRPKVDAARLLDELTREADLAAFVLFSSAAGVLGNPGQAGYAAANAELDALARQRNSLDLPAVSIAWGYWATVSGMTEHLGDADLRRNQRIGMSGLPADEGMALLDAAIATGGTLVAAKFDVAALRATAKAGGPVPPLLRGLAPLPRRAAAKTASLTERLAGLAETEQAAALLDLVRRHAAEVLGHSGAESVHSGRTFKDAGFDSLTAVELRNRLAAATGLTLSPAMIFDYPKPPALADHLRAKLFGSGGGGSGGGGSHRPEAEKWLRRFERAPDARARLVCLPHAGGSASFFFPLAKALAPAVEVLAVQYPGRQDRRHEPPVDSIGGLTNRLLEVLRPFGDRPLALFGHSMGAIIGYELALRMPEAGLPAPVHLFASGRRAPSRYRDDDVRGASDERLVAELRKLGGSDAAMLADPELLAMVLPAIRSDYRAVETYRHEPGRRVDCPVTVFTGDHDPRVSVGEARAWEEHTTGPADLRVLPGGHFFLVDQAAPMIATMTEKLAGPALTGSTGGNSGNSSSVDKLAAALEHHHHHH |
| --- |

*RIFS M1 with N-terminal docking domain from 6-deoxyerythronolide B synthase and C-terminal TEII (a.k.a. RifR) inactivated by S2316A and separated by a* (G_4_S)_8_ linker *(****RIFS*** ***M1-TEII°****, pDC147)* **|** (DD3)-KS-AT-DH°-KR-CP1-(G_4_S)_8_-TEII°-His_6_

| MASTDSEKVAEYLRRATLDLRAARQRIRELEGEPIAIVGMACRLPGGVASPEDLWRLVAERVDAVSEFPGDRGWDLDSLIDPDRERAGTSYVGQGGFLHDAGEFDAGFFGISPREAVAMDPQQRLLLETSWEALENAGVDPIALKGTDTGVFSGLMGQGYGSGAVAPELEGFVTTGVASSVASGRVSYVLGLEGPAVTVDTACSSSLVAMHLAAQALRQGECSMALAGGVTVMATPGSFVEFSRQRALAPDGRCKAFAAAADGTGWSEGVGVVVLERLSVARERGHRILAVLRGSAVNQDGASNGLTAPNGLSQQRVIRRALAAAGLAPSDVDVVEAHGTGTTLGDPIEAQALLATYGQERKQPLWLGSLKSNIGHAQAAAGVAGVIKMVQALRHETLPPTLHVDKPTLEVDWSAGAIELLTEARAWPRNGRPRRAGVSSFGVSGTNAHLILEEAPAEEPVAAPELPVVPLVVSARSTESLSGQAERLASLLEGDVSLTEVAGALVSRRAVLDERAVVVAGSREEAVTGLRALNTAGSGTPGKVVWVFPGQGTQWAGMGRELLAESPVFAERIAECAAALAPWIDWSLVDVLRGEGDLGRVDVLQPACFAVMVGLAAVWESVGVRPDAVVGHSQGEIAAACVSGALSLEDAAKVVALRSQAIAAELSGRGGMASVALGEDDVVSRLVDGVEVAAVNGPSSVVIAGDAHALDATLEILSGEGIRVRRVAVDYASHTRHVEDIRDTLAETLAGISAQAPAVPFYSTVTSEWVRDAGVLDGGYWYRNLRNQVRFGAAATALLEQGHTVFVEVSAHPVTVQPLSELTGDAIGTLRREDGGLRRLLASMGELFVRGIDVDWTAMVPAAGWVDLPTYAFEHRHYWLEPAEPASAGDPLLGTVVSTPGSDRLTAVAQWSRRAQPWAVDGLVPNAALVEAAIRLGDLAGTPVVGELVVDAPVVLPRRGSREVQLIVGEPGEQRRRPIEVFSREADEPWTRHAHGTLAPAAAAVPEPAAAGDATDVTVAGLRDADRYGIHPALLDAAVRTVVGDDLLPSVWTGVSLLASGATAVTVTPTATGLRLTDPAGQPVLTVESVRGTPFVAEQGTTDALFRVDWPEIPLPTAETADFLPYEATSAEATLSALQAWLADPAETRLAVVTGDCTEPGAAAIWGLVRSAQSEHPGRIVLADLDDPAVLPAVVASGEPQVRVRNGVASVPRLTRVTPRQDARPLDPEGTVLITGGTGTLGALTARHLVTAHGVRHLVLVSRRGEAPELQEELTALGASVAIAACDVADRAQLEAVLRAIPAEHPLTAVIHTAGVLDDGVVTELTPDRLATVRRPKVDAARLLDELTREADLAAFVLFSSAAGVLGNPGQAGYAAANAELDALARQRNSLDLPAVSIAWGYWATVSGMTEHLGDADLRRNQRIGMSGLPADEGMALLDAAIATGGTLVAAKFDVAALRATAKAGGPVPPLLRGLAPLPRRAAAKTASLTERLAGLAETEQAAALLDLVRRHAAEVLGHSGAESVHSGRTFKDAGFDSLTAVELRNRLAAATGLTLSPAMIFDYPKPPALADHLRAKLFGSAASGGGGSGGGGSGGGGSGGGGSGGGGSGGGGSGGGGSGGGGSHRPEAEKWLRRFERAPDARARLVCLPHAGGSASFFFPLAKALAPAVEVLAVQYPGRQDRRHEPPVDSIGGLTNRLLEVLRPFGDRPLALFGHAMGAIIGYELALRMPEAGLPAPVHLFASGRRAPSRYRDDDVRGASDERLVAELRKLGGSDAAMLADPELLAMVLPAIRSDYRAVETYRHEPGRRVDCPVTVFTGDHDPRVSVGEARAWEEHTTGPADLRVLPGGHFFLVDQAAPMIATMTEKLAGPALTGSTGGNSGNSSSVDKLAAALEHHHHHH |
| --- |

*RIFS M1 with N- and C-terminal docking domains from the 6-deoxyerythronolide B synthase and a 38-residue deletion (Δ1588–1625) within the C-terminal docking domain corresponding to its dimeric α-helical fragment (****RIFS*** ***M1-ΔDD****, pDC155)* **|** (DD3)-KS-AT-DH°-KR-CP1-(ΔDD2)-His_6_

| MASTDSEKVAEYLRRATLDLRAARQRIRELEGEPIAIVGMACRLPGGVASPEDLWRLVAERVDAVSEFPGDRGWDLDSLIDPDRERAGTSYVGQGGFLHDAGEFDAGFFGISPREAVAMDPQQRLLLETSWEALENAGVDPIALKGTDTGVFSGLMGQGYGSGAVAPELEGFVTTGVASSVASGRVSYVLGLEGPAVTVDTACSSSLVAMHLAAQALRQGECSMALAGGVTVMATPGSFVEFSRQRALAPDGRCKAFAAAADGTGWSEGVGVVVLERLSVARERGHRILAVLRGSAVNQDGASNGLTAPNGLSQQRVIRRALAAAGLAPSDVDVVEAHGTGTTLGDPIEAQALLATYGQERKQPLWLGSLKSNIGHAQAAAGVAGVIKMVQALRHETLPPTLHVDKPTLEVDWSAGAIELLTEARAWPRNGRPRRAGVSSFGVSGTNAHLILEEAPAEEPVAAPELPVVPLVVSARSTESLSGQAERLASLLEGDVSLTEVAGALVSRRAVLDERAVVVAGSREEAVTGLRALNTAGSGTPGKVVWVFPGQGTQWAGMGRELLAESPVFAERIAECAAALAPWIDWSLVDVLRGEGDLGRVDVLQPACFAVMVGLAAVWESVGVRPDAVVGHSQGEIAAACVSGALSLEDAAKVVALRSQAIAAELSGRGGMASVALGEDDVVSRLVDGVEVAAVNGPSSVVIAGDAHALDATLEILSGEGIRVRRVAVDYASHTRHVEDIRDTLAETLAGISAQAPAVPFYSTVTSEWVRDAGVLDGGYWYRNLRNQVRFGAAATALLEQGHTVFVEVSAHPVTVQPLSELTGDAIGTLRREDGGLRRLLASMGELFVRGIDVDWTAMVPAAGWVDLPTYAFEHRHYWLEPAEPASAGDPLLGTVVSTPGSDRLTAVAQWSRRAQPWAVDGLVPNAALVEAAIRLGDLAGTPVVGELVVDAPVVLPRRGSREVQLIVGEPGEQRRRPIEVFSREADEPWTRHAHGTLAPAAAAVPEPAAAGDATDVTVAGLRDADRYGIHPALLDAAVRTVVGDDLLPSVWTGVSLLASGATAVTVTPTATGLRLTDPAGQPVLTVESVRGTPFVAEQGTTDALFRVDWPEIPLPTAETADFLPYEATSAEATLSALQAWLADPAETRLAVVTGDCTEPGAAAIWGLVRSAQSEHPGRIVLADLDDPAVLPAVVASGEPQVRVRNGVASVPRLTRVTPRQDARPLDPEGTVLITGGTGTLGALTARHLVTAHGVRHLVLVSRRGEAPELQEELTALGASVAIAACDVADRAQLEAVLRAIPAEHPLTAVIHTAGVLDDGVVTELTPDRLATVRRPKVDAARLLDELTREADLAAFVLFSSAAGVLGNPGQAGYAAANAELDALARQRNSLDLPAVSIAWGYWATVSGMTEHLGDADLRRNQRIGMSGLPADEGMALLDAAIATGGTLVAAKFDVAALRATAKAGGPVPPLLRGLAPLPRRAAAKTASLTERLAGLAETEQAAALLDLVRRHAAEVLGHSGAESVHSGRTFKDAGFDSLTAVELRNRLAAATGLTLSPAMIFDYPKPPALADHLRAKLFGTEVRGEAVAQAADASGTGANPSGDDLGEAGVDELLEALGRELDGDGNSSSVDKLAAALEHHHHHH |
| --- |

*NOCAPS M1 with N- and C-terminal docking domains from the 6-deoxyerythronolide B synthase (****NOCAPS*** ***M1-DD****, pDC57)* **|** (DD3)-KS-AT-DH-KR-CP1-(DD2)-His_6_

| MASTDSEKVAEYLRRATLDLRAARQRIRELEQEPIAVIGMGCRFPGGAESPQQYWELLSSGRGAIVEVPENRWPVADSAPRRARRRAGLLAGPIDAMDTEFLGIAPREAASMDPQQRLVLEVAWEAMEDAALAPNGPAAARTGVFLGVSWQEYQRTITPDWVNSVDAHTLTGTMSSIVAGRVSYVLGLRGPAVAIDTACSSSLVAVHQACRSLRAGECEVALAGGVNLLQSELTSEALARMGALSPDGHCRPFDARANGYVRGEGAGVVVLKPLSKALADGDPIRALIRGSSVNHDGRSMGLTAPNPTAQRELLRDALADAGCAADQVGYVETHGTGTPLGDPIEIEALSQVLGAPRADGSVCLLGSVKSQIGHLEAAAGIAGLIKAVLQLEHERIPRQHDFATMNPKIALDGTALAVPTDDVAWPRTDRPRLAGVSSFGFAGTNAHVLLEQAPEPSPTVDAEQPELLALSARTASALDALSRRYVEQLDTTQSPLADIAATAALGRSHFAHRRAVVARTVAEAARKLAEPPGRSTDLDSGKPRVAMLFTGQGAQYAGMGAALDRGYPAFRAALDRCDDILGPLPGGLRLRSVLFEANDEVLSRTEYAQPALFAIEWACAELWATFGVRPDIVLGHSLGELVAATVAGVLDLETGLRLAAERGRLMQSVARPGAMAAVFGKPEEFAALLDELAAEVAVAAVNGPGQFVVSGTAEAVRRILEDAARRSSRSVKLPGSTAFHSPLLDPMLDEFETRVAELVPAPRAAVIPLVSNVTGTRIEAALDAAYWRAHARGTVRFADGVRSVADFGVDAYLEVGPHPVLLEFGRQADPDALWLPSMRRTSTDDEVLLTGLGEMYCAGADIDWTAVIPPRARRRNGLPTYPFERERITVPIAGASSNTGYLAEHRFQDRAVVPAAYLALRALRAAGGANRELVDFVAARPVPLEELADPIVSGADGHADVRFHVGNGARTAASGSVRAATSKRPRDSNDLAEARRASSTEWEVADFYRRCADAGLSFGPRFRWITRLHVTGSSVLAELTRPAELAGEPDAALACLLDAAVQTGLALTARHSHAAQLPVGIGRLRVSGGETAAKAWALAERTGRTVRITVFDTAGAVVCEFDDVWFAESGAAGQSQDRDLLSGLVRVPVWEPAAASATGRGPDATLVIGVGQQADRLAAALRAAGTDAVAVERTTDPAAWPDDNRAVVYLADTEADDSADTARTETEFVLGVVQAMARAATAGPLYLVTMGATGALPGERVRPAAATLWGLGAVVRTELPELRCRLIDLPVGARCDDRRLIDELASSAADFVVALRGRQRYVTRTESFDDAGEAPRLERDAAYLITGGRGALGLTAARTLAECGAGTVVLVGRTDPDAHAQREMARIAALGTRVETATVDVADRNALSGLLARFGGELPRLAGVVHCAGVLDDAMLIEQTPAHVDRVFAGKVAGAWHLHELTAEHSLELFVLFSSVSATVGTAGQGNYAAANAYLDALARLRLAEGLPAVSIAWGPWAGTGMAGGLSDAARRAWERRGIAELAAEDGARALRSLLAAEGVVAVFPAAADASTRQEAVADADEQDMEAPRAAADLLELVRTHAMTALGRSAAVSDRTPLRELGLDSMMAVELRNALSADLGVRLPATLLFDHPTVAAVSEEIARLSAREGGATEVRGEAPSALAGLDALEAALPEVPATEREELVQRLERMLAALRPVAQAADASGTGANPSGDDLGEAGVDELLEALGRELDGDGNSSSVDKLAAALEHHHHHH |
| --- |

*NOCAPS M1 with C-terminal TEII derived from NocapC (****NOCAPS*** ***M1-TEII****, pDC81)* **|** KS-AT-DH-KR-CP1-TEII-His_6_

| MADDGYDLRVLLTRALRRIQELETGPRQEPIAVIGMGCRFPGGAESPQQYWELLSSGRGAIVEVPENRWPVADSAPRRARRRAGLLAGPIDAMDTEFLGIAPREAASMDPQQRLVLEVAWEAMEDAALAPNGPAAARTGVFLGVSWQEYQRTITPDWVNSVDAHTLTGTMSSIVAGRVSYVLGLRGPAVAIDTACSSSLVAVHQACRSLRAGECEVALAGGVNLLQSELTSEALARMGALSPDGHCRPFDARANGYVRGEGAGVVVLKPLSKALADGDPIRALIRGSSVNHDGRSMGLTAPNPTAQRELLRDALADAGCAADQVGYVETHGTGTPLGDPIEIEALSQVLGAPRADGSVCLLGSVKSQIGHLEAAAGIAGLIKAVLQLEHERIPRQHDFATMNPKIALDGTALAVPTDDVAWPRTDRPRLAGVSSFGFAGTNAHVLLEQAPEPSPTVDAEQPELLALSARTASALDALSRRYVEQLDTTQSPLADIAATAALGRSHFAHRRAVVARTVAEAARKLAEPPGRSTDLDSGKPRVAMLFTGQGAQYAGMGAALDRGYPAFRAALDRCDDILGPLPGGLRLRSVLFEANDEVLSRTEYAQPALFAIEWACAELWATFGVRPDIVLGHSLGELVAATVAGVLDLETGLRLAAERGRLMQSVARPGAMAAVFGKPEEFAALLDELAAEVAVAAVNGPGQFVVSGTAEAVRRILEDAARRSSRSVKLPGSTAFHSPLLDPMLDEFETRVAELVPAPRAAVIPLVSNVTGTRIEAALDAAYWRAHARGTVRFADGVRSVADFGVDAYLEVGPHPVLLEFGRQADPDALWLPSMRRTSTDDEVLLTGLGEMYCAGADIDWTAVIPPRARRRNGLPTYPFERERITVPIAGASSNTGYLAEHRFQDRAVVPAAYLALRALRAAGGANRELVDFVAARPVPLEELADPIVSGADGHADVRFHVGNGARTAASGSVRAATSKRPRDSNDLAEARRASSTEWEVADFYRRCADAGLSFGPRFRWITRLHVTGSSVLAELTRPAELAGEPDAALACLLDAAVQTGLALTARHSHAAQLPVGIGRLRVSGGETAAKAWALAERTGRTVRITVFDTAGAVVCEFDDVWFAESGAAGQSQDRDLLSGLVRVPVWEPAAASATGRGPDATLVIGVGQQADRLAAALRAAGTDAVAVERTTDPAAWPDDNRAVVYLADTEADDSADTARTETEFVLGVVQAMARAATAGPLYLVTMGATGALPGERVRPAAATLWGLGAVVRTELPELRCRLIDLPVGARCDDRRLIDELASSAADFVVALRGRQRYVTRTESFDDAGEAPRLERDAAYLITGGRGALGLTAARTLAECGAGTVVLVGRTDPDAHAQREMARIAALGTRVETATVDVADRNALSGLLARFGGELPRLAGVVHCAGVLDDAMLIEQTPAHVDRVFAGKVAGAWHLHELTAEHSLELFVLFSSVSATVGTAGQGNYAAANAYLDALARLRLAEGLPAVSIAWGPWAGTGMAGGLSDAARRAWERRGIAELAAEDGARALRSLLAAEGVVAVFPAAADASTRQEAVADADEQDMEAPRAAADLLELVRTHAMTALGRSAAVSDRTPLRELGLDSMMAVELRNALSADLGVRLPATLLFDHPTVAAVSEEIARLSAREGGAAGSRDREAKGPAAPLLPATLTQTTTQPARATPPSDDTGVLTIHRRTAGTPSLRLFCWPFAGGKAAAYTPWRRHLPDWVELCVIELPARQRHLAQTPIRRFTDLVDASLAQVLPLTDLPFAFFGHSLGALTAYEVARGLPAGIEPRALFLGAVAAPHLPRPGRLSGLPDHEFIAAVGHYGGIPPEVRDTPEVMALFLPALRSDFEIFDDYRFAPAAAPSCPAHLFGGRDDRQVATTQLEAWADVLPGLRSTELMPGGHFFLVEHREALLGSLTDKLTAVHPDVVPAGNSSSVDKLAAALEHHHHHH |
| --- |

*F_ab_ 1B2 heavy chain with a C-terminal His_6_ and FLAG tag*

| MAEVQLVQSGGGLVQPGRSLRLSCTASGFTFGDYAMSWVRQAPGKGLEWVGFIRSKAYGGTTEYAASVKGRFTISRDDSKSIAYLQMNSLKTEDTAVYYCTRGGTLFDYWGQGTLVTVSSASTKGPSVFPLAPSSKSTSGGTAALGCLVKDYFPEPVTVSWNSGALTSGVHTFPAVLQSSGLYSLSSVVTVPSSSLGTQTYICNVNHKPSNTKVDKKVEPKSCAALVPRGSAHHHHHHAADYKDDDDKA |
| --- |

*F_ab_ 1B2 light chain*

| LFAIPLVVPFYSHSALDVVMTQSPLSLPVTPGEPASISCRSSQSLLHSNGYNYLDWYLQKPGQSPQLLIYLGSNRASGVPDRFSGSGSGTDFTLKISRVEAEDVGVYYCMQSLQTPRLTFGPGTKVDIKRTVAAPSVFIFPPSDEQLKSGTASVVCLLNNFYPRGAKVQWKVDNALQSGNSQESVTEQDSKDSTYSLSSTLTLSKADYEKHKVYACEVTHQGLSSPVTKSFNRGEC |
| --- |

**Caption for Supporting Movie**

The left half shows a linear interpolation between the *transacylation-mode* (PDB 9PAT) and *elongation-mode* (PDB 9PAV) structures generated via *morph* in ChimeraX. The right half displays results from Gaussian mixture model (GMM) based heterogeneity analysis in EMAN2 employed to extract information about continuous motion in the M1-DD-1B2 single-particle cryo-EM dataset (Fig. S8 and Table S4)(24). For the GMM-based analysis, a volume series reconstructed from 10,000 particles was generated along the first eigenvector in the conformational space to visualize DH° dimer motion and its associated CP occupancy. The left and right halves of the 100-frame movie are synchronized such that frames 0 (0 s), 50 (2 s), and 100 (4 s) correspond to a 23° relative angle of the DH° dimer with respect to the pseudo-C2 axis of module symmetry, whereas frames 25 (1 s) and 75 (3 s) correspond to a 0° relative angle (in accordance with Fig. 3E). The movie displays a total of 2 oscillations of the DH° dimer from 23° to 0°, wherein density for the CP domain bound to the KS was weakest and strongest at 23° and 0°, respectively.

**Supporting References**

1. Admiraal, S. J., Khosla, C., and Walsh, C. T. (2003) A Switch for the transfer of substrate between nonribosomal peptide and polyketide modules of the rifamycin synthetase assembly line. *J. Am. Chem. Soc.* **125**, 13664–13665

2. Gay, D., You, Y.-O., Keatinge-Clay, A., and Cane, D. E. (2013) Structure and Stereospecificity of the Dehydratase Domain from the Terminal Module of the Rifamycin Polyketide Synthase. *Biochemistry*. **52**, 8916–8928

3. Kao, C. M., Pieper, R., Cane, D. E., and Khosla, C. (1996) Evidence for two catalytically independent clusters of active sites in a functional modular polyketide synthase. *Biochemistry*. **35**, 12363–12368

4. Cogan, D. P., Zhang, K., Li, X., Li, S., Pintilie, G. D., Roh, S.-H., Craik, C. S., Chiu, W., and Khosla, C. (2021) Mapping the catalytic conformations of an assembly-line polyketide synthase module. *Science*. **374**, 729–734

5. Lowry, B., Li, X., Robbins, T., Cane, D. E., and Khosla, C. (2016) A turnstile mechanism for the controlled growth of biosynthetic intermediates on assembly line polyketide synthases. *ACS Cent. Sci.* **2**, 14–20

6. Dorrestein, P. C., Bumpus, S. B., Calderone, C. T., Garneau-Tsodikova, S., Aron, Z. D., Straight, P. D., Kolter, R., Walsh, C. T., and Kelleher, N. L. (2006) Facile detection of acyl and peptidyl intermediates on thiotemplate carrier domains via phosphopantetheinyl elimination reactions during tandem mass spectrometry. *Biochemistry*. **45**, 12756–12766

7. Pfeifer, B. A., Admiraal, S. J., Gramajo, H., Cane, D. E., and Khosla, C. (2001) Biosynthesis of complex polyketides in a metabolically engineered strain of *E. coli*. *Science*. **291**, 1790–1792

8. Scheres, S. H. W. (2012) RELION: Implementation of a Bayesian approach to cryo-EM structure determination. *J. Struct. Biol.* **180**, 519–530

9. Punjani, A., Rubinstein, J. L., Fleet, D. J., and Brubaker, M. A. (2017) cryoSPARC: algorithms for rapid unsupervised cryo-EM structure determination. *Nat. Methods*. **14**, 290–296

10. Henderson, R., Sali, A., Baker, M. L., Carragher, B., Devkota, B., Downing, K. H., Egelman, E. H., Feng, Z., Frank, J., Grigorieff, N., Jiang, W., Ludtke, S. J., Medalia, O., Penczek, P. A., Rosenthal, P. B., Rossmann, M. G., Schmid, M. F., Schröder, G. F., Steven, A. C., Stokes, D. L., Westbrook, J. D., Wriggers, W., Yang, H., Young, J., Berman, H. M., Chiu, W., Kleywegt, G. J., and Lawson, C. L. (2012) Outcome of the first electron microscopy validation task force meeting. *Structure*. **20**, 205–214

11. Afonine, P. V, Poon, B. K., Read, R. J., Sobolev, O. V, Terwilliger, T. C., Urzhumtsev, A., and Adams, P. D. (2018) Real-space refinement in PHENIX for cryo-EM and crystallography. *Acta Crystallogr. Sect. D, Struct. Biol.* **74**, 531–544

12. Pintilie, G., Zhang, K., Su, Z., Li, S., Schmid, M. F., and Chiu, W. (2020) Measurement of atom resolvability in cryo-EM maps with Q-scores. *Nat. Methods*. **17**, 328–334

13. Abramson, J., Adler, J., Dunger, J., Evans, R., Green, T., Pritzel, A., Ronneberger, O., Willmore, L., Ballard, A. J., Bambrick, J., Bodenstein, S. W., Evans, D. A., Hung, C.-C., O’Neill, M., Reiman, D., Tunyasuvunakool, K., Wu, Z., Žemgulytė, A., Arvaniti, E., Beattie, C., Bertolli, O., Bridgland, A., Cherepanov, A., Congreve, M., Cowen-Rivers, A. I., Cowie, A., Figurnov, M., Fuchs, F. B., Gladman, H., Jain, R., Khan, Y. A., Low, C. M. R., Perlin, K., Potapenko, A., Savy, P., Singh, S., Stecula, A., Thillaisundaram, A., Tong, C., Yakneen, S., Zhong, E. D., Zielinski, M., Žídek, A., Bapst, V., Kohli, P., Jaderberg, M., Hassabis, D., and Jumper, J. M. (2024) Accurate structure prediction of biomolecular interactions with AlphaFold 3. *Nature*. **630**, 493–500

14. van Kempen, M., Kim, S. S., Tumescheit, C., Mirdita, M., Lee, J., Gilchrist, C. L. M., Söding, J., and Steinegger, M. (2024) Fast and accurate protein structure search with Foldseek. *Nat. Biotechnol.* **42**, 243–246

15. Altschul, S. F., Gish, W., Miller, W., Myers, E. W., and Lipman, D. J. (1990) Basic local alignment search tool. *J. Mol. Biol.* **215**, 403–10

16. Crooks, G. E., Hon, G., Chandonia, J.-M., and Brenner, S. E. (2004) WebLogo: a sequence logo generator. *Genome Res.* **14**, 1188–1190

17. Ashkenazy, H., Abadi, S., Martz, E., Chay, O., Mayrose, I., Pupko, T., and Ben-Tal, N. (2016) ConSurf 2016: an improved methodology to estimate and visualize evolutionary conservation in macromolecules. *Nucleic Acids Res.* **44**, W344–W350

18. Cogan, D. P., Soohoo, A. M., Chen, M., Liu, Y., Brodsky, K. L., and Khosla, C. (2025) Structural basis for intermodular communication in assembly-line polyketide biosynthesis. *Nat. Chem. Biol.* **21**, 876–882

19. Yuet, K. P., Liu, C. W., Lynch, S. R., Kuo, J., Michaels, W., Lee, R. B., McShane, A. E., Zhong, B. L., Fischer, C. R., and Khosla, C. (2020) Complete Reconstitution and Deorphanization of the 3 MDa Nocardiosis-Associated Polyketide Synthase. *J. Am. Chem. Soc.* **142**, 5952–5957

20. Admiraal, S. J., Khosla, C., and Walsh, C. T. (2002) The loading and initial elongation modules of rifamycin synthetase collaborate to produce mixed aryl ketide products. *Biochemistry*. **41**, 5313–5324

21. Miyazawa, T., Hirsch, M., Zhang, Z., and Keatinge-Clay, A. T. (2020) An in vitro platform for engineering and harnessing modular polyketide synthases. *Nat. Commun.* **11**, 80

22. Gokhale, R. S., Tsuji, S. Y., Cane, D. E., and Khosla, C. (1999) Dissecting and exploiting intermodular communication in polyketide synthases. *Science*. **284**, 482–485

23. Li, X., Sevillano, N., La Greca, F., Deis, L., Liu, Y.-C., Deller, M. C., Mathews, I. I., Matsui, T., Cane, D. E., Craik, C. S., and Khosla, C. (2018) Structure–Function Analysis of the Extended Conformation of a Polyketide Synthase Module. *J. Am. Chem. Soc.* **140**, 6518–6521

24. Chen, M., Schmid, M. F., and Chiu, W. (2024) Improving resolution and resolvability of single-particle cryoEM structures using Gaussian mixture models. *Nat. Methods*. **21**, 37–40
